# Design, synthesis and antiproliferative, apoptotic, and immunomodulatory properties of new heteroaryl pyridine-linked 1,2,4-oxadiazoles as prospective dual EGFR/BRAF^V600E^ inhibitors

**DOI:** 10.1039/d6ra00207b

**Published:** 2026-03-16

**Authors:** Hesham A. M. Gomaa, Mohamed E. Shaker, Sami I. Alzarea, Eid Alatwi, Fatma A. M. Mohamed, Abdullah Yahya Abdullah Alzahrani, Bandar A. Alyami, Stefan Bräse, Safwat M. Rabea, Bahaa G. M. Youssif

**Affiliations:** a Department of Pharmacology, College of Pharmacy, Jouf University Sakaka 72388 Saudi Arabia hasoliman@ju.edu.sa; b Department of Clinical Laboratory Sciences, College of Applied Medical Sciences at Al-Qurayyat, Jouf University Al-Qurayyat 72388 Saudi Arabia; c Department of Chemistry, Faculty of Science, King Khalid University Abha 61413 Saudi Arabia; d Department of Pharmaceutical Chemistry, College of Pharmacy, Najran University Najran Saudi Arabia; e Institute of Biological and Chemical Systems, IBCS-FMS, Karlsruhe Institute of Technology 76131 Karlsruhe Germany braese@kit.edu; f Medicinal Chemistry Department, Faculty of Pharmacy, Minia University Minia 61519 Egypt; g Pharmaceutical Organic Chemistry Department, Faculty of Pharmacy, Assiut University Assiut 71526 Egypt bgyoussif2@gmail.com +201044353895

## Abstract

A novel series of heteroaryl pyridine-linked 1,2,4-oxadiazole compounds (5, 9, 14, 20a–c, 21a–c, and 22a–c) was developed, synthesized, and investigated as prospective inhibitors of EGFR and BRAF^V600E^. The new compounds were investigated for antiproliferative activity against four human cancer cell lines and for safety in normal mammary epithelial cells (MCF-10A) and a normal human diploid cell line (WI-38). Compounds 20c, 21a–c, and 22b demonstrated significant antiproliferative action, with compounds 20c and 21c exhibiting the highest efficacy. Compounds 20c and 21c exhibited potent inhibition of EGFR, with IC_50_ values of 71 and 64 nM, respectively, surpassing the reference erlotinib (IC50 = 80 nM). Moreover, compounds 20c and 21c exhibited BRAF^V600E^ inhibitory action with IC_50_ values of 49 and 41 nM, respectively, which are somewhat less potent than the reference drug Vemurafenib. Assays for apoptotic markers (Caspases, Bax, Bcl-2, and p53) demonstrated that apoptosis plays a role in the reported antiproliferative effects. Compounds 20c and 21c showed a notable decrease in TNF-α and IL-6 levels compared with dexamethasone, suggesting an immunomodulatory effect. Molecular docking further validated the favorable orientation of 20c and 21c within the ATP-binding pocket of EGFR and BRAF^V600E^. These findings underscore compounds 20c and 21c as innovative dual-target scaffolds with significant promise for anticancer drug development.

## Abstract 2

Vemurafenib triggers resistance *via* EGFR feedback upregulation, compound 21c achieves a “vertical blockade” by simultaneously inhibiting EGFR and mutant BRAF.

## Graphical abstract

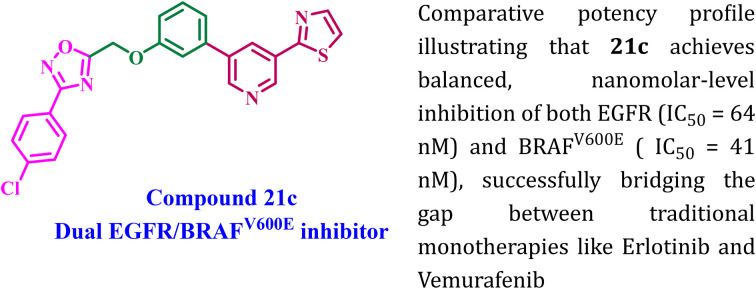

## 1. Introduction

Cancer is a primary cause of morbidity and mortality globally, and its epidemiological profile is becoming increasingly complex.^1^ The American Cancer Society projects that by 2026, over 2.1 million new cancer cases will be diagnosed in the United States. That amounts to over 5800 new cases daily.^2,3^ Breast and colorectal malignancies are two cancer types frequently targeted by novel heterocyclic scaffolds. These malignancies remain a significant public health issue, with an expected 321 910 and 158 850 new cases, respectively.^4^ The cumulative five-year relative survival rate has reached an unprecedented 70%. The rising incidence rates in younger populations and the persistence of metastatic disease highlight the urgent need for innovative treatments that might deliver sustained clinical outcomes.^5,6^

Despite advances in precision medicine and immunotherapy, many modern medications continue to have substantial difficulties. Traditional chemotherapeutic drugs can occasionally lack specificity, resulting in severe systemic toxicities such as myelosuppression and cardiotoxicity, which significantly limit the maximum tolerated dose.^7,8^ Furthermore, the establishment of multidrug resistance (MDR) is a major factor of treatment failure and illness recurrence. These issues call for the development of new chemical frameworks.^9^ These frameworks may improve metabolic stability and cellular permeability, and enable more precise interactions with cancer signaling pathways.^10^ The utilization of multiple drugs in cancer therapy simultaneously targets various pathways; yet, this approach may result in detrimental drug–drug interactions and the development of drug resistance.^11^ As a result, using a single treatment that can influence more than one target is considered a different approach to overcoming the aforementioned limitations.^12,13^ The FDA has authorized several multitarget therapies for cancer, including Dasatinib, a highly effective multitargeted kinase inhibitor.^14^

BRAF^V600E^ is a somatic point mutation that converts thymine to adenine at nucleotide 1799. This modification results in the substitution of valine (V) with glutamic acid (E) at codon 600 inside the kinase domain. This alteration renders the BRAF protein a monomer that is perpetually active and autonomously transmits signals, independent of prior RAS activation. The V600E mutation induces uncontrolled cellular proliferation, evasion of apoptosis, and the emergence of an aggressive oncogenic phenotype by perpetually activating the mitogen-activated protein kinase (MAPK/ERK) pathway. In colorectal cancer (CRC), this mutation serves as a critical molecular marker for a distinct clinical cohort characterized by right-sided primary tumors, poor differentiation, and significantly reduced overall survival rates compared to BRAF wild-type tumors.^15,16^

Although BRAF inhibitors are effective in other V600E-mutant cancers, they are not effective as monotherapy in colorectal cancer due to the disease's rapid adaptation.^17^ Colorectal cancer cells, conversely, react to BRAF inhibition by initiating a feedback loop that elevates the levels of epidermal growth factor receptor (EGFR). This reactivation *via* EGFR circumvents the obstructed BRAF protein and reinstates signaling through the MAPK/ERK pathway, hence sustaining tumor proliferation and viability.^18,19^ To achieve a durable therapeutic blockade, it is essential to utilize both a BRAF inhibitor and an EGFR inhibitor. This combination therapy addresses intrinsic resistance by simultaneously targeting both the mutant driver and its primary escape mechanism.^15^ This results in improved clinical outcomes and establishes a new standard of care for this challenging patient population.

**Fig. 1:**
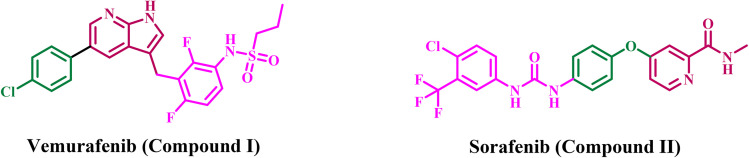
Structure of vemurafenib (I) and sorafenib (II).

Pyridine derivatives constitute a significant category of anticancer treatments, featuring multiple FDA-approved medications (*e.g.*, Sorafenib, Vemurafenib) and a multitude of investigational compounds designed as EGFR and BRAF inhibitors.^20,21^ The pyridine scaffold is a crucial structural element of numerous kinase inhibitors because of its advantageous electronic properties and ability to form robust interactions within the ATP-binding sites of target enzymes.^22^ Sorafenib (Compound I, Fig. 1) is an FDA-approved pyridine derivative used for advanced renal cell carcinoma and hepatocellular carcinoma, functioning as a multikinase inhibitor targeting VEGFR and BRAF.^23^ Vemurafenib (Compound II, Fig. 1), a pyridine derivative, is approved to treat metastatic melanoma by inhibiting the V600E-mutated BRAF kinase specifically.^24^ Researchers are continually developing novel pyridine derivatives, frequently as hybrid compounds, to target both EGFR and BRAF pathways. This dual-targeting strategy seeks to enhance efficacy and diminish resistance mechanisms.^25,26^

**Fig. 2:**
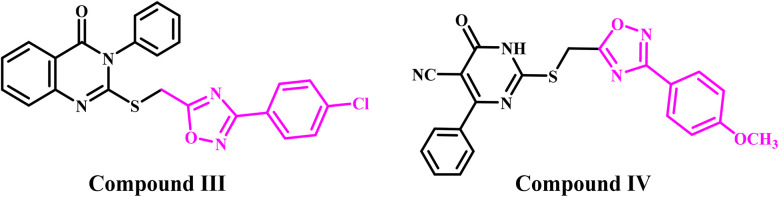
Structures of 1,2,4-oxadiazole-based antiproliferative agents III and IV.

Meanwhile, literature reviews demonstrate that 1,2,4-oxadiazoles hold considerable significance in bioorganic and medicinal chemistry. They are recognized for their diverse pharmacological characteristics.^27,28^ The 1,2,4-oxadiazole exhibits bioisosteric equivalence to ester and amide features. Under unstable conditions (*e.g.*, hydrolysis), 1,2,4-oxadiazole offers a highly effective alternative.^29^ The substantial biological impact of 1,2,4-oxadiazole derivatives on cancer cells is due to multiple mechanisms of action. We recently^30^ displayed a group of 1,2,4-oxadiazole/quinazoline-4-one hybrids designed as antiproliferative agents targeting EGFR/BRAF^V600E^. The results indicated that compound III (Fig. 2) exhibited the highest antiproliferative activity. Compound III demonstrated significant efficacy as an EGFR and BRAF^V600E^ inhibitor, with pronounced apoptotic action.

In a separate publication from our laboratory,^29^ we reported the synthesis of a novel class of dihydropyrimidine-5-carbonitrile/1,2,4-oxadiazole hybrids that serve as dual inhibitors of EGFR and VEGFR-2 and exhibit antioxidant properties. Compound IV (Fig. 2) exhibited the most significant antiproliferative activity, exceeding the efficacy of erlotinib against Panc-1 (pancreatic) and MCF-7 (breast) cancer cell lines. Compound IV had the highest inhibitory action against EGFR and VEGFR-2, with IC_50_ values of 57 nM for EGFR and 21 nM for VEGFR-2. Additionally, compound IV exhibited significant apoptotic activity by upregulating caspases-3, 8, and 9, along with Bax and p53, and downregulating the anti-apoptotic protein Bcl-2.

**Fig. 3:**
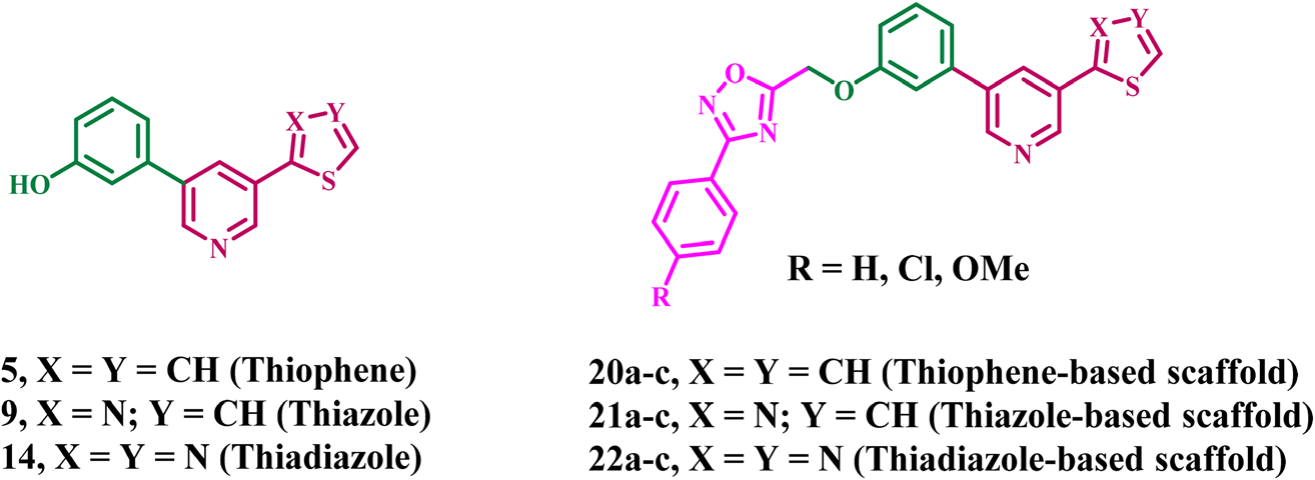
Structures of new compounds 5, 9, 14, 20a–c, 21a–c, and 22a–c.

In light of the previously discussed anticancer attributes of pyridine and 1,2,4-oxadiazole derivatives as EGFR and/or BRAF^V600E^ inhibitors, and to advance our goal of developing novel inhibitors that target both EGFR and BRAF^V600E^,^15,20,31–35^ here, we detail the development, and antiproliferative testing of a handful of novel compounds (20a–c, 21a–c, and 22a–c, Fig. 3) that simultaneously targeting EGFR and BRAF^V600E^.

The molecular structure was developed using a dual-binding approach that included various pharmacophores tailored to the conserved and divergent characteristics of the target kinases. The aryl-pyridine core was selected as the primary hinge-binding motif to facilitate anchoring interactions within the ATP-binding pockets of both EGFR and BRAF^V600E^. A 1,2,4-oxadiazole moiety functions as a rigid linker to ensure optimal spatial configuration. It provides the appropriate configuration to link the hinge and supplementary binding regions. Finally, a substituted phenyl tail was included to enhance lipophilicity and accommodate the configuration of distal hydrophobic areas, particularly the “back pocket” of BRAF, hence augmenting potency and selectivity.

The structural confirmation of the novel compounds was achieved using ^1^H NMR, ^13^C NMR, elemental analysis, and LC-MS for selected typical instances. The new compounds will be assessed for antiproliferative activity using the MTT assay against four different cancer cell lines. The most effective compounds will be evaluated as dual EGFR/BRAF^V600E^ inhibitors. Additionally, several selected compounds will be evaluated for their effects on normal cell lines, and their apoptotic and immunomodulatory capabilities. Ultimately, docking research will be employed to examine the binding interactions of some selected derivatives with the designated receptor sites.

## 2. Experimental

### 2.1. Chemistry

General details: see Appendix A (SI).

The starting materials, 3-aryl-5-(chloromethyl)-1,2,4-oxadiazoles 19a–c, were prepared according to literature methods.^30^

#### 2.1.1. Synthesis of 3-bromo-5-(thiophen-2-yl)pyridine (3)

At room temperature, a stirred solution of 3,5-dibromopyridine 1 (15 g, 63.32 mmol) in 1,4-dioxane (100 mL) was mixed with thiophen-2-yl boronic acid 2 (10.13 g, 79.15 mmol) and Cs_2_CO_3_ (34.04 g, 104.48 mmol). The reaction mixture was degassed for 15 min, after which Pd(PPh_3_)_4_ (5.12 g, 4.43 mmol) was introduced. The reaction mixture was stirred at 100 °C for 2 h in a nitrogen environment. Upon completion of the reaction (monitored using TLC), the reaction mixture was allowed to cool to ambient temperature, after which the solvent was removed under lowered pressure. The residue was dissolved in ethyl acetate and washed with water, followed by brine. The organic layer was dried over sodium sulfate, then evaporated under reduced pressure. The residue was purified by automated normal-phase chromatography, eluting with ethyl acetate/petroleum ether, to give 3-bromo-5-(thiophen-2-yl)pyridine 3 (6 g, 40% yield) as an off-white solid. ^1^H NMR (400 MHz, DMSO-d6): *δ* 8.88 (d, *J* = 2.10 Hz, 1H), 8.62 (d, *J* = 2.10 Hz, 1H), 8.35 (t, *J* = 2.10 Hz, 1H), 7.76–7.70 (m, 2H), 7.22–7.19 (m, 1H); MS (ES + APCI) *m*/*z* 242.0 (M + 2).

#### 2.1.2. Synthesis of 3-(5-(thiophen-2-yl)pyridin-3-yl)phenol (5)

At room temperature, a stirred solution of 3-bromo-5-(thiophen-2-yl)pyridine 3 (6 g, 25 mmol) in 1,4-dioxane (60 mL) and water (6 mL) was treated with (3-hydroxyphenyl)boronic acid 4 (4.14 g, 30 mmol) and Cs_2_CO_3_ (16.28 g, 50 mmol). The reaction mixture was degassed for 15 min before Pd(dppf)Cl_2_ (0.1 g, 0.08 mmol) was introduced. The reaction mixture was agitated at 90 °C for 3 h under a nitrogen atmosphere. Following the completion of the reaction (as monitored by TLC), the reaction mixture was cooled to room temperature before the solvent was evaporated under reduced pressure. The residue was solubilized with ethyl acetate, subsequently washed with water and then brine. The organic layer was dried using sodium sulfate and subsequently evaporated at decreased pressure. The residue underwent purification *via* automated normal-phase chromatography and was eluted with ethyl acetate/petroleum ether, yielding 3-(5-(thiophen-2-yl)pyridin-3-yl)phenol 5 (4 g, 63% yield) as an off-white solid. ^1^H NMR (400 MHz, DMSO-d6): *δ* 9.66 (s, 1H), 8.88 (d, *J* = 2.00 Hz, 1H), 8.74 (d, *J* = 2.00 Hz, 1H), 8.19 (t, *J* = 2.00 Hz, 1H), 7.79–7.78 (m, 1H), 7.70–7.68 (m, 1H), 7.33 (t, *J* = 8.00 Hz, 1H), 7.23–7.20 (m, 2H), 7.15 (t, *J* = 2.00 Hz, 1H), 6.88–6.86 (m, 1H); MS (ES + APCI) *m*/*z* 254.3 (M + 1).

#### 2.1.3. Synthesis of 2-(5-bromopyridin-3-yl)thiazole (8)

At room temperature, a stirred solution of 2-bromothiazole 6 (12 g, 73.17 mmol) in 1,4-dioxane (120 mL) was mixed with (5-bromopyridin-3-yl)boronic acid 7 (18 g, 87.80 mmol) and Cs_2_CO_3_ (35.8 g, 109.8 mmol). The reaction mixture was degassed for 15 minutes then Pd(PPh_3_)_4_ (5.1 g, 4.40 mmol) was added. The reaction mixture was agitated at 100 °C for 5 h in a nitrogen environment. Upon completion of the reaction (detected by TLC), the reaction mixture was cooled to room temperature, and subsequently, the solvent was evaporated under reduced pressure. The residue was purified by automated normal-phase chromatography and eluted with ethyl acetate/petroleum ether to give 2-(5-bromopyridin-3-yl)thiazole 8 (4.5 g, 24% yield) as an off-white solid. ^1^H-NMR (400 MHz, DMSO-d6): *δ* 9.13 (d, *J* = 2.80 Hz, 1H), 8.81 (d, *J* = 3.20 Hz, 1H), 8.53 (t, *J* = 2.80 Hz, 1H), 8.04 (d, *J* = 4.40 Hz, 1H), 7.96 (d, *J* = 4.40 Hz, 1H); MS (ES + APCI) *m*/*z* 243.1(M + 2).

#### 2.1.4. Synthesis of 3-(5-(thiazol-2-yl)pyridin-3-yl)phenol (9)

At room temperature, a stirred solution of 2-(5-bromopyridin-3-yl)thiazole 8 (4.5 g, 18.66 mmol) in 1,4-dioxane (50 mL) and water (5 mL) was mixed with (3-hydroxyphenyl)boronic acid 4 (2.9 g, 20.53 mmol) and K_2_CO_3_ (18.3 g, 56.0 mmol). The reaction mixture was degassed for 15 minutes then Pd(PPh_3_)_4_ (1.1 g, 0.95 mmol) was added. The reaction mixture was agitated at 90 °C for 5 h in a nitrogen environment. Upon completion of the reaction (monitored by TLC), the reaction mixture was cooled to room temperature, and the solvent was then evaporated under reduced pressure. The residue underwent purification *via* automated normal-phase chromatography and was eluted with ethyl acetate/petroleum ether, yielding 3-(5-(thiazol-2-yl)pyridin-3-yl)phenol 9 (2.2 g, 46% yield) as an off-white solid. ^1^H NMR (400 MHz, DMSO-d6): *δ* 9.69 (s, 1H), 9.13 (d, *J* = 2.00 Hz, 1H), 8.92 (d, *J* = 2.00 Hz, 1H), 8.43 (t, *J* = 2.40 Hz, 1H), 8.05 (d, *J* = 3.20 Hz, 1H), 7.94 (d, *J* = 3.20 Hz, 1H), 7.35 (t, *J* = 8.00 Hz, 1H), 7.24 (d, *J* = 8.00 Hz, 1H), 7.16 (t, *J* = 2.00 Hz, 1H), 6.89–6.87 (m, 1H); MS (ES + APCI) *m*/*z* 255.2 (M + 1).

#### 2.1.5. Synthesis of 5-bromonicotinohydrazide (11)

To a stirred solution of methyl 5-bromonicotinate 10 (5 g, 23.14 mmol) in ethanol (50 mL) was added hydrazine hydrate (25 mL, 325 mmol) at room temperature. The reaction mixture was stirred at 60 °C for 15 h. After completion of the reaction (monitored by TLC), the resulting mixture was concentrated to a residue. The residue co-evaporated with toluene to remove the residual water and repeated the toluene co-evaporation process for 3 to 4 times to give 5-bromonicotinohydrazide 2 (5 g, 100% yield) which was used for next step without further purification. ^1^H NMR (400 MHz, DMSO-d6) *δ* 8.94 (d, *J* = 1.80 Hz, 1H), 8.84 (d, *J* = 2.10 Hz, 1H), 8.37 (t, *J* = 2.10 Hz, 1H); MS (ES + APCI) *m*/*z* 216.2.

#### 2.1.6. Synthesis of 5-bromo-*N*′-formylnicotinohydrazide (12)

To a stirred solution of 5-bromonicotinohydrazide 11 (5 g, 23.14 mmol) in formic acid (10 mL) was stirred at room temperature for 16 h under nitrogen atmosphere. After completion of the reaction (monitored by TLC), the reaction mixture was evaporated under reduced pressure. The residue was triturated with MTBE, and the precipitated solid was filtered and dried to give 5-bromo-*N*′-formylnicotinohydrazide 12 (5.5 g, 97% yield) as an off-white solid. ^1^H NMR (400 MHz, CD_3_OD): *δ* 8.99 (d, *J* = 2.00 Hz, 1H), 8.90–8.87 (m, 1H), 8.47 (t, *J* = 2.00 Hz, 1H), 8.19 (d, *J* = 7.20 Hz, 1H); MS (ES + APCI) *m*/*z* 246.0 (M + 2).

#### 2.1.7. Synthesis of 2-(5-bromopyridin-3-yl)-1,3,4-thiadiazole (13)

To a stirred solution of 5-bromo-*N*′-formylnicotinohydrazide 12 (5.5 g, 22.54 mmol) in pyridine (55 mL) was added phosphorus pentasulfide (10.02 g, 22.54 mmol) at room temperature. The reaction mixture was stirred at 115 °C for 16 h. After completion of the reaction (monitored by TLC), the resulting mixture was concentrated to a residue. The residue was quenched with 1.5 N HCl solution and extracted with ethyl acetate. The organic layer was dried over sodium sulfate then evaporated under reduced pressure. The residue was purified by automated normal-phase chromatography and eluted with ethyl acetate/petroleum ether to give 2-(5-bromopyridin-3-yl)-1,3,4-thiadiazole 13 (2.5 g, 44% yield) as yellow solid. ^1^H NMR (400 MHz, DMSO-d6) *δ* 9.76 (s, 1H), 9.20 (d, *J* = 1.50 Hz, 1H), 8.91 (d, *J* = 1.50 Hz, 1H), 8.67 (t, *J* = 1.50 Hz, 1H): MS (ES + APCI) *m*/*z* 244.1 (M + 2).

#### 2.1.8. Synthesis of 3-(5-(1,3,4-thiadiazol-2-yl)pyridin-3-yl)phenol (14)

3-(Hydroxyphenyl)boronic acid 4 (6.70 g, 48.6 mmol) and K_2_CO_3_ (11.19 g, 81 mmol) were added to a stirred solution of 2-(5-bromopyridin-3-yl)-1,3,4-thiadiazole 13 (4.9 g, 20.24 mmol) in 1,4-dioxane (40 mL) and water (10 mL) at room temperature. The reaction mixture was degassed for 15 min, after which Pd(PPh_3_)_4_ (4.68 g, 4.05 mmol) was introduced. The reaction mixture was agitated at 80 °C for 4 h in a nitrogen environment. Upon completion of the reaction, as monitored by TLC, the reaction mixture was cooled to ambient temperature, and subsequently, the solvent was evaporated under reduced pressure. The residue was quenched with water and extracted using ethyl acetate; the mixed organic layers were washed with water and brine, then concentrated to provide a residue. The residue underwent purification *via* automated normal-phase chromatography and was eluted with ethyl acetate/petroleum ether, yielding 3-(5-(1,3,4-thiadiazol-2-yl)pyridin-3-yl)phenol 14 (3.38 g, 65% yield) as a pale yellow solid. ^1^H NMR (400 MHz, DMSO-d_6_): *δ* 9.76 (s, 1H), 9.70 (s, 1H), 9.18 (d, *J* = 2.00 Hz, 1H), 9.01 (d, *J* = 2.40 Hz, 1H), 8.52 (t, *J* = 2.40 Hz, 1H), 7.35 (t, *J* = 8.00 Hz, 1H), 7.27–7.25 (m, 1H), 7.19 (t, *J* = 1.60 Hz, 1H), 6.91–6.88 (m, 1H); MS (ES + APCI) *m*/*z* 256.3 (M + 1).

#### 2.1.9. General procedures for the synthesis of new compounds (20a–c, 21a–c, and 22a–c)

A stirred solution of phenolic scaffolds 5, 9, and 14 (0.60 mmol, 1 eq) in DMF (6 mL) was mixed with of K_2_CO_3_ (0.72 mmol, 1.2 eq., 0.10 g), followed by stirring for 1 h at ambient temperature. Subsequently, oxadiazoles 19a–c (0.60 mmol, 1 eq.) and KI (0.90 mmol, 1.5 eq., 0.15 g) were included into the reaction mixture, which was stirred for 24 h. Upon completion of the reaction (verified by TLC), the reaction mixture was added to crushed ice while stirring. The precipitate was filtered, washed repeatedly with water, dried at 60 °C, and recrystallized from ethanol to yield pure 20a–c, 21a–c, and 22a–c.

##### 2.1.9.1. 3-Phenyl-5-((3-(5-(thiophen-2-yl)pyridin-3-yl)phenoxy)methyl)-1,2,4-oxadiazole (20a)

Yield: 0.17 g (69%), brown solid, m.p: 157–159 °C. ^1^H NMR (400 MHz, *δ* ppm CDCl_3_): 8.89 (d, *J* = 2.1 Hz, 1H, Ar–H), 8.75 (d, *J* = 2.1 Hz, 1H, Ar–H), 8.13 (dd, *J* = 7.8, 1.5 Hz, 1H, Ar–H), 8.02 (t, *J* = 2.1 Hz, 1H, Ar–H), 7.52–7.47 (m, 4H, Ar–H), 7.44 (dd, *J* = 3.5, 1.2 Hz, 1H, Ar–H), 7.41 (dd, *J* = 5.2, 1.2 Hz, 1H, Ar–H), 7.34–7.30 (m, 2H, Ar–H), 7.28 (s, 1H, Ar–H), 7.17 (dd, *J* = 5.2, 3.5 Hz, 1H, Ar–H), 7.11 (ddd, *J* = 8.3, 2.4, 1.2 Hz, 1H, Ar–H), 5.46 (s, 2H, O–CH_2_); ^13^C NMR (100 MHz, *δ* ppm CDCl_3_): 174.54, 168.61, 158.16, 146.92, 146.04, 140.13, 139.39, 138.34, 137.73, 136.14, 131.52, 130.58, 130.42, 128.35, 126.25, 126.09, 124.76, 124.59, 121.32, 114.38, 114.28, 61.29; Anal. Calc. (%) For C_24_H_17_N_3_O_2_S: C, 70.06; H, 4.16; N, 10.21; S, 7.79. Found: C, 70.15; H, 4.20; N, 10.32; S, 7.71.

##### 2.1.9.2. 3-(4-Chlorophenyl-5-((3-(5-(thiophen-2-yl)pyridin-3-yl)phenoxy)methyl)-1,2,4-oxadiazole (20b)

Yield: 0.20 g (75%), brown solid, m.p: 160–162 °C. ^1^H NMR (400 MHz, *δ* ppm CDCl_3_): 8.89 (d, *J* = 2.2 Hz, 1H, Ar–H), 8.75 (d, *J* = 2.2 Hz, 1H, Ar–H), 8.07 (d, *J* = 8.7 Hz, 2H, Ar–H 4-Cl C_6_H_5_), 8.02 (t, *J* = 2.2 Hz, 1H, Ar–H), 7.49 (d, *J* = 8.7 Hz, 2H, Ar–H 4-Cl C_6_H_5_), 7.44 (dd, *J* = 3.6, 1.2 Hz, 1H, Ar–H), 7.41 (dd, *J* = 5.1, 1.1 Hz, 1H, Ar–H), 7.35–7.32 (m, 2H, Ar–H), 7.28 (s, 1H, Ar–H), 7.17 (dd, *J* = 5.1, 3.5 Hz, 1H, Ar–H), 7.11 (ddd, *J* = 8.3, 2.4, 1.1 Hz, 1H, Ar–H), 5.46 (s, 2H, O–CH_2_); ^13^C NMR (100 MHz, *δ* ppm CDCl_3_): 174.83, 167.89, 158.12, 146.94, 146.04, 140.14, 139.40, 137.72, 136.15, 131.47, 130.54, 130.44, 129.31, 128.89, 128.39, 126.32, 124.76, 124.56, 121.39, 114.34, 114.25, 61.20; anal. calc. (%) For C_24_H_16_ClN_3_O_2_S: C, 64.64; H, 3.62; N, 9.42; S, 7.19. Found: C, 64.72; H, 3.67; N, 9.40; S, 7.10.

##### 2.1.9.3. 3-(4-Methoxyphenyl-5-((3-(5-(thiophen-2-yl)pyridin-3-yl)phenoxy)methyl)-1,2,4-oxadiazole (20c)

Yield: 0.18 g (68%), brown solid, m.p: 165–167 °C. ^1^H NMR (400 MHz, *δ* ppm DMSO-*d*_6_): 9.32 (s, 1H, Ar–H), 8.98 (s, 1H, Ar–H), 8.43 (s, 1H, Ar–H), 7.98 (d, *J* = 8.8 Hz, 2H, Ar–H), 7.68 (dd, *J* = 6.0, 1.5 Hz, 1H, Ar–H), 7.61 (dd, *J* = 6.0, 1.5 Hz, 1H, Ar–H), 7.33 (t, *J* = 7.8 Hz, 1H, Ar–H), 7.26 (d, *J* = 7.8 Hz, 1H, Ar–H), 7.20 (d, *J* = 5.5 Hz, 1H, Ar–H), 7.17 (d, *J* = 8.8 Hz, 2H, Ar–H), 7.10 (s, 1H, Ar–H), 6.82 (d, *J* = 7.8 Hz, 1H, Ar–H), 5.61 (s, 2H, O–CH_2_), 3.81 (s, 3H, O–CH_3_); ^13^C NMR (100 MHz, *δ* ppm DMSO-*d*_6_): 174.80, 167.86, 158.10, 146.95, 146.03, 140.15, 139.42, 137.75, 136.14, 131.48, 130.53, 130.44, 128.67, 128.38, 126.32, 124.74, 124.53, 121.36, 114.30, 114.21, 113.34, 61.19, 55.39; anal. calc. (%) For C_25_H_19_N_3_O_3_S: C, 68.01; H, 4.34; N, 9.52; S, 7.26. Found: C, 68.13; H, 4.30; N, 9.60; S, 7.18.

##### 2.1.9.4. 3-Phenyl-5-((3-(5-(thiazol-2-yl)pyridin-3-yl)phenoxy)methyl)-1,2,4-oxadiazole (21a)

Yield: 0.18 g (73%), Yellow solid, m.p: 160–162 °C. ^1^H NMR (400 MHz, *δ* ppm CDCl_3_): 9.16 (d, *J* = 2.0 Hz, 1H, Ar–H), 8.97 (d, *J* = 2.0 Hz, 1H, Ar–H), 8.55 (t, *J* = 2.0 Hz, 1H, Ar–H), 8.12 (dd, *J* = 7.7, 1.5 Hz, 1H, Ar–H), 7.98 (d, *J* = 3.3 Hz, 1H, Ar–H), 7.52–7.49 (m, 4H, Ar–H), 7.46 (d, *J* = 3.3 Hz, 1H, Ar–H), 7.35 (dd, *J* = 7.4, 1.2 Hz, 2H, Ar–H), 7.27 (s, 1H, Ar–H), 7.16–7.12 (m, 1H, Ar–H), 5.46 (s, 2H, O–CH_2_); ^13^C NMR (100 MHz, *δ* ppm CDCl_3_): 174.41, 168.60, 164.99, 158.22, 156.58, 151.70, 150.54, 147.85, 138.31, 136.58, 133.29, 131.50, 130.70, 128.94, 127.55, 126.23, 126.05, 121.31, 114.76, 114.21, 61.20; anal. calc. (%) For C_23_H_16_N_4_O_2_S: C, 66.98; H, 3.91; N, 13.58; S, 7.77. Found: C, 66.72; H, 3.98; N, 13.49; S, 7.82.

##### 2.1.9.5. 3-(4-Chlorophenyl-5-((3-(5-(thiazol-2-yl)pyridin-3-yl)phenoxy)methyl)-1,2,4-oxadiazole (21b)

Yield: 0.19 g (72%), yellow solid, m.p: 168–170 °C. ^1^H NMR (400 MHz, *δ* ppm CDCl_3_): 9.18 (d, *J* = 2.2 Hz, 1H, Ar–H), 8.90 (d, *J* = 2.2 Hz, 1H, Ar–H), 8.46 (t, *J* = 2.2 Hz, 1H, Ar–H), 8.08 (d, *J* = 8.6 Hz, 2H, Ar–H 4-Cl C_6_H_5_), 7.98 (d, *J* = 3.2 Hz, 1H, Ar–H), 7.49 (d, *J* = 8.6 Hz, 2H, Ar–H 4-Cl C_6_H_5_), 7.47 (d, *J* = 3.2 Hz, 1H, Ar–H), 7.37 (dd, *J* = 4.0, 1.8 Hz, 2H, Ar–H), 7.29 (s, 1H, Ar–H), 7.12 (dd, *J* = 7.9, 3.1 Hz, 1H, Ar–H), 5.46 (s, 2H, O–CH_2_); ^13^C NMR (100 MHz, *δ* ppm CDCl_3_): 174.78, 167.89, 164.65, 158.16, 149.21, 146.74, 144.22, 138.92, 137.71, 136.30, 131.95, 130.59, 129.65, 129.30, 128.90, 124.78, 121.40, 119.88, 114.57, 114.23, 61.20; Anal. Calc. (%) For C_23_H_15_ClN_4_O_2_S: C, 61.81; H, 3.38; N, 12.54; S, 7.17. Found: C, 61.78; H, 3.32; N, 12.46; S, 7.30.

##### 2.1.9.6. 3-(4-Methoxyphenyl-5-((3-(5-(thiazol-2-yl)pyridin-3-yl)phenoxy)methyl)-1,2,4-oxadiazole (21c)

Yield: 0.19 g (73%), yellow solid, m.p: 176–178 °C. ^1^H NMR (400 MHz, *δ* ppm DMSO-*d*_6_): 9.32 (s, 1H, Ar–H), 8.98 (s, 1H, Ar–H), 8.43 (s, 1H, Ar–H), 7.98 (d, *J* = 8.6 Hz, 2H, Ar–H 4-OCH_3_ C_6_H_4_), 7.59 (d, *J* = 5.4 Hz, 1H, Ar–H), 7.44 (d, *J* = 5.4 Hz, 1H, Ar–H), 7.33 (t, *J* = 7.7 Hz, 1H, Ar–H), 7.26 (d, *J* = 7.8 Hz, 1H, Ar–H), 7.17 (d, *J* = 8.6 Hz, 2H, Ar–H 4-OCH_3_ C_6_H_4_), 7.10 (s, 1H, Ar–H), 6.82 (d, *J* = 7.8 Hz, 1H, Ar–H), 5.61 (s, 2H, O–CH_2_), 3.81 (s, 3H, O–CH_3_); ^13^C NMR (100 MHz, *δ* ppm DMSO-*d*_6_): 174.76, 167.88, 164.63, 158.16, 149.20, 146.75, 144.23, 138.91, 137.73, 136.32, 131.90, 130.57, 129.66, 128.69, 124.80, 121.43, 119.86, 114.84, 114.54, 114.20, 113.34, 61.20, 55.38; anal. calc. (%) For C_24_H_18_N_4_O_3_S: C, 65.15; H, 4.10; N, 12.66; S, 7.25. Found: C, 65.18; H, 4.03; N, 12.75; S, 7.39.

##### 2.1.9.7. 5-((3-(5-(1,3,4-Thiadiazol-2yl)pyridin-3-yl)phenoxy)methyl)-3-phenyl-1,2,4-oxadiazole (22a)

Yield: 0.17 g (70%), orange solid, m.p: 165–167 °C. ^1^H NMR (400 MHz, *δ* ppm CDCl_3_): 9.23 (s, 1H, Ar–H), 9.17 (d, *J* = 2.2 Hz, 1H, Ar–H), 8.98 (d, *J* = 2.2 Hz, 1H, Ar–H), 8.56 (t, *J* = 2.2 Hz, 1H, Ar–H), 8.13 (dd, *J* = 7.8, 1.6 Hz, 1H, Ar–H), 7.57–7.46 (m, 4H, Ar–H), 7.36 (dd, *J* = 7.5, 1.3 Hz, 2H, Ar–H), 7.28 (s, 1H, Ar–H), 7.17–7.13 (m, 1H, Ar–H), 5.47 (s, 2H, O–CH_2_); ^13^C NMR (100 MHz, *δ* ppm CDCl_3_): 174.49, 168.66, 165.03, 158.26, 151.76, 150.56, 147.88, 138.34, 136.62, 133.31, 131.52, 130.72, 128.96, 127.58, 126.25, 126.09, 121.33, 114.81, 114.29, 61.23; anal. calc. (%) For C_22_H_15_N_5_O_2_S: C, 63.91; H, 3.66; N, 16.94; S, 7.75. Found: C, 64.03; H, 3.71; N, 16.99; S, 7.68.

##### 2.1.9.8. 5-((3-(5-(1,3,4-Thiadiazol-2-yl)pyridin-3-yl)phenoxy)methyl)-3-(4-chlorophenyl)-1,2,4-oxadiazole (22b)

Yield: 0.20 g (75%), orange solid, m.p: 173–175 °C. ^1^H NMR (400 MHz, *δ* ppm CDCl_3_): 9.24 (s, 1H, Ar–H), 9.17 (d, *J* = 2.1 Hz, 1H, Ar–H), 8.98 (d, *J* = 2.1 Hz, 1H, Ar–H), 8.58 (t, *J* = 2.1 Hz, 1H, Ar–H), 8.08 (d, *J* = 8.8 Hz, 2H, Ar–H 4-Cl C_6_H_5_), 7.50 (d, *J* = 8.8 Hz, 2H, Ar–H 4-Cl C_6_H_5_), 7.39–7.36 (m, 2H, Ar–H), 7.28 (s, 1H, Ar–H), 7.14 (ddd, *J* = 8.2, 2.4, 1.0 Hz, 1H, Ar–H), 5.47 (s, 2H, O–CH_2_); ^13^C NMR (100 MHz, *δ* ppm CDCl_3_): 174.71, 167.91, 164.99, 158.22, 151.74, 150.56, 147.94, 138.38, 137.74, 136.60, 133.26, 130.74, 129.32, 128.90, 126.12, 124.75, 121.38, 114.83, 114.25, 61.18; anal. calc. (%) For C_22_H_14_ClN_5_O_2_S: C, 59.00; H, 3.15; N, 15.64; S, 7.16. Found: C, 59.08; H, 3.24; N, 15.57; S, 7.10.

##### 2.1.9.9. 5-((3-(5-(1,3,4-Thiadiazol-2-yl)pyridin-3-yl)phenoxy)methyl)-3-(4-methoxyphenyl)-1,2,4-oxadiazole (22c)

Yield: 0.19 g (72%), orange solid, m.p: 182–184 °C. ^1^H NMR (400 MHz, *δ* ppm DMSO-*d*_6_): 9.73 (s, 1H, Ar–H), 9.32 (s, 1H, Ar–H), 8.98 (s, 1H, Ar–H), 8.43 (s, 1H, Ar–H), 7.98 (d, *J* = 8.5 Hz, 2H, Ar–H 4-OCH_3_ C_6_H_4_), 7.33 (t, *J* = 7.7 Hz, 1H, Ar–H), 7.26 (d, *J* = 7.8 Hz, 1H, Ar–H), 7.17 (d, *J* = 8.5 Hz, 2H, Ar–H 4-OCH_3_ C_6_H_4_), 7.10 (s, 1H, Ar–H), 6.82 (d, *J* = 7.9 Hz, 1H, Ar–H), 5.61 (s, 2H, O–CH_2_), 3.81 (s, 3H, O–CH_3_); ^13^C NMR (100 MHz, *δ* ppm DMSO-*d*_6_): 174.72, 167.92, 165.00, 158.73, 155.41, 150.30, 147.46, 137.74, 136.74, 133.44, 130.83, 128.68, 126.36, 121.37, 118.31, 116.31, 114.84, 114.46, 113.35, 61.18, 55.38; anal. calc. (%) For C_23_H_17_N_5_O_3_S: C, 62.29; H, 3.86; N, 15.79; S, 7.23. Found: C, 62.35; H, 3.77; N, 15.85; S, 7.18.

### 2.2. Biology

#### 2.2.1. Cell viability assay

Using the human mammary gland epithelial cell line (MCF-10A), derivatives 5, 9, 14, 20a–c, 21a–c, and 22a–c were tested for their effects on cell viability. After a 4-days incubation with MCF-10A cells, the MTT test was used to determine the viability of 5, 9, 14, 20a–c, 21a–c, and 22a–c.^36^ Check Appendix A for additional information.

#### 2.2.2. Antiproliferative assay

The antiproliferative effectiveness of compounds 5, 9, 14, 20a–c, 21a–c, and 22a–c was assessed using the MTT assay on four human cancer cell lines: HT-29 (colon cancer), Panc-1 (pancreatic cancer), A-549 (lung cancer), and MCF-7 (breast cancer).^37,38^ All cell lines were purchased from ATCC (American Type Cell Culture) *via* the Holding Company for Biological Products and Vaccines (VACSERA) in Cairo, Egypt. Erlotinib functioned as a reference. The IC_50_ values for novel compounds were determined by dose–response assays. Each concentration was evaluated in triplicate across at least two distinct experiments, yielding the reported values. Appendix A (SI) contains experimental details.

#### 2.2.3. EGFR inhibitory assay

The EGFR-TK assay^39^ has been employed to evaluate the inhibitory activity of the most effective derivatives, 20c, 21a–c, and 22b, against EGFR, with erlotinib as the reference compound. Appendix A (SI) contains experimental details.

#### 2.2.4. BRAF^V600E^ inhibitory assay

The kinase assay^40^ was employed to assess the inhibitory potential of compounds 20c, 21a–c, and 22b against BRAF^V600E^. Vemurafenib served as the reference agent. Review Appendix A for additional information.

#### 2.2.5. TNF-α and IL-6 inhibitory assays

The impact of compounds 20c and 21c on the expression of TNF-α and IL-6 was assessed using the qRT-PCR assay.^41^ Refer to Appendix A for additional information.

#### 2.2.6. Apoptotic markers assay

Compounds 20c and 21c were evaluated for their ability to trigger apoptosis in A-549 lung cancer cells by analyzing the expression of key apoptotic markers, specifically Caspases-3, -8, -9, Bcl-2, p53, and Bax.^42^ Refer to Appendix A (Supplementary File) for comprehensive details of all experimental trials.

#### 2.2.7. Docking studies

Molecular modeling investigations were conducted using Autodock Vina software. The Autodock Vina tools was utilized to import the structures of compounds 20c and 21c for this investigation.^43,44^ Refer to Appendix A for experimental specifics.

## 3. Results and discussion

### 3.1. Chemistry

**Scheme 1:**
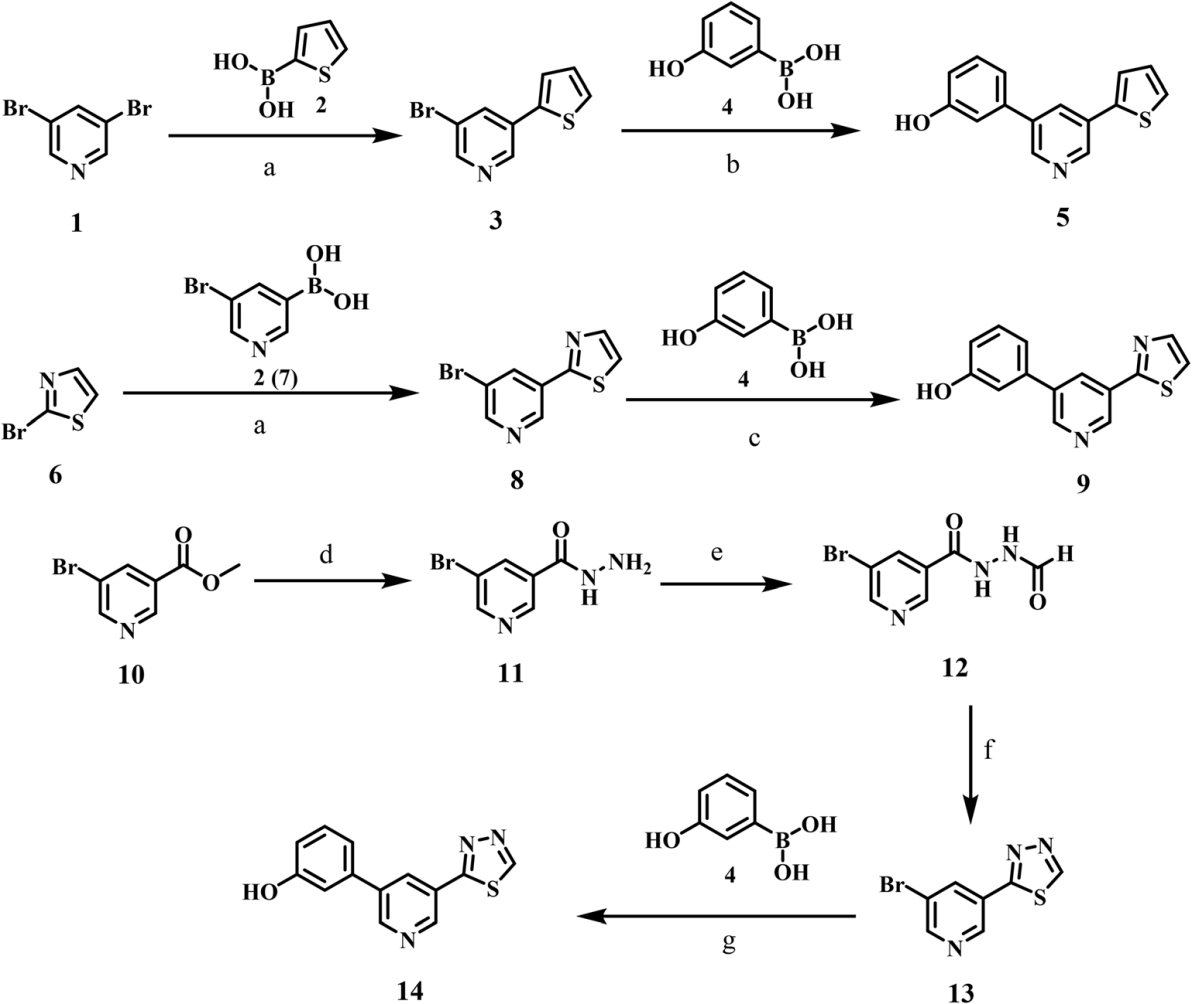
Synthesis of the key scaffolds 5, 9, and 14. Reagents and reaction conditions: (a) Pd(PPh_3_)_4_, Cs_2_CO_3_, 1,4-dioxane, 100 °C, 2 h, 40%; (b) Pd(dppf)Cl_2_, Cs_2_CO_3_, 1,4-dioxane, 90 °C, 3 h, 63%; (c) Pd(PPh_3_)_4_, K_2_CO_3_, 1,4-dioxane/H_2_O, 90 °C, 5 h, 46%; d) NH_2_NH_2_·H_2_O, EtOH, 60 °C, 15 h, 100%; e) HCOOH, RT, 16 h, 97%; (f) pyridine, P_2_S_5_, 115 °C, 16 h, 44%; (g) Pd(PPh_3_)_4_, K_2_CO_3_, 1,4-dioxane, 80 °C, 4 h, 65%.

A convergent method for synthesising intermediate 5 (thiophene–pyridine–phenol) was achieved *via* two successive Suzuki–Miyaura cross-couplings, utilising carefully selected catalyst/ligand complexes to control regioselectivity and functional-group tolerance (Scheme 1). Starting with 3,5-dibromopyridine 1, we decided to insert the thiophene ring *via* coupling with 2-thienylboronic acid 2. To suppress the formation of the undesired symmetrically disubstituted (bis-coupled) analogue, several experimental conditions were tried using different palladium catalysts and ligand sets. Under these optimized conditions, tetrakis(triphenylphosphine)palladium (Pd(PPh_3_)_4_ and cesium carbonate (Cs_2_CO_3_) in 1,4-dioxane at 100 °C, we obtain 3-bromo-5-(thiophen-2-yl)pyridine 3 in 40% yield and recover the unreacted dibromopyridine without observing the formation of the bis-adduct byproduct. It is worth noting that an increase in the reaction temperature to more than 120 °C and/or employing two equivalents of the starting 2-thienylboronic 2 led to the formation of the bis-coupling product. The structure of compound 3 was confirmed using the spectral (^1^H NMR and MS) data. The ^1^H NMR spectrum exhibited three new aromatic signals at 7.76–7.70 and 7.22–7.19 ppm assigned to the protons of the thiophene ring.

In the next step, we employed [1,1′-bis(diphenylphosphino)ferrocene]palladium(ii) dichloride (Pd(dppf)Cl_2_) as a catalyst in presence of Cs_2_CO_3_ as a base for the cross-coupling of intermediate 3 with (3-hydroxyphenyl)boronic acid 4. The reaction proceeded in a 1,4-dioxane/H_2_O system at 90 °C, yielding the target scaffold 5 at 63% efficiency. When the reaction was conducted under the same conditions but without the aqueous component, similar product was obtained in slightly lower yield. The bidentate ligand 1,1′-bis(diphenylphosphino)ferrocene (dppf), together with the dioxane/aqueous medium, proved optimal for *trans*-metalation from a phenylboronic acid bearing a free phenolic group. The ^1^H NMR spectrum of compound 5 displayed four new signals in the aromatic region at *δ* 7.33, 7.23–7.20, 7.15, and 6.88–6.86 ppm, corresponding to the protons of the phenyl ring, along with a phenolic OH signal at *δ* 9.66 ppm.

Key intermediate 9 (thiazole–pyridine–phenol) was obtained in the same fashion using two Suzuki–Miyaura cross-couplings designed to retain chemo-selectivity and functional-group tolerance (Scheme 1). In the first step, 2-bromothiazole 6 was coupled with 5-bromopyridine-3-boronic acid 7 under Pd(PPh_3_)_4_/Cs_2_CO_3_ in 1,4-dioxane at 100 °C, furnishing 2-(5-bromopyridin-3-yl)thiazole 8 in 24% yield. It should be noted that the use of an electron-rich phosphine catalyst with a non-nucleophilic carbonate base enabled the *trans*-metalation from the pyridinyl boronic acid while leaving the C–Br bond on the pyridine ring intact for subsequent derivatization. Next, compound 8 was coupled with (3-hydroxyphenyl)boronic acid 4 using Pd(PPh_3_)_4_ and K_2_CO_3_ in a mixed 1,4-dioxane/H_2_O medium at 90 °C to furnish the target scaffold, 3-(5-(thiazol-2-yl)pyridin-3-yl)phenol 9, in 46% yield. The ^1^H NMR spectrum of compound 9 displayed four new signals in the aromatic region at *δ* 7.35, 7.24, 7.16, and 6.89–6.87 ppm, corresponding to the protons of the phenyl ring, along with a phenolic OH signal at *δ* 9.69 ppm.

Finally, Scheme 1 outlines the synthetic route for key intermediate 14 (thiadiazole–pyridine–phenol). The route began with the hydrazinolysis of methyl 5-bromonicotinate 10 with hydrazine monohydrate in ethanol at 60 °C, yielding hydrazide 11 in quantitative yield. Next, formylation of hydrazide 11 with formic acid gave *N*-formyl hydrazide intermediate 12. The ^1^H NMR spectrum of compound 12 exhibited a signal at *δ* 8.19 ppm due to the CH proton of the formyl group. Subsequent cyclization of *N*-formyl hydrazide 12 using phosphorus pentasulfide (P_2_S_5_) in pyridine resulted in the formation of a 1,3,4-thiadiazole derivative 13. In this reaction, phosphorus pentasulfide functions as both a thionating and dehydrating agent, converting the carbonyl oxygen to sulfur and facilitating the subsequent intramolecular cyclization. The ^1^H NMR spectrum confirmed the formation of thiadiazole derivatives 13. The formyl CH protons were not detected. Instead, the C–H peak of the thiadiazole ring was observed in the aromatic region at *δ* 9.76 ppm. The key intermediate 14 was synthesized *via* a Suzuki–Miyaura cross-coupling reaction of compound 13 with the corresponding (3-hydroxyphenyl)boronic acid 4, employing Pd(PPh3)4 and K_2_CO_3_ as the catalyst in 1,4-dioxane at 80 °C. The ^1^H NMR spectrum of compound 14 displayed four new signals in the aromatic region at *δ* 7.35, 7.27–7.25, 7.19, and 6.91–6.88 ppm, corresponding to the protons of the phenyl ring, along with a phenolic OH signal at *δ* 9.76 ppm.

**Scheme 2:**
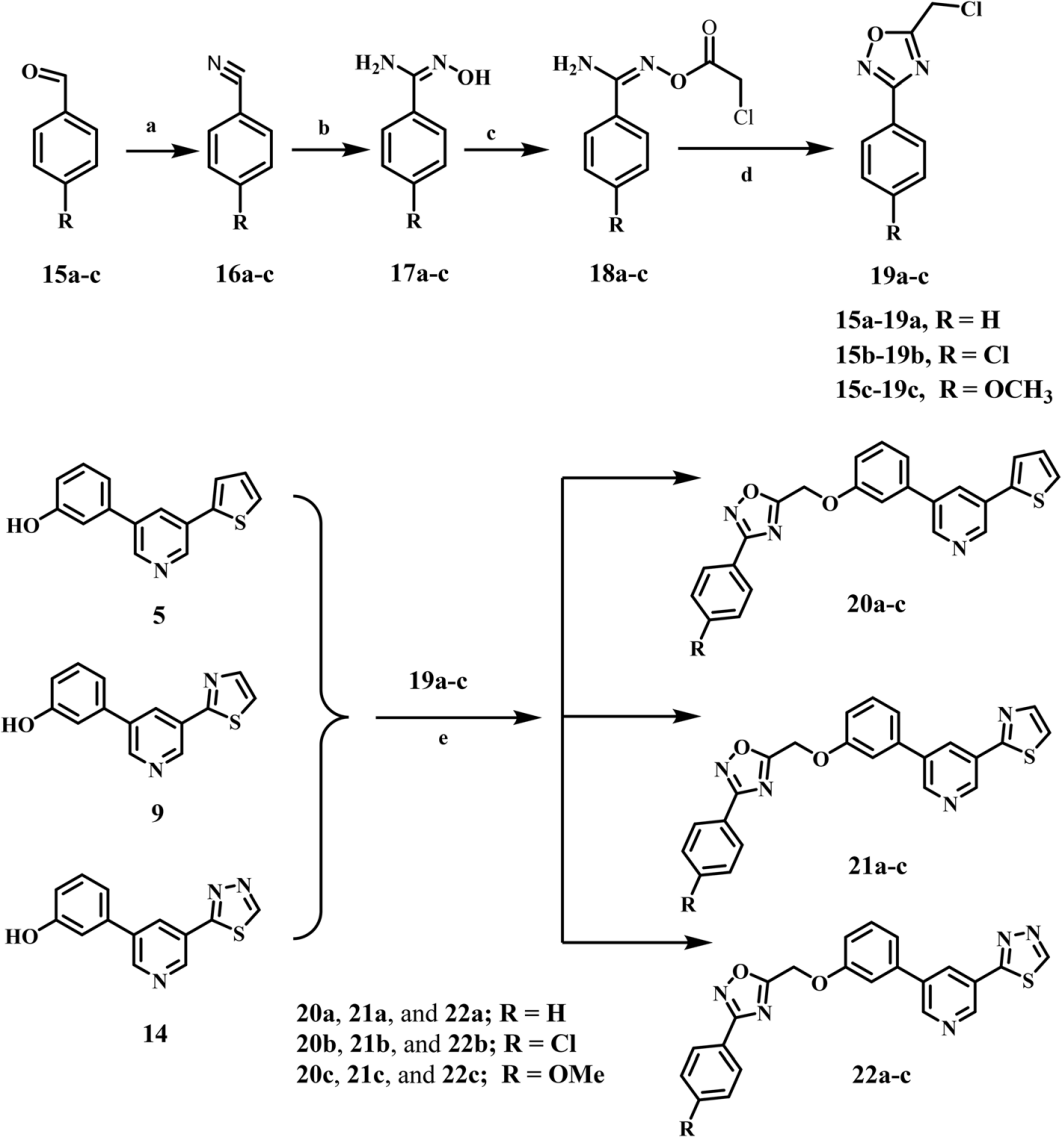
Synthesis of target compounds 20a–c, 21a–c, and 22a–c. Reagents and conditions: (a) ammonia (28%), I_2_, THF, stirring 1 h; (b) NH_2_OH·HCl, Na_2_CO_3_, THF, reflux 18 h; 50–60%, over two steps (c) chloroacetyl chloride, K_2_CO_3_, acetone, RT 24 h; 78% (d) toluene, reflux 10 h; 81% (e) K_2_CO_3_, KI, DMF, stirring 24 h.

Comparatively, amidoxime intermediates 17a–c, were synthesized in two steps with yields ranging from 50 to 60%. The first stage involves reacting the various aldehydes (15a–c) with 28% liquid ammonia and iodine in THF for two to three hours. This produces the aryl nitriles 16a–c in 76–80% yields.^45^ The second stage in the synthesis of 17a–c involved refluxing 16a–c in methanol with hydroxylamine chloride and sodium carbonate for 12 to 18 h. Benzimidamides 18a–c were synthesized by reacting 17a–c with chloroacetyl chloride in anhydrous acetone. The compounds were then cyclized by refluxing in toluene, resulting in the formation of 3-aryl-5-(chloromethyl)-1,2,4-oxadiazoles 19a–c as a yellow oil,^30^Scheme 2. Oxadiazoles 19a–c were purified by column chromatography utilizing a hexane: ethyl acetate (9 : 1) eluent.

The novel compounds 20a–c, 21a–c, and 22a–c were synthesized by reacting intermediates 5, 9, and 14 with oxadiazoles 19a–c in DMF, using K_2_CO_3_ and KI as catalysts, as depicted in Scheme 2. The reaction mixture was stirred overnight and then poured onto crushed ice. The formed precipitate was recrystallized from ethanol, yielding pure 20a–c, 21a–c, and 22a–c. The structures of 20a–c, 21a–c, and 22a–c were validated through ^1^H NMR, ^13^C NMR, and elemental microanalysis. The ^1^H NMR spectrum of compound 22c exhibited two distinct singlet signals: one at *δ* 5.61 ppm, attributed to two protons of the O–CH_2_ group, and another at *δ* 3.81 ppm, corresponding to three protons of the *p*-methoxy group. The spectrum additionally exhibited two doublets at 7.98 (d, *J* = 8.5 Hz, 2H) and 7.17 (d, *J* = 8.5 Hz, 2H) corresponding to a *p*-disubstituted benzene ring. The ^13^C NMR spectrum of 22c exhibited two signals at *δ* 61.18 and 55.38 ppm, corresponding to OCH_2_ and OCH_3_, respectively. Furthermore, all aromatic signals manifested as anticipated.

### 3.2. Biology

#### 3.2.1. Cell viability assay

The impact of new compounds 5, 9, 14, 20a–c, 21a–c, and 22a–c on the viability of the human mammary gland epithelial cell line (MCF-10A) was assessed to evaluate the safety of the synthesised compounds.^36,46^ The MTT test was utilized to evaluate the cell viability of the novel compounds following a four-day incubation with MCF-10A cells. Table 1 indicates that none of the tested compounds exhibited cytotoxicity in normal cells, with all maintaining cell viability above 88% at 50 µM.

**Table 1:** Cell viability% and IC_50_ values of 5, 9, 14, 20a–c, 21a–c, and 22a–c^a^

Comp.	Cell viability%	Antiproliferative activity IC_50_ ± SEM (nM)				
		A-549	MCF-7	Panc-1	HT-29	Average (mean IC_50_)
5	89	64 ± 5	69 ± 5	67 ± 5	67 ± 5	67
9	90	61 ± 5	66 ± 5	64 ± 5	63 ± 5	64
14	91	69 ± 5	74 ± 5	72 ± 6	71 ± 6	72
20a	91	49 ± 4	55 ± 5	53 ± 5	50 ± 4	52
20b	90	45 ± 3	49 ± 3	47 ± 3	46 ± 3	47
20c	92	23 ± 1	26 ± 1	24 ± 1	23 ± 1	24
21a	89	31 ± 2	35 ± 2	34 ± 2	32 ± 2	33
21b	90	27 ± 1	31 ± 2	29 ± 1	28 ± 1	29
21c	91	20 ± 1	23 ± 1	21 ± 1	21 ± 1	21
22a	91	41 ± 3	47 ± 3	45 ± 3	43 ± 3	44
22b	90	36 ± 2	39 ± 2	38 ± 2	36 ± 2	38
22c	92	53 ± 5	58 ± 5	56 ± 5	56 ± 5	56
Erlotinib	ND	30 ± 3	40 ± 3	30 ± 3	30 ± 3	33

a ND: not determined.

#### 3.2.2. Antiproliferative assay

The MTT test^37,38^ was employed to evaluate the antiproliferative effects of 5, 9, 14, 20a–c, 21a–c, and 22a–c on four human cancer cell lines: HT-29 (colon cancer), Panc-1 (pancreatic cancer), A-549 (lung cancer), and MCF-7 (breast cancer). Erlotinib functioned as a reference. Table 1 displays the median inhibitory concentration (IC_50_) and mean IC_50_ (average) values for each compound assessed across the four cancer cell lines.

Generally, compounds 5, 9, 14, 20a–c, 21a–c, and 22a–c exhibited significant antiproliferative activity, with mean IC_50_ values ranging from 21 to 72 nM across the four cancer cell lines, compared with the reference Erlotinib, which had a mean IC_50_ of 33 nM. Furthermore, all evaluated compounds demonstrate greater affinity for the lung (A-549) and colon (HT-29) cancer cell lines than for the other cell lines examined. Compounds 20c, 21a–c, and 22b exhibited the most significant antiproliferative activity, with mean IC_50_ values ranging from 21 to 38 nM. Compounds 20c, 21b, and 21c exhibited superior efficacy than erlotinib against the A-549 lung cancer cell line, with IC_50_ values of 23, 27, and 20 nM, respectively, compared to 30 nM for Erlotinib. Furthermore, derivatives 20c, 21b, and 21c demonstrated enhanced activity relative to erlotinib against the HT-29 (colon) cancer cell line. Their IC_50_ values were 23, 28, and 21 nM, respectively, whereas erlotinib demonstrated an IC_50_ value of 30 nM.

Compound 21c (Thiazole scaffold, R = 4-OMe) outperformed all other evaluated compounds. It had a mean IC_50_ of 21 nM, therefore being 1.5-fold more effective than erlotinib (mean IC_50_ = 33 nM) against the four cancer cell lines studied. Compound 21c showed significant antiproliferative activity against the A-549 lung cancer cell line, with an IC_50_ of 20 nM, which is 1.5 times more effective than erlotinib's IC_50_ of 30 nM. Furthermore, compound 21c exhibits a potency 1.4-fold superior to erlotinib against pancreatic (Panc-1) and colon (HT-29) cancer cell lines, and 1.7-fold superior against the breast (MCF-7) cancer cell line, as indicated in Table 1.

The structural features of the heterocyclic component at the fifth position of the pyridine ring (thiophene, thiazole, or thiadiazole), as well as the substitution pattern at the fourth position of the phenyl group in the 1,2,4-oxadiazole moiety, have a significant impact on the novel compounds' antiproliferative activity. For instance, compounds 20c (thiophene scaffold, R = 4-OMe) and 22c (thiadiazole scaffold, R = 4-OMe), which possess thiophene and thiadiazole groups at the fifth position of the pyridine moiety, exhibited decreased efficacy as antiproliferative agents relative to the thiazole derivative, 21c (thiazole scaffold, R = 4-OMe). Compound 20c demonstrated a mean IC_50_ of 24 nM, signifying a potency comparable to that of 21c (mean IC_50_ = 21 nM), while compound 22c displayed a mean IC_50_ value of 56 nM, rendering it 2.7-fold less potent than compound 21c. The data demonstrate that both thiophene and thiazole groups at the 5-position of the pyridine moiety are more advantageous for antiproliferative activity than the thiadiazole group, with activity increasing in the sequence thiazole > thiophene > thiadiazole. Compound 20c (Thiophene scaffold, R = 4-OMe) had the second–highest activity and demonstrated more potency than erlotinib across the four cancer cell lines evaluated, as shown in Table 1.

Additionally, the substitution pattern at the *para* position of the phenyl group in the 1,2,4-oxadiazole moiety may substantially affect the antiproliferative activity of the newly developed compounds. Compounds 21a (thiazole scaffold, R = H) and 21b (Thiazole scaffold, R = 4-Cl), possessing identical structural attributes to 21c but featuring an unsubstituted phenyl group in 21a or a *p*-chlorophenyl group in 21b, had IC_50_ values of 33 nM and 29 nM, respectively. Compound 21a exhibited a GI_50_ value of 33 nM, making it 1.6-fold less effective than 21c. The observations suggest that an unsubstituted phenyl group in the 1,2,4-oxadiazole structure is unfavorable to antiproliferative efficacy. Compound 21b, a *p*-chloro-phenyl derivative of the 1,2,4-oxadiazole moiety, exhibited a mean IC_50_ value of 29 nM, which was slightly less potent than the *p*-methoxy derivative, 21c (mean IC_50_ = 21 nM). This suggests that substitution with either an electron-withdrawing group (chlorine) or an electron-donating group (methoxy) enhances antiproliferative activity, with the methoxy group demonstrating superior efficacy.

Compounds 5 (thiophene-based derivative), 9 (thiazole-based derivative), and 14 (thiadiazole-based derivative), which possess free phenolic hydroxyl groups unassociated with the phenyl-1,2,4-oxadiazole moiety, were identified as the least potent derivatives, exhibiting mean IC_50_ values of 67, 64, and 72 nM, respectively. These values are at least threefold less effective than compound 21c and approximately twofold less potent than erlotinib. The data indicated the potential impact of the 1,2,4-oxadiazole moiety on antiproliferative activity.

#### 3.2.3. EGFR inhibitory assay

The most potent antiproliferative derivatives, 20c, 21a–c, and 22b, were evaluated for their capacity to inhibit EGFR utilizing the EGFR-TK assay.^39^ The findings are displayed in Table 2. Erlotinib functioned as the reference medication. In general, the evaluated compounds 20c, 21a–c, and 22b showed significant EGFR inhibitory activity, with IC_50_ values ranging from 64 to 87 nM, compared with the reference erlotinib, which has an IC_50_ of 80 nM.

**Table 2:** IC_50_ values of compounds 20c, 21a–c, and 22b against EGFR and BRAF^V600E^

Compound	EGFR inhibition IC_50_ ± SEM (nM)	BRAF^V600E^ inhibition IC_50_ ± SEM (nM)
20c	71 ± 4	49 ± 2
21a	82 ± 5	61 ± 4
21b	76 ± 4	54 ± 3
21c	64 ± 3	41 ± 2
22b	87 ± 5	69 ± 4
Erlotinib	80 ± 5	—
Vemurafenib	—	30 ± 2

The results of this assay correspond with those of the antiproliferative assay, demonstrating that compounds 20c (thiophene scaffold, R = 4-OMe) and 21c (thiazole scaffold, R = 4-OMe), recognized as the most potent antiproliferative agents, were the most effective derivatives of EGFR inhibitors, displaying IC_50_ values of 71 ± 4 and 64 ± 3 nM, respectively. They demonstrated 1.2- and 1.3-fold greater efficacy than erlotinib (IC_50_ = 80 nM). Moreover, compounds 21a (Thiazole scaffold, R = H) and 21b (thiazole scaffold, R = 4-Cl) have significant EGFR inhibitory action, with IC_50_ values of 82 and 76 nM, respectively, demonstrating equivalent potency to the reference erlotinib. Ultimately, compound 22b (thiadiazole scaffold, R = 4-Cl) exhibited a slightly reduced EGFR inhibitory efficacy relative to erlotinib, with an IC_50_ value of 87 nM. The data from these *in vitro* investigations suggested that compounds 20c, 21b, and 21c were effective antiproliferative agents, potentially acting as EGFR inhibitors.

#### 3.2.4. BRAF^V600E^ inhibitory assay

The most efficient derivatives, 20c, 21a–c, and 22b, exhibiting significant antiproliferative properties and EGFR inhibitory effects, were further evaluated for their potential to inhibit mutant BRAF, using vemurafenib as the standard.^40^Table 2 presents the results as IC_50_ values. All values are represented as the mean ± standard deviation of three experiments. Compounds 20c, 21b, and 21c demonstrated improved BRAF^V600E^ inhibition, with IC_50_ values of 49, 54, and 41 nM, respectively. All compounds assessed had inferior potency compared to the reference vemurafenib, which has an IC_50_ of 30 nM (Table 2).

Compound 21c, the most efficacious antiproliferative and EGFR inhibitor, demonstrated the highest potency as a BRAF^V600E^ inhibitor, with an IC_50_ of 41 nM, which is 1.4-fold lower than vemurafenib. The results indicated that compounds 20c, 21b, and 21c function as antiproliferative agents targeting both EGFR and mutant BRAF. Nevertheless, they warrant structural modifications to enhance their effectiveness against their molecular targets.

#### 3.2.5. *In vitro* cytotoxicity against normal human cells

Therefore, it was essential to evaluate the safety profiles of the most potent compounds, 20c and 21c, in the normal human diploid cell line (WI-38) using the MTT assay to assess the selectivity of the target compounds for cancer cells *versus* normal cells.^38^ Compounds 20c and 21c exhibited IC_50_ values over 200 nM. The results demonstrated a favorable safety margin for the assessed compounds concerning normal cells, as shown in Table 3.

**Table 3:** IC_50_ values and selectivity index of 20c and 21c against WI-38 normal cell line

Compound	Cytotoxicity (WI-38) IC_50_ (nM)	Selectivity index (SI)	
		HCT-116	A-549
20c	> 200	> 8	> 8
21c	> 200	> 10	> 10

#### 3.2.6. Immunomodulatory assays

Cytokines are integral to the advancement and metastasis of cancer. Extensive research is underway on anti-cytokine therapies, perhaps resulting in innovative treatments for symptoms that are presently challenging to control.^47,48^ Interleukin-6 (IL-6) and tumour necrosis factor-alpha (TNF-α) are multifunctional cytokines implicated in tumour proliferation and metastasis.^49^ TNF-α has been associated with cancer progression and metastasis in both human and experimental animals.^50^ Consequently, anticancer agents that suppress both TNF-α and IL-6 are advantageous for pharmaceutical progress.

Additionally, the dual inhibition of EGFR and BRAF^V600E^ efficiently inhibits the MAPK signaling pathway, and the therapeutic benefit of this combination is increasingly being related to its function in restoring the tumor immune milieu. Dual inhibition greatly reduces the release of major pro-inflammatory cytokines such as IL-6 and TNF-α. These cytokines promote bypass signaling *via* the STAT3 pathway and increase the recruitment of immunosuppressive myeloid cells; thus, studying their regulation is critical for understanding how dual inhibitors overcome initial resistance and restore anti-tumor immunity.

The impact of the most active compounds 20c and 21c on the levels of immunomodulatory proteins (TNF-α and IL-6) was assessed using a qRT-PCR assay test.^51^ A-549 cells were administered compounds 20c and 21c for 24 hours at concentrations of 23 nM and 20 nM (IC_50_ against A-549), respectively. The reference molecule was dexamethasone, a pharmaceutical agent that consistently modulates the immune system.

Compounds 20c and 21c had substantial immunomodulatory effects, reducing the production of pro-inflammatory cytokines TNF-α and IL-6 by 86% and 96%, respectively (Table 4). This degree of inhibition is particularly noteworthy since it surpasses the efficacy of the potent corticosteroid Dexamethasone (83% and 93% inhibition). The superior efficacy of 21c demonstrates that concurrently inhibiting both EGFR and BRAF^V600E^ effectively halts the ERK-dependent activation of NF-κB and AP-1, hence dismantling the cytokine-mediated survival niche that resistant tumor cells frequently exploit for sustenance.

**Table 4:** % Inhibition of compounds 20c and 21c against TNF-α and IL-6

Compound	TNF-α (% inhibition)	IL-6 (% inhibition)
20c	79	89
21c	86	96
Dexamethasone	83	93

#### 3.2.7. Apoptotic markers assays

A hallmark of human cancer is dysregulation of apoptosis, which results in unchecked growth, insufficient response to treatment, and the development of drug-resistant cells.^52,53^ Therefore, it is recognized that modern anticancer medications can cause cancer cells to undergo apoptosis *via* both intrinsic and extrinsic routes.^54^ Therefore, by measuring the expression of crucial apoptotic markers, such as Bcl-2, p53, and Bax, compounds 20c and 21c were investigated for their capacity to cause apoptosis in A-549 lung cancer cells. Table 5 presents the findings.

**Table 5:** Apoptotic assays findings for 20c and 21c against Bax, p53, and Bcl-2

Compound no.	Bcl-2 (ng mL^−1^)	Fold reduction	Bax (pg mL^−1^)	Fold change	p53 (pg mL^−1^)	Fold change
20c	1.70 ± 0.001	3	485 ± 2	8	330 ± 2	5
21c	1.25 ± 0.001	4	550 ± 3	9	450 ± 3	7
Control	5	1	60	1	65	1

Apoptosis is basically controlled by the Bcl-2 protein family, which include anti-apoptotic proteins like Bcl-2 and pro-apoptotic proteins like Bax.^55,56^ Multiple investigations have demonstrated a considerable correlation between high Bcl-2 levels and lowered Bax levels, which contribute to cancer cell proliferation.^57,58^ Thus, we evaluated the Bcl-2 and Bax protein expression levels in A-549 lung cancer cells that were exposed to compounds 20c and 21c.^42^

In comparison to control, untreated cells, compound 20c caused an 8-fold increase in Bax levels and a 3-fold decrease in Bcl-2 levels, as shown in Table 5. Additionally, compound 21c reduced Bcl-2 levels by four times and raised Bax levels nine times. These findings suggest that apoptosis may play a role in the antiproliferative properties of 20c and 21c.

Cancer cells frequently inactivate p53 enzymes during transformation, which could be explained by p53 overexpression's ability to trigger apoptosis.^59^ Cancer cells treated with 20c and 21c demonstrated a significant increase in p53 levels, which were at least five times greater than those of untreated control cells. This data shows that enhanced p53 protein levels may control the apoptotic process in these new drugs.

Moreover, caspase activity dictates the initiation and termination of the apoptosis process.^60^ Caspase-3, an essential enzyme, triggers apoptotic cell death by cleaving numerous proteins within the cell.^61^ Using the A-549 lung cancer cell line, the effects of compounds 20c and 21c on caspase-3 were evaluated and contrasted with staurosporine as a reference drug (Table 6). The findings indicated that 21c was the most potent derivative, exhibiting a significant increase in caspase-3 protein levels (580 ± 4 pg mL^−1^) compared with the reference, staurosporine (465 ± 4 pg mL^−1^). Compound 21c exhibited a 9-fold elevation in active caspase-3 levels compared to control A-549 cells, producing caspase-3 levels that surpassed those induced by staurosporine, the reference medication. Compound 20c demonstrated a sevenfold increase in active caspase-3 levels (455 ± 3 pg mL^−1^) compared to the control untreated A-549 lung cells, as shown in Table 6.

**Table 6:** Caspases 3, 8, and 9 assays of compounds 20c and 21c

Compd. no.	Caspase-3		Caspase-8		Caspase-9	
	Conc. (Pg ml^−1^)	Fold change	Conc. (ng ml^−1^)	Fold change	Conc. (ng ml^−1^)	Fold change
20c	455 ± 3	7	2.05 ± 0.10	20	22 ± 1	22
21c	580 ± 4	9	2.80 ± 0.20	28	25 ± 1	25
Staurosporine	465 ± 4	7	1.90 ± 0.10	19	20 ± 1	20
Control	65	1	0.10	1	1	1

The effects of compounds 20c and 21c on caspase-8 and caspase-9 were investigated in order to determine whether these medicines cause apoptosis *via* the intrinsic or extrinsic pathways. In comparison to the control untreated A-549 cancer cells, the results showed that compound 20c elevates caspase-8 and caspase-9 levels by 20 and 22 times, respectively, while compound 21c increases caspase-8 and caspase-9 levels by 28 and 25 times. This signifies the activation of both intrinsic and extrinsic pathways, as shown in Table 6.

### 3.3. *In silico* studies

#### 3.3.1. Docking study into the EGFR active site

The most effective antiproliferative derivatives against EGFR, 20c and 21c, were docked in order to examine the molecular basis for EGFR inhibition by the newly developed compounds (PDB ID: 1M17).^62,63^ The FDA-approved inhibitor erlotinib acted as the reference ligand for EGFR. Protein structures were obtained from the Protein Data Bank and processed using the Discovery Studio 2016, utilizing the Avogadro force field.^64^ The precision of the docking process was validated using self-docking, wherein the native ligand (erlotinib) was re-docked into its original EGFR binding site. The resulting RMSD of 1.44 Å and a re-docking score of −7.4 kcal mol^−1^ demonstrated a significant association with the experimentally observed conformation, so validating the reliability and predictive accuracy of the docking methodology.

**Fig. 4:**
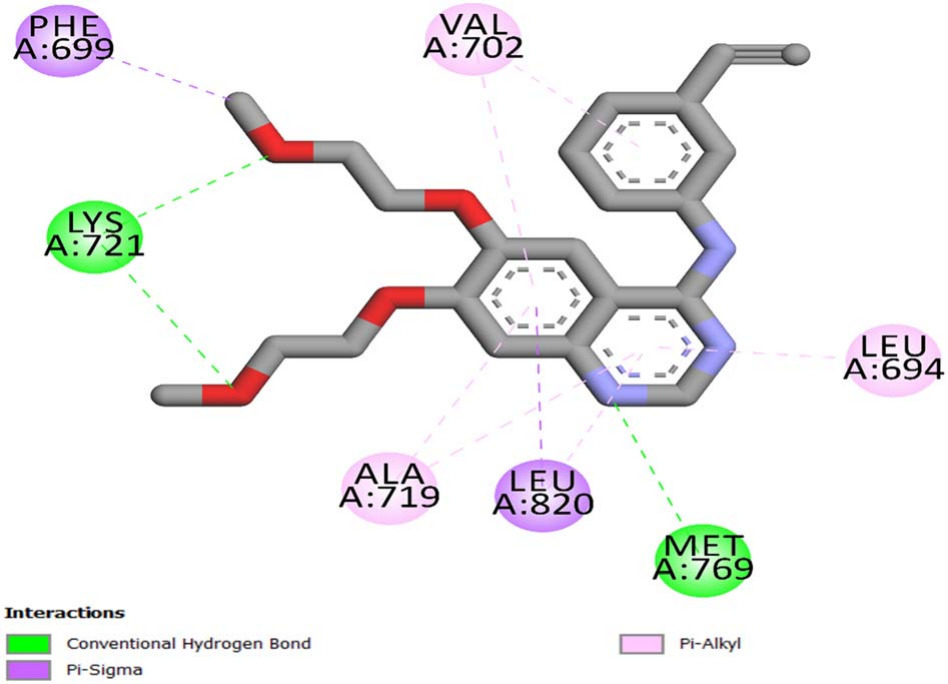
Two-dimensional (2D) interaction mapping of erlotinib within EGFR active site.

The traditional hinge-region hydrogen bond between the docked ligands' pyrimidine nitrogen and EGFR Met769 was reliably detected, demonstrating its crucial function in compound stabilization within the ATP-binding pocket. Erlotinib established an additional hydrogen bond with Lys721 *via* the ethoxy group, as well as a network of hydrophobic bonds comprising Leu694, Phe699, Val702, Ala719, and Leu820 (Fig. 4).

**Fig. 5:**
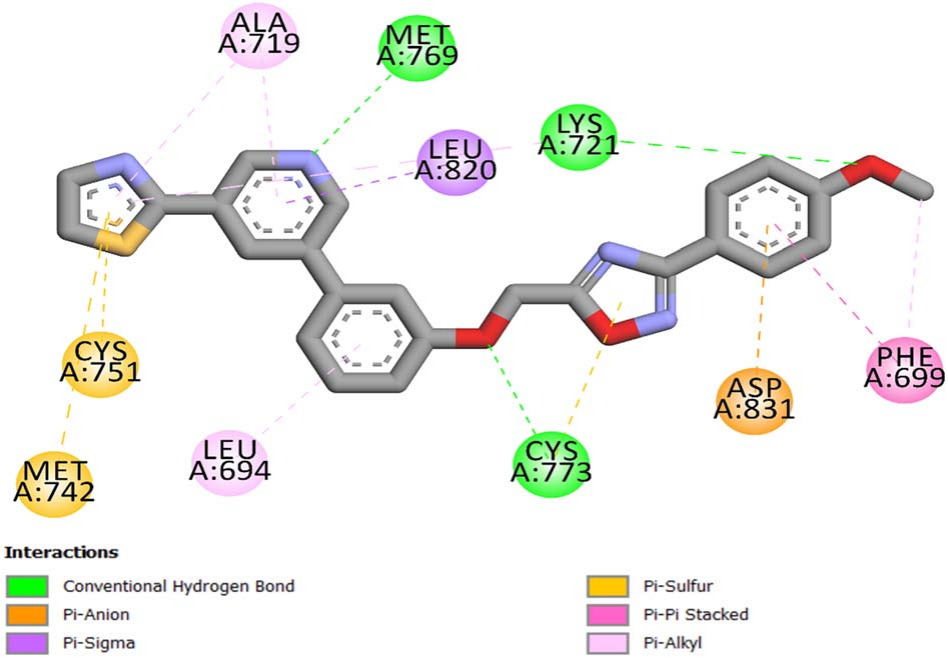
Compound 21c's 2D interaction mapping with the EGFR active site.

Compound 21c possesses a distinct pharmacophoric structure with two principal components: the phenyl-1,2,4-oxadiazole ring and the pyridine ring. Each characteristic specifically enhances the elevated binding affinity within the ATP-binding pocket of EGFR. The pyridine ring functions as the primary anchoring motif, forming a crucial hydrogen bond with the hinge residue Met769, a conserved interaction necessary for efficient ATP-competitive kinase inhibition. This ring is preserved in a planar conformation, essential for its accurate alignment within the tight hinge region. Planarity is enhanced by the neighboring thiazole group, which stabilizes the entire compound's binding *via* two pi-sulfur interactions with Cys751 and Met742 (Fig. 5). Adjacent to the pyridine ring, the *p*-methoxyphenyl-1,2,4-oxadiazole ring facilitates molecular recognition by two hydrogen bonds with Lys721 and Cys773, *via* the methoxy group and the oxygen atom of the 1,2,4-oxadiazole ring. Furthermore, the phenyl ring of the 1,2,4-oxadiazole moiety engages in a pi–anion interaction with Asp831, hence augmenting binding stability and directional specificity. Ultimately, the entire molecule contributes to a network of hydrophobic contacts with Leu694, Phe699, Ala719, and Leu820, as illustrated in Fig. 5. These combined effects clarify the strong binding affinity (−9.5 kcal mol^−1^ and RMSD of 1.46) observed in the docking tests and the significant *in vitro* inhibitory activity demonstrated by 21c, which surpassed that of the reference erlotinib.

**Fig. 6:**
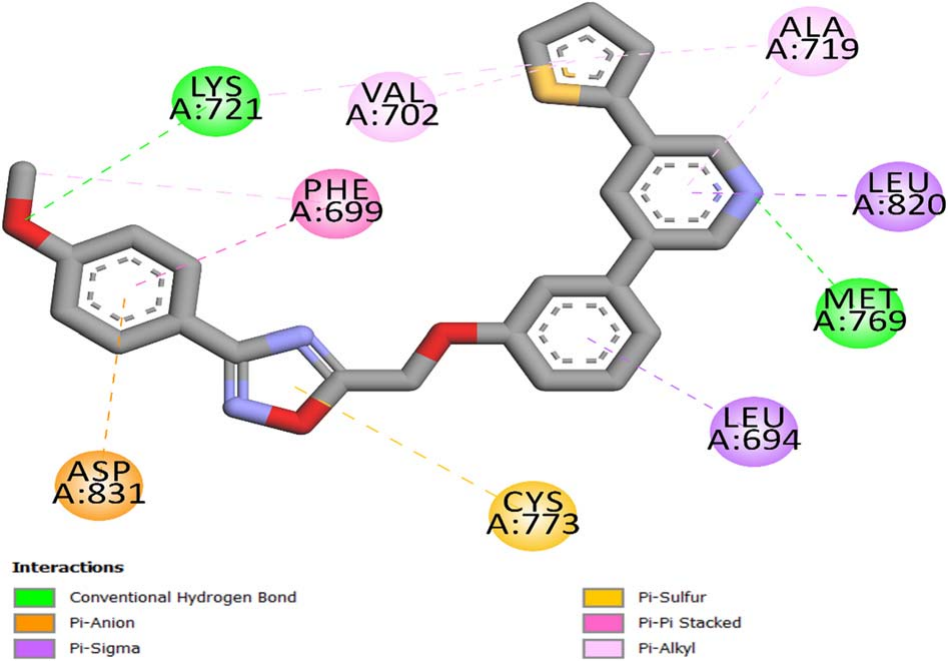
Compound 20c's 2D interaction mapping with the EGFR active site.

Compound 20c's docking investigation within the EGFR binding site revealed that it has a binding interaction pattern identical to compound 21c, with the difference that, unlike compound 21c, the binding with residue Cys773 is accomplished *via* a pi-sulfur link rather than a hydrogen bond, Fig. 6. This elucidates the decreased binding affinity of 20c (−9.3 kcal mol^−1^) relative to 21c (−9.5 kcal mol^−1^) and, consequently, its reduced efficacy. Nonetheless, both 20c and 21c have greater potency than erlotinib.

#### 3.3.2. Docking study into BRAF^V600E^ active site

**Fig. 7:**
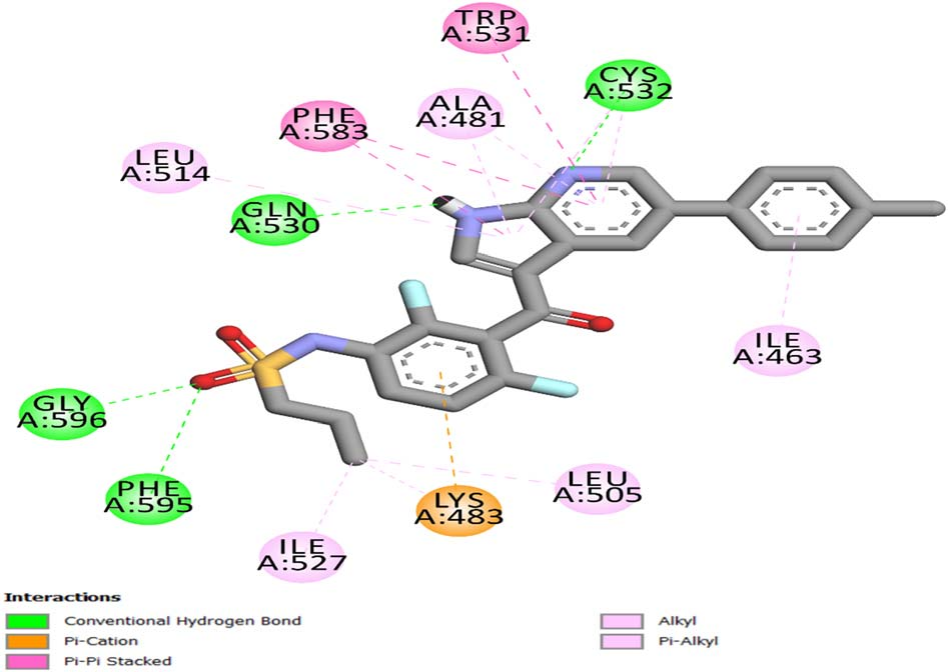
Vemurafenib's two-dimensional interaction mapping with the BRAF^V600E^ active site.

To verify the docking protocol for BRAF^V600E^, the co-crystallized ligand, vemurafenib, was re-docked into its binding site (PDB ID: 3OG7).^65,66^ The redocking produced a docking score of −11 kcal mol^−1^ and an RMSD of 1.53 Å relative to the experimental conformation, indicating remarkable agreement and affirming the dependability of the docking protocol. The consistent binding orientation of vemurafenib preserved the critical hydrogen bonds with Cys532 and Gly596, vital interactions that stabilize inhibitors in the ATP-binding site, as depicted in Fig. 7. Vemurafenib also formed two hydrogen bonds with Gln530 and Phe595, as well as a network of hydrophobic bonds that included Trp531, Phe583, and Ala481.

**Fig. 8:**
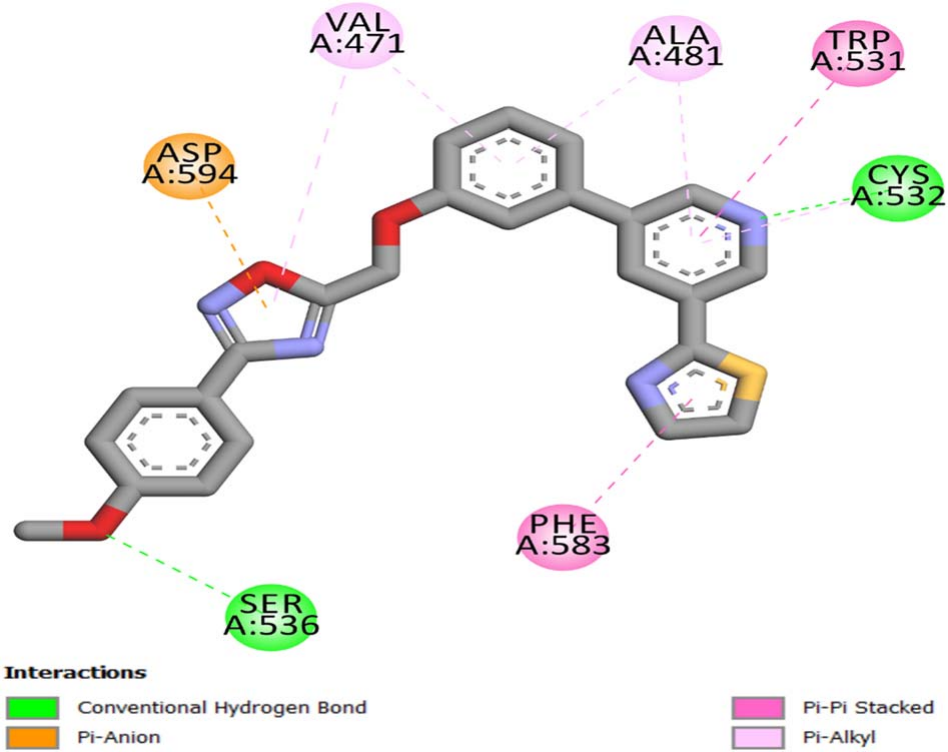
Compound 21c's two-dimensional interaction mapping with BRAF^V600E^'s active site.

With a docking score of −9.20 kcal mol^−1^ and an RMSD of 1.57 Å from the reference pose, 21c's docking into the ATP-binding site of BRAF^V600E^ exhibited a well-aligned and energetically favorable binding configuration. The ligand is firmly positioned within the active site, where the pyridine ring forms a hydrogen bond with the backbone of Cys532 and engages in hydrophobic interactions with Trp531, Ala481, and Phe583, as depicted in Fig. 8. Additionally, the oxadiazole ring forms a hydrogen bond with Ser536 *via* the *p*-methoxy group, consistent with structure–activity relationship trends indicating increased potency with *p*-methoxy substitution. Furthermore, it engages in a π–anion interaction with Asp594 and a π–π-alkyl interaction with Val471, thereby stabilizing the scaffold's position within the hinge region.

#### 3.3.3. Swiss ADME prediction

To assess developability in relation to target potency, we evaluated compound 21c using SwissADME^67,68^ and compared the results with the clinical standard erlotinib. 21c is defined as a moderate, lipophilic scaffold (MW 442.49 g mol^−1^; consensus log *P* 4.44) exhibiting high polarity (TPSA 111.40 Å^2^), comprising seven rotatable bonds, seven hydrogen-bond acceptors, and no hydrogen-bond donors. All solubility models indicate low to moderate aqueous solubility (ESOL logS −5.62; Ali −6.58; SILICOS-IT −9.61), and gastrointestinal absorption is anticipated to be minimal. The compound serves as a P-glycoprotein substrate and lacks blood–brain barrier permeability; yet it is anticipated to inhibit CYP2C9 and CYP3A4, indicating a potential risk for drug–drug interactions. The criteria for rule-based drug-likeness are fully met Lipinski's criteria are satisfied without violations (MW < 500), and the filters set by Ghose, Veber, Egan, and Muegge are satisfied (TPSA < 140 Å^2^ and < 10 rotors); the bioavailability score is 0.55. Alerts in medicinal chemistry are negligible (PAINS 0; Brenk alert 0). A synthetic accessibility (SA) score of 3.70 is deemed low to moderate, suggesting that the molecule is anticipated to be comparatively easy to synthesize.

Erlotinib, used as a reference, has an oral-drug-like profile (MW 393.4 g mol^−1^, consensus log *P* 3.20; TPSA 74.7 Å^2^; 10 rotatable bonds) with model-dependent but acceptable solubility (ESOL log *S* −4.11; Ali −4.56; SILICOS-IT −7.26) and predicted high GI absorption. It is not a P-gp substrate and is BBB permeant. Erlotinib meets the Lipinski, Ghose, Veber, Egan, and Muegge criteria (bioavailability score 0.55), has no PAINS alerts and one Brenk alert (alkyne), and is synthetically accessible (3.19). However, broad CYP inhibition (1A2/2C19/2C9/2D6/3A4) is envisaged for this chemotype.

Taken together, the ADME comparison reveals that 21c's potency is accompanied by a suboptimal physicochemical envelope, moderate MW, high lipophilicity, and high TPSA, all of which contribute to poor solubility and low predicted intestinal absorption and increase the likelihood of CYP2C9/3A4 interactions.

### 3.4. Structural activity relationship (SAR) analysis

The subsequent points delineate the structure–activity relationship of the newly synthesized compounds 5, 9, 14, 20a–c, 21a–c, and 22a–c.
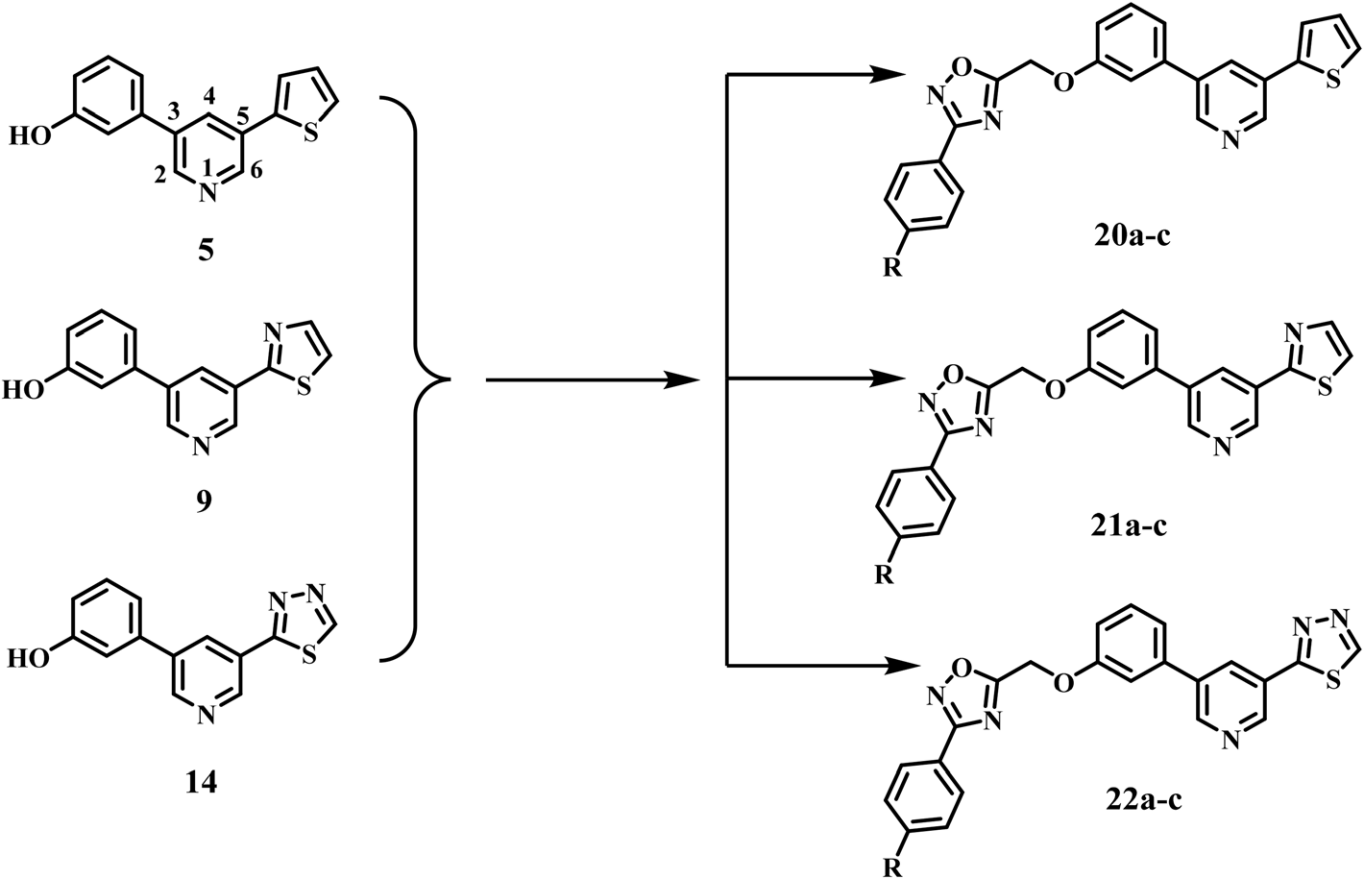


1- The phenyl-1,2,4-oxadiazole moiety is crucial for activity. Compounds 5 (thiophene-based derivative), 9 (thiazole-based derivative), and 14 (thiadiazole-based derivative), which are characterized by the presence of free phenolic moiety at position-3 of pyridine, were identified to be the least potent derivatives.

2- The pyridine ring is vital for activity, as the nitrogen atom forms a hydrogen bond with the crucial amino acid Met769, underscoring its significant role in stabilizing compounds within the ATP-binding pocket. It similarly establishes a hydrogen bond with Cys352, an essential interaction for the inhibitory activity of BRAF^V600E^, akin to that observed in Vemurafenib.

3- The pyridine ring engaged in a series of hydrophobic interactions within the binding sites of both EGFR and BRAF^V600E^, therefore consolidating the compounds in their receptor binding sites and augmenting their efficacy.

4- The coexistence of thiophene and thiazole groups at the 5-position of the pyridine moiety enhances antiproliferative activity more effectively than the thiadiazole group, with activity ranking as thiazole > thiophene > thiadiazole.

5- The unsubstituted phenyl group in the 1,2,4-oxadiazole structure reduces antiproliferative efficacy. Substitution with either an electron-withdrawing group (chlorine atom) or an electron-donating group (methoxy group) enhances antiproliferative activity, with the methoxy group demonstrating superior efficacy.

6- The methoxy group in the phenyl-1,2,4-oxadiazole moiety engages in additional hydrogen bonds with Lys712 in the EGFR binding site and Ser536 in the BRAF^V600E^ binding pocket, thereby further stabilizing the compounds within the receptor binding sites and enhancing their efficacy.

## 4. Conclusion

This work presents the successful design and synthesis of novel thiophene/thiazole/thiadiazole-1,2,4-oxadiazole derivatives that are potential dual inhibitors of EGFR and BRAF^V600E^, introducing a new scaffold with considerable therapeutic significance in cancer treatment. Among the synthesized compounds, 20c and 21c emerged as the most promising lead compounds, demonstrating significant antiproliferative activity against specific human cancer cell lines while preserving a favorable safety profile in normal human cell lines. Compared to Erlotinib, compounds 20c and 21c exhibited superior inhibitory potency against EGFR and comparable efficacy to Vemurafenib against BRAF^V600E^. Compounds 20c and 21c exhibited apoptotic and immunomodulatory characteristics. Molecular docking experiments validated the dual-targeting strategy of the examined compounds *via* essential interactions within the active sites of EGFR and BRAF^V600E^.

These findings underscore 20c and 21c as promising lead compounds for subsequent preclinical development. Subsequent research will focus on the structural optimization of analogous compounds and *in vivo* assessments to improve potency, selectivity, and therapeutic efficacy, thereby advancing innovative dual-targeted anticancer treatments.

## Conflicts of interest

The authors disclose no conflicting interests.

## Supplementary Material

RA-016-D6RA00207B-s001

## Data Availability

The authors assert that any data supporting this study can be found in the supplementary materials (SI). Supplementary information is available. See DOI: https://doi.org/10.1039/d6ra00207b.
