# Supplementary material for: Design, synthesis and antiproliferative, apoptotic, and immunomodulatory properties of new heteroaryl pyridine-linked 1,2,4-oxadiazoles as prospective dual EGFR/BRAFV600E inhibitors
Source: RSC Adv. 2026 Mar 16;16(16):14294–309. doi: 10.1039/d6ra00207b (PMC12990325; doi:10.1039/d6ra00207b)
Supplement: RA-016-D6RA00207B-s001 [file RA-016-D6RA00207B-s001.pdf]

Supplementary Information (SI) for RSC Advances.
This journal is © The Royal Society of Chemistry 2026

Design, synthesis, antiproliferative, apoptotic, and immunomodulatory properties of new heteroaryl pyridine-linked 1,2,4-oxadiazoles as prospective dual EGFR/BRAF^{V600E} inhibitors

Hesham A. M. Gomaa[1]*, Mohamed E. Shaker[1], Sami I. Alzarea[1], Eid Alatwi[1], Fatma A.M. Mohamed[2], Abdullah Yahya Abdullah Alzahrani[3], Bandar A. Alyami[4], Stefan Bräse[5]*, Safwat M. Rabea[6], Bahaa G. M. Youssif[7]*

[1]Department of Pharmacology, College of pharmacy, Jouf University, Sakaka 72388, Saudi Arabia; [2]Department of Clinical Laboratory Sciences, College of Applied Medical Sciences at Al-Qurayyat, Jouf University, Al-Qurayyat 72388, Saudi Arabia; [3]Department of Chemistry, Faculty of Science, King Khalid University, Abha 61413, Saudi Arabia; [4]Department of Pharmaceutical Chemistry, College of Pharmacy, Najran University, Najran, Saudi Arabia; [5]Institute of Biological and Chemical Systems, IBCS-FMS, Karlsruhe Institute of Technology, 76131 Karlsruhe, Germany; [6]Medicinal Chemistry Department, Faculty of Pharmacy, Minia University, Minia 61519, Egypt. [7]Pharmaceutical Organic Chemistry Department, Faculty of Pharmacy, Assiut University, Assiut 71526, Egypt

**To whom correspondence should be addressed:*

Hesham A.M. Gomaa, Ph.D. Department of Pharmacology, College of Pharmacy, Jouf University, Sakaka 72388, Saudi Arabia.

E-mail address: hasoliman@ju.edu.sa

Bahaa G. M. Youssif, Ph.D. Pharmaceutical Organic Chemistry Department, Faculty of Pharmacy, Assiut University, Assiut 71526, Egypt.

Tel.: +201044353895; E-mail address: bgyoussif2@gmail.com

Stefan Bräse

Institute of Biological and Chemical Systems, IBCS-FMS, Karlsruhe Institute of Technology, 76131 Karlsruhe, Germany. E-mail address: braese@kit.edu

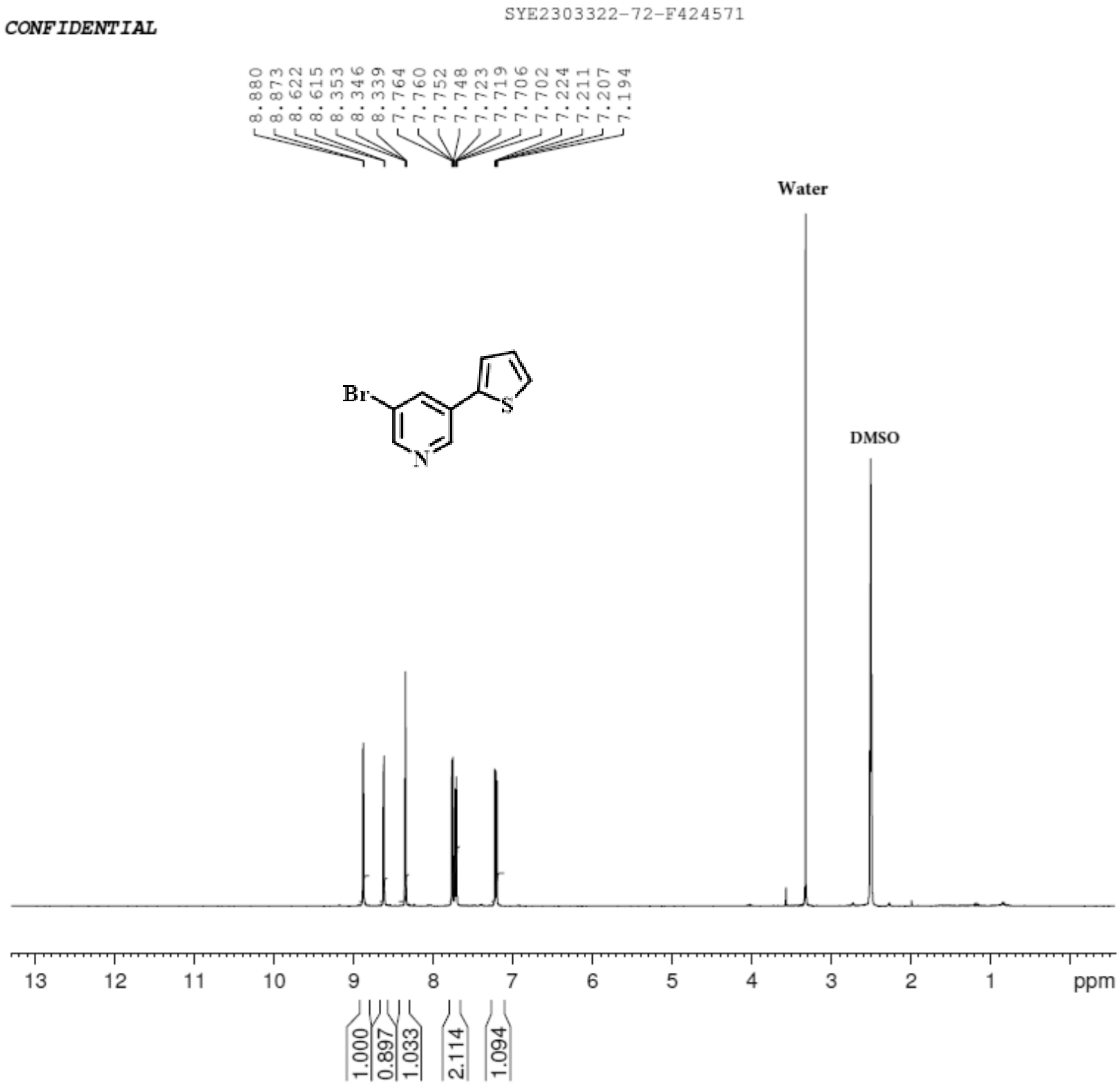

Figure S1: ^{1}H NMR (400 MHz, DMSO-d_6) of compound **3**

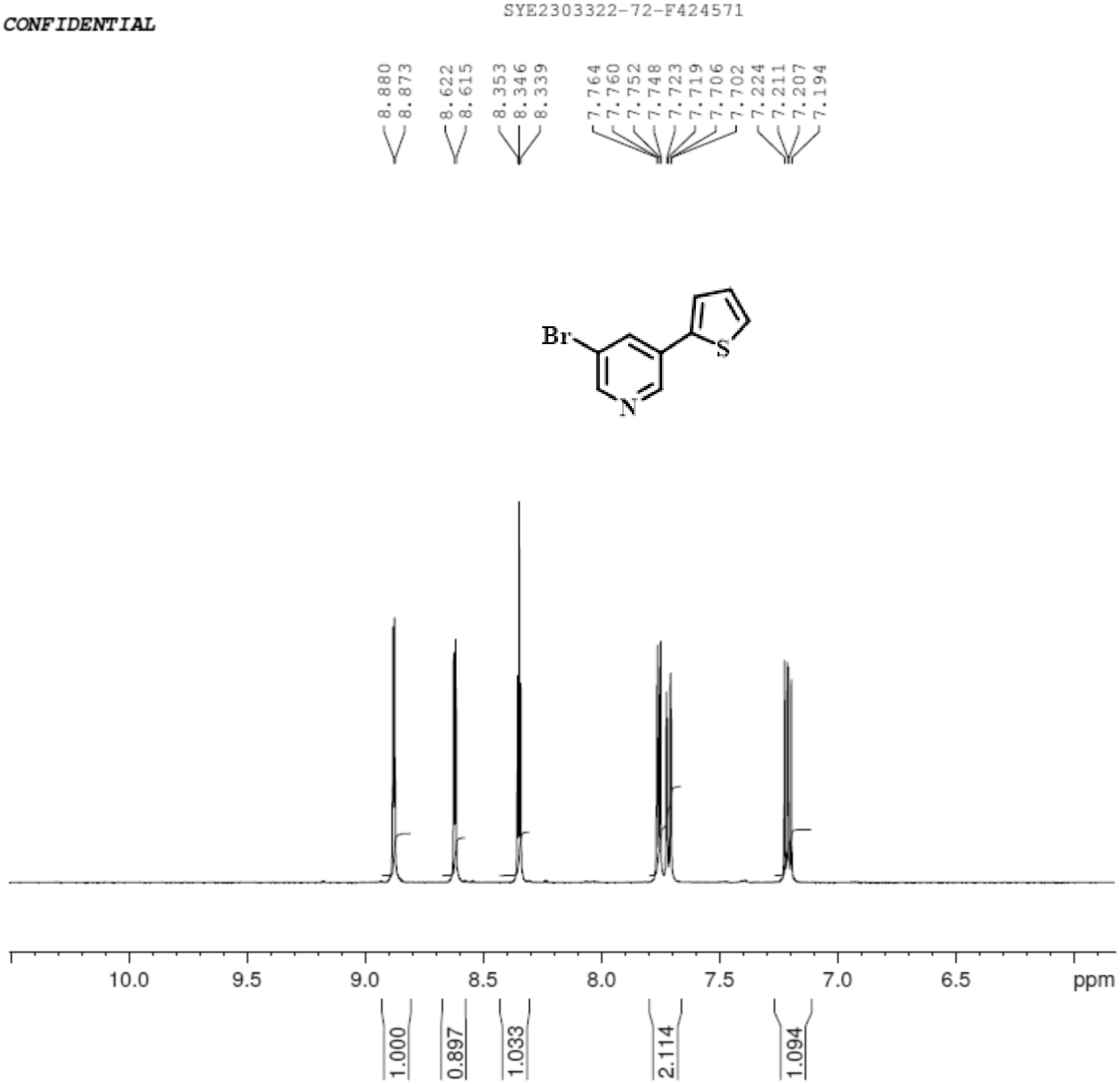

Figure S2: Expanded ^{1}H NMR (400 MHz, DMSO-d_6) of compound **3**

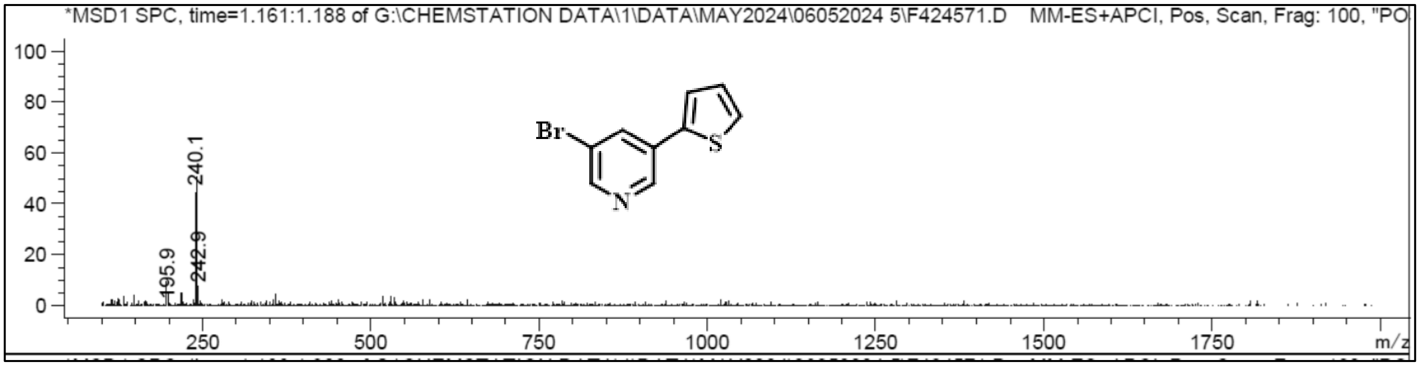

Figure S3: LCMS of compound **3**

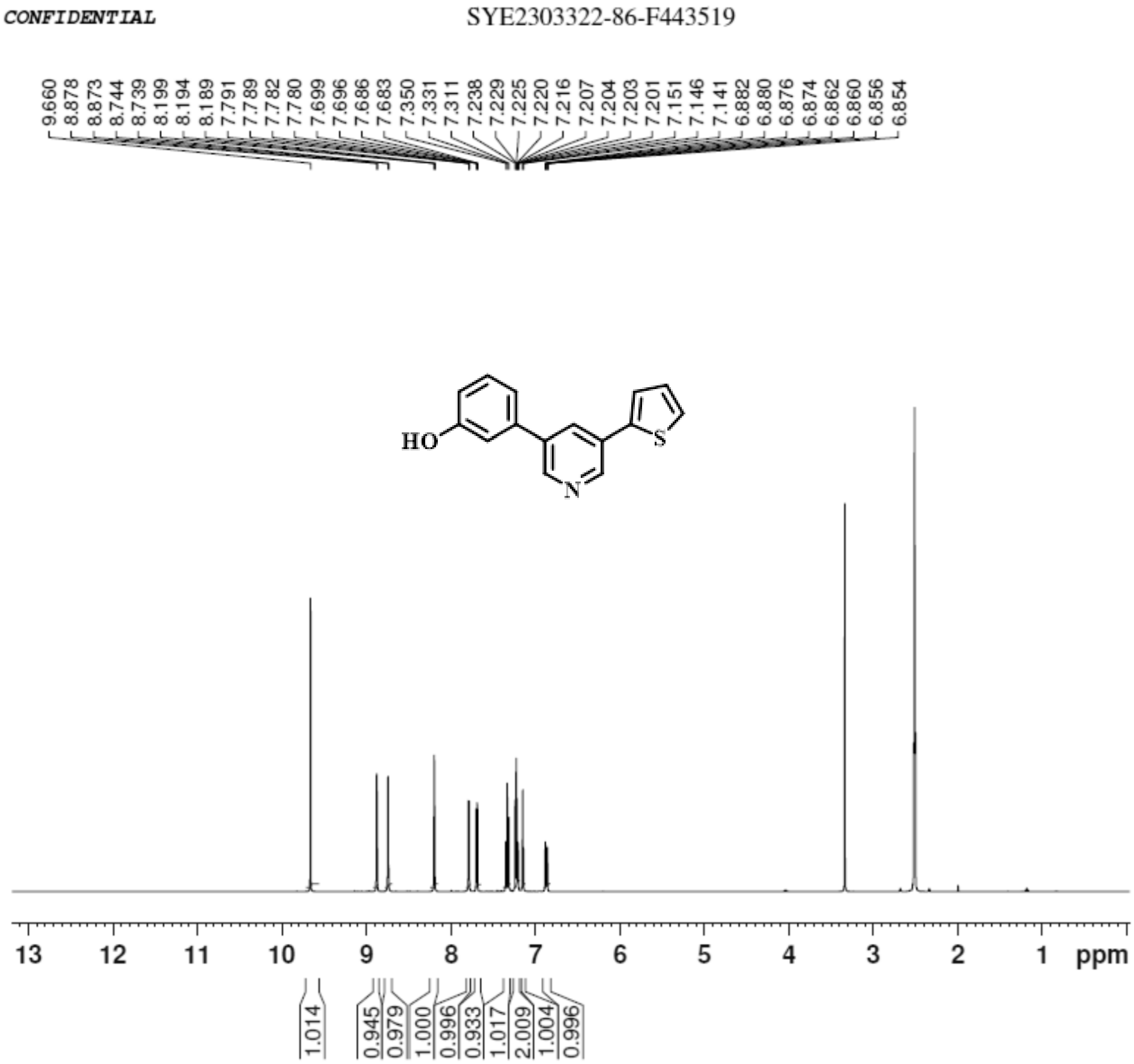

Figure S4: ^{1}H NMR (400 MHz, DMSO-d_6) of compound **5**

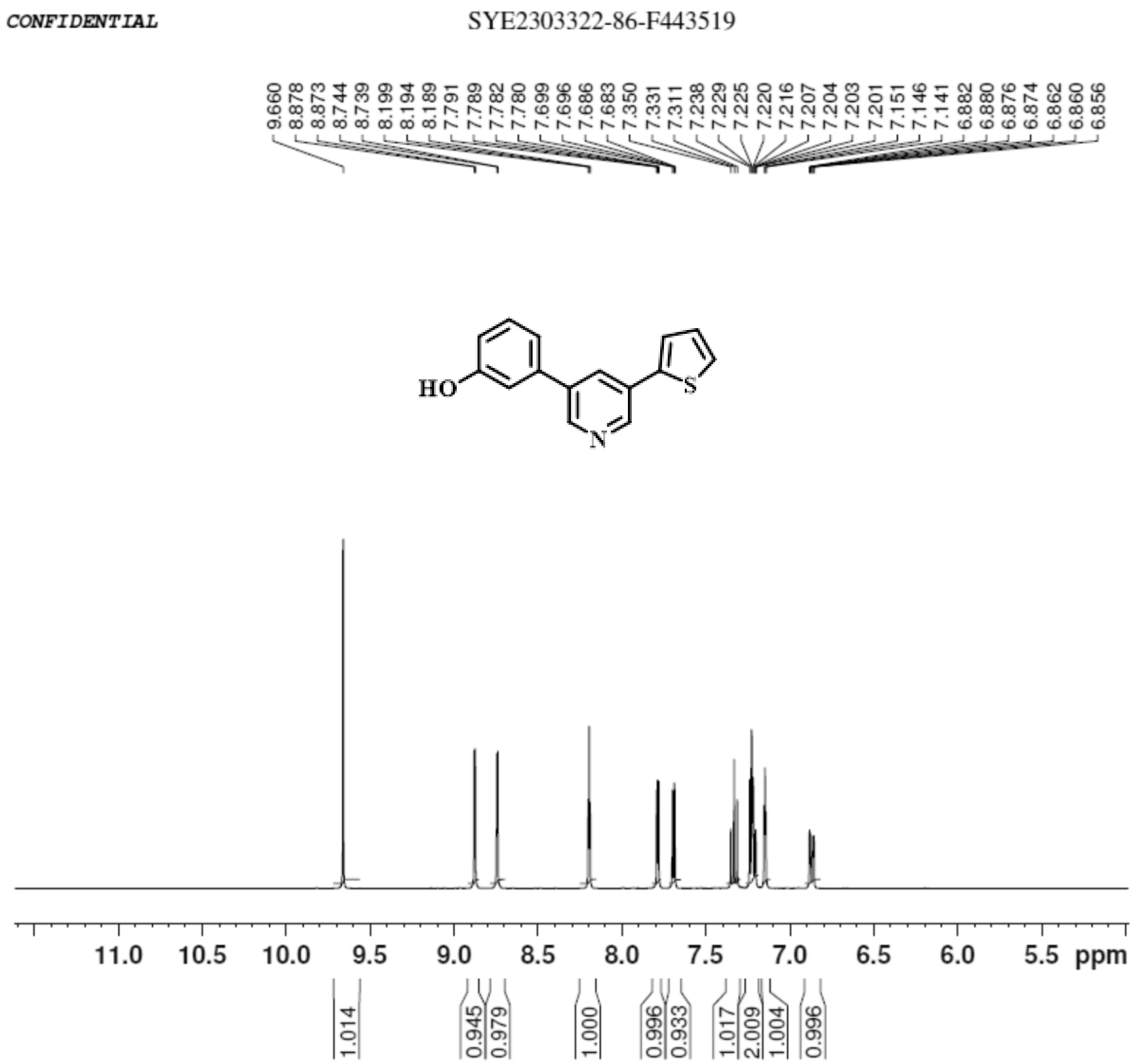

Figure S5: Expanded ^{1}H NMR (400 MHz, DMSO-d_6) of compound **5**

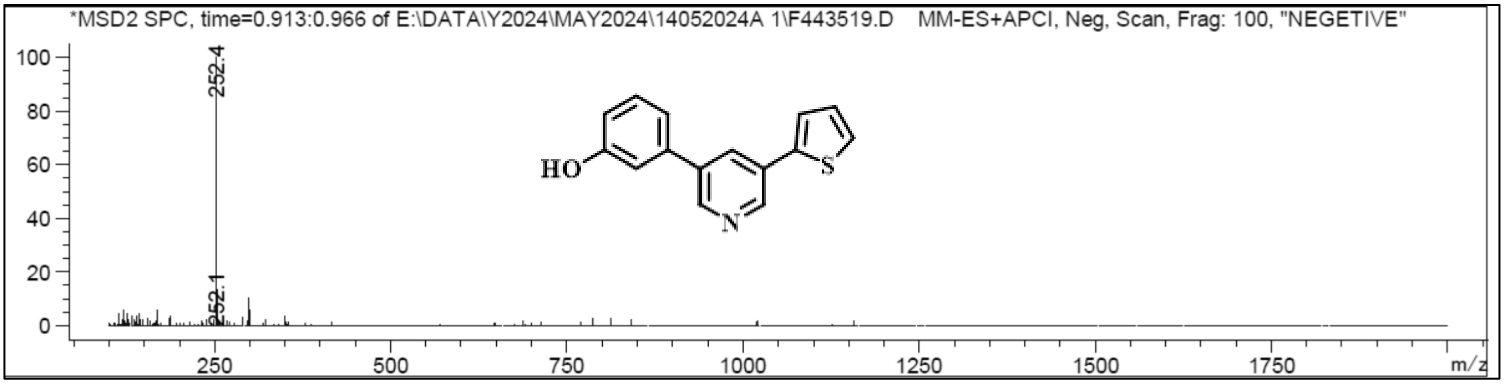

Figure S6: LCMS of compound **5**

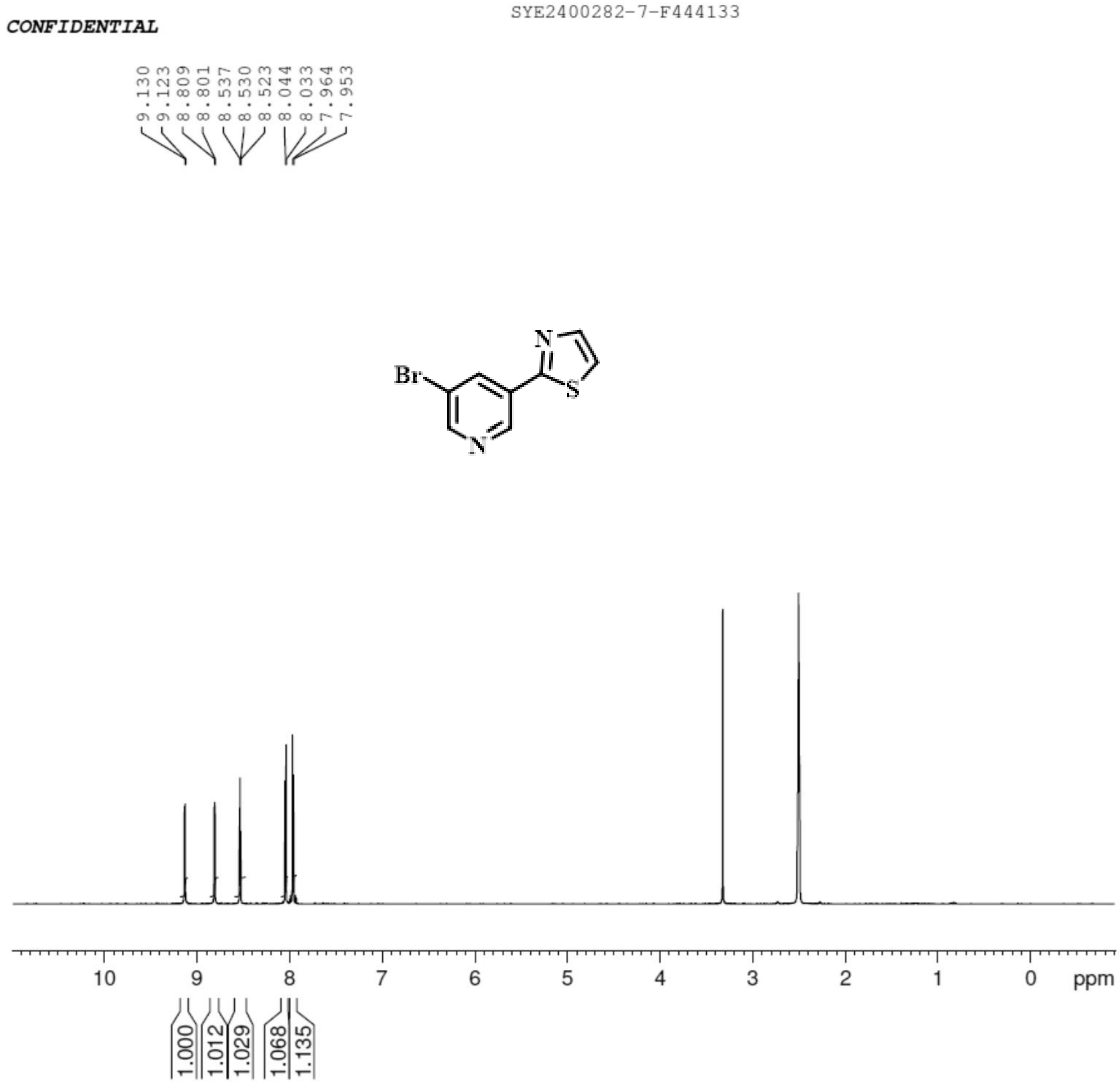

Figure S7: ^{1}H NMR (400 MHz, DMSO-d_6) of compound **8**

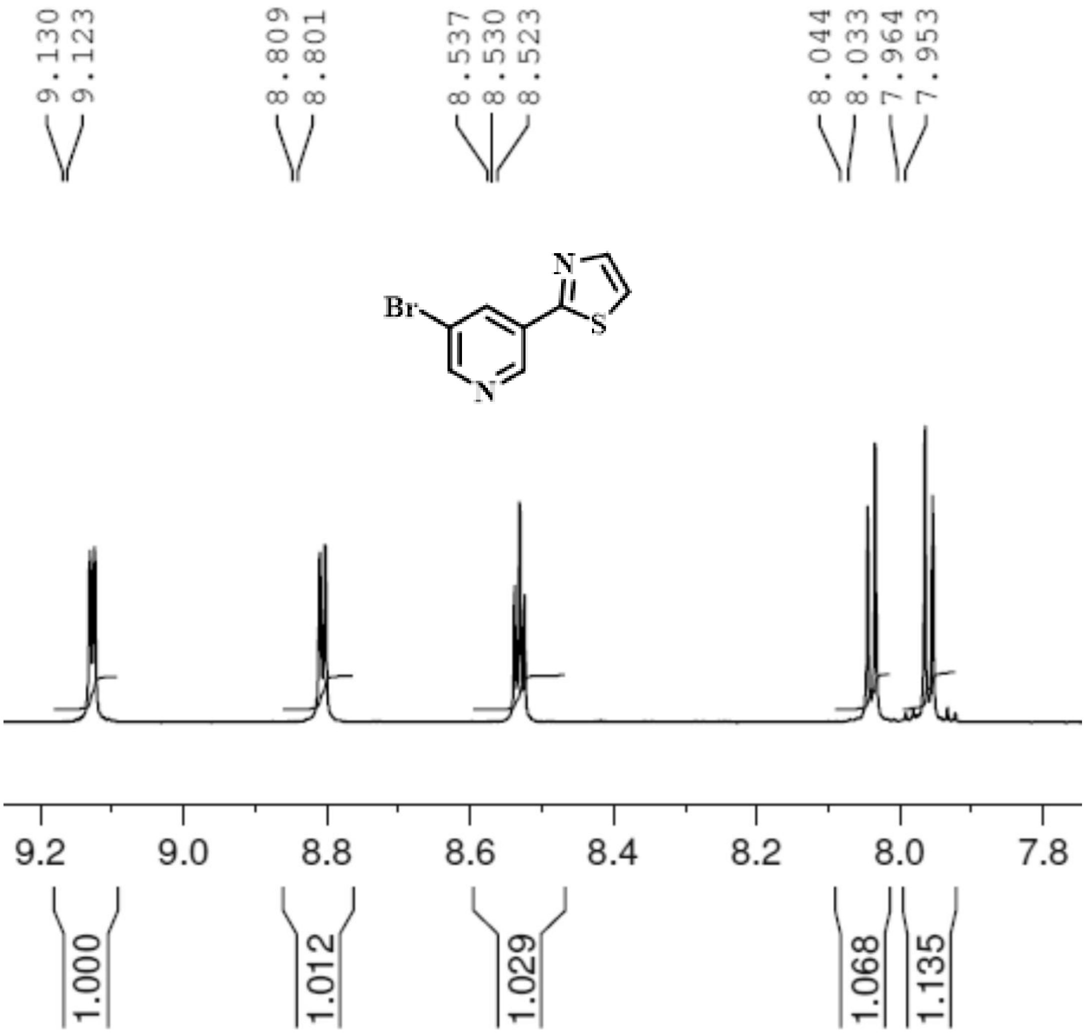

Figure S8: Expanded ^{1}H NMR (400 MHz, DMSO-d_6) of compound **8**

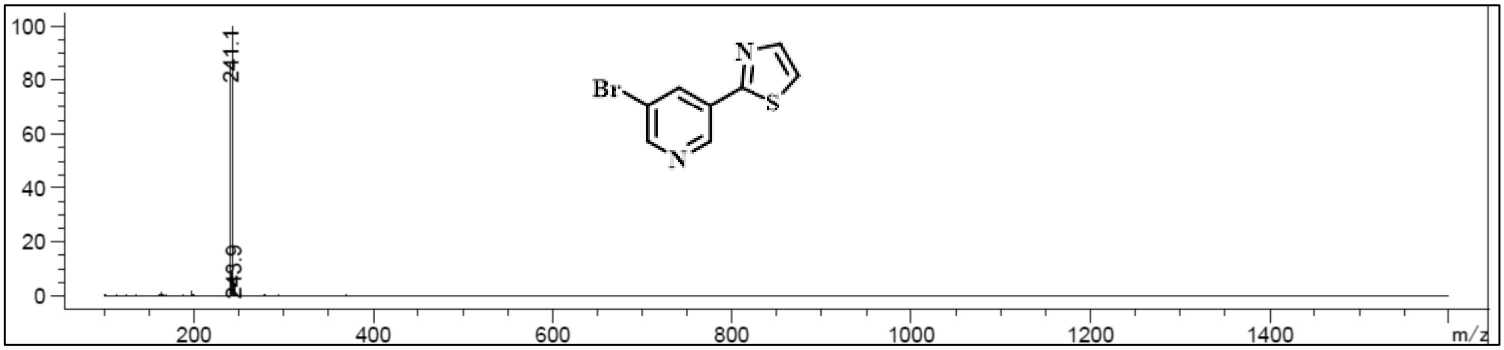

Figure S9: LCMS of compound **8**

CONFIDENTIAL

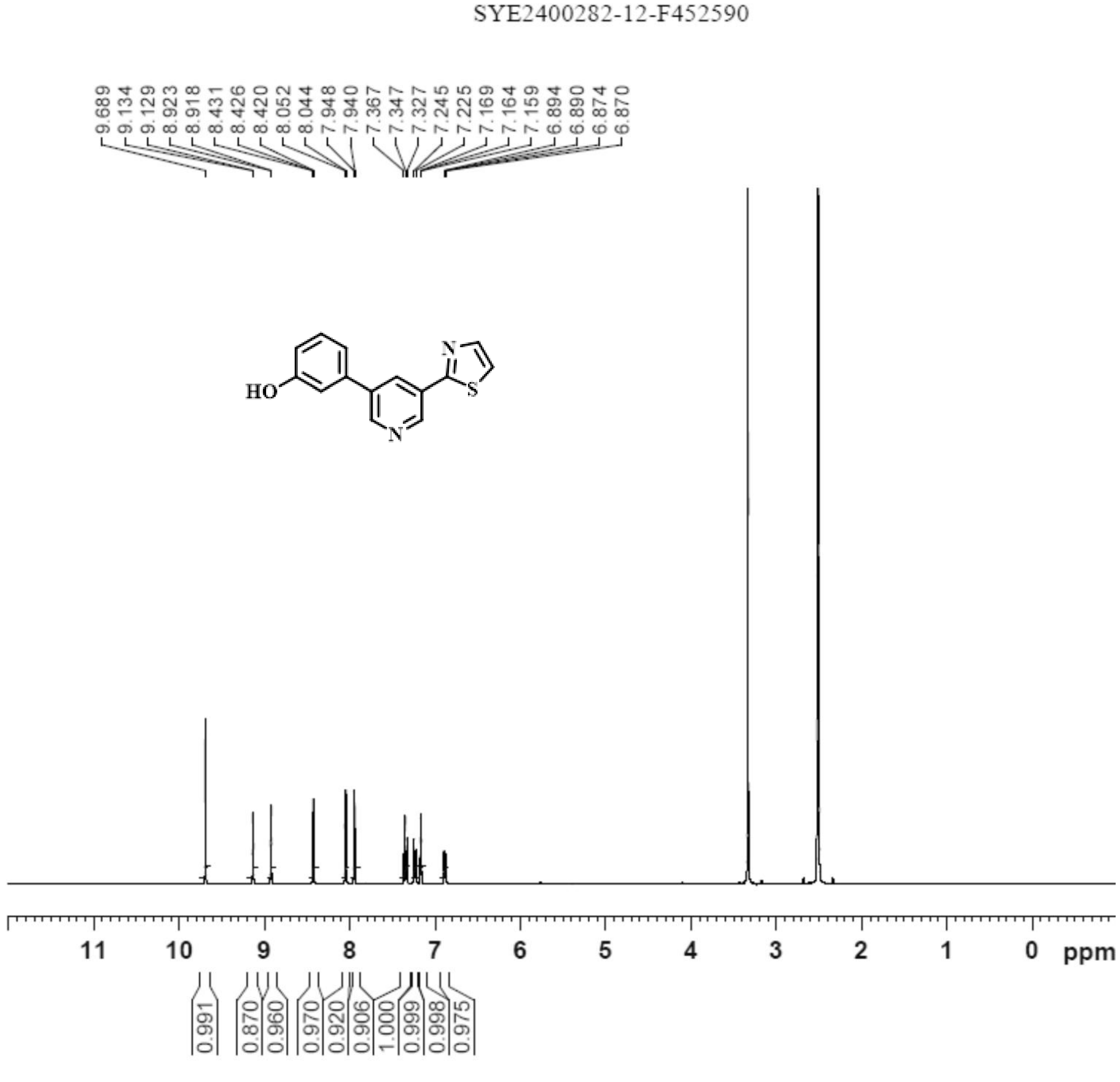

Figure S10: ^{1}H NMR (400 MHz, DMSO-d_6) of compound **9**

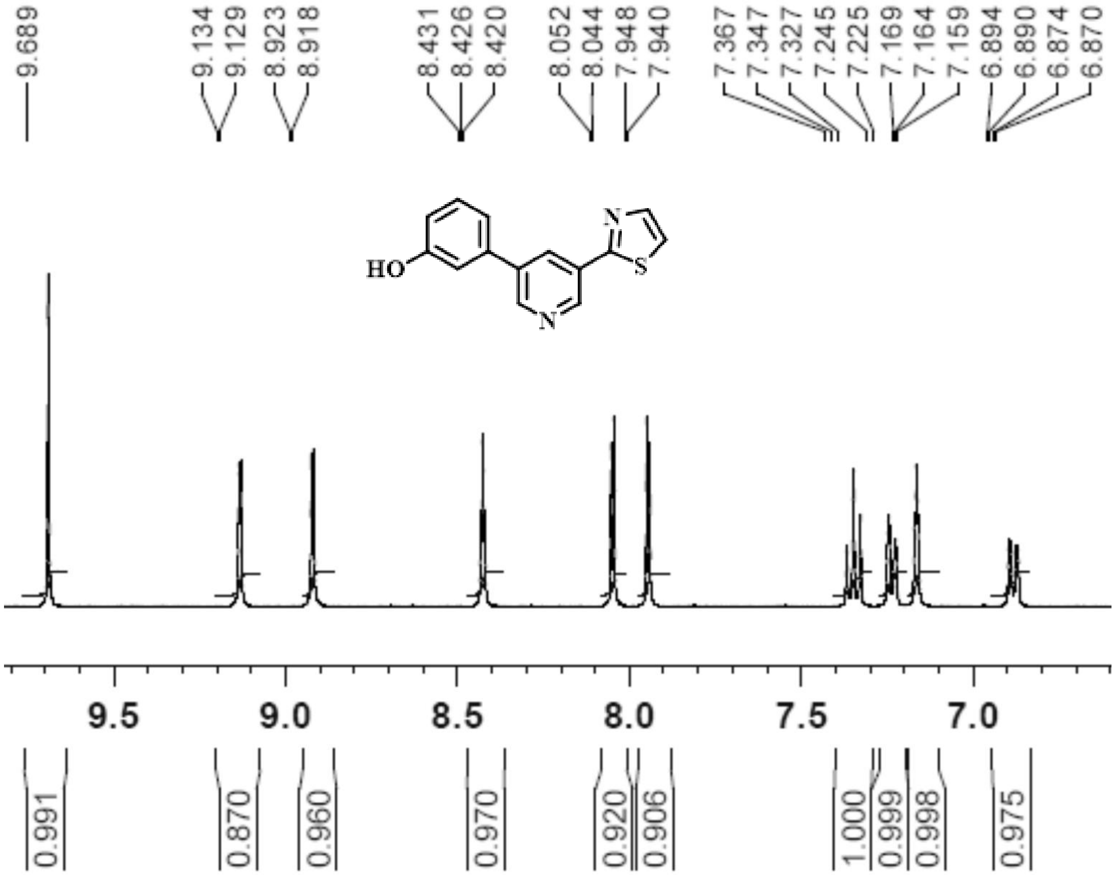

Figure S11: Expanded ^{1}H NMR (400 MHz, DMSO-d_6) of compound **9**

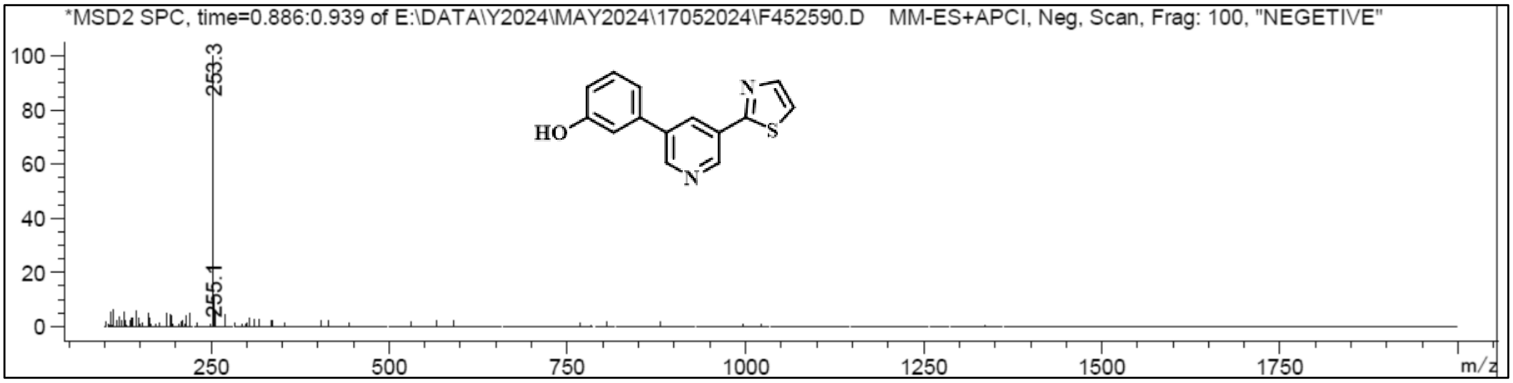

Figure S12: LCMS of compound **9**

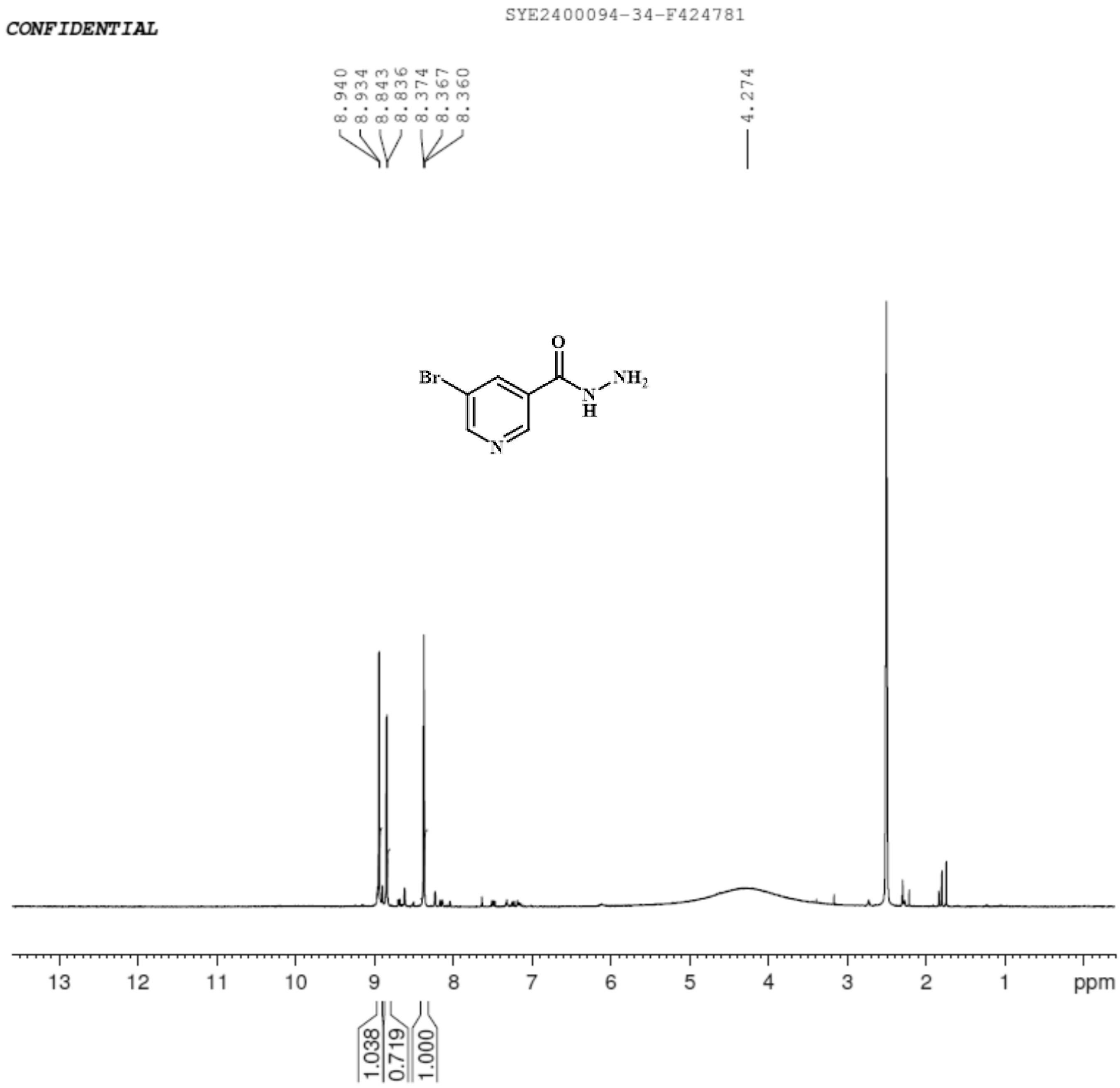

Figure S13: ^{1}H NMR (400 MHz, DMSO-d_6) of compound **11**

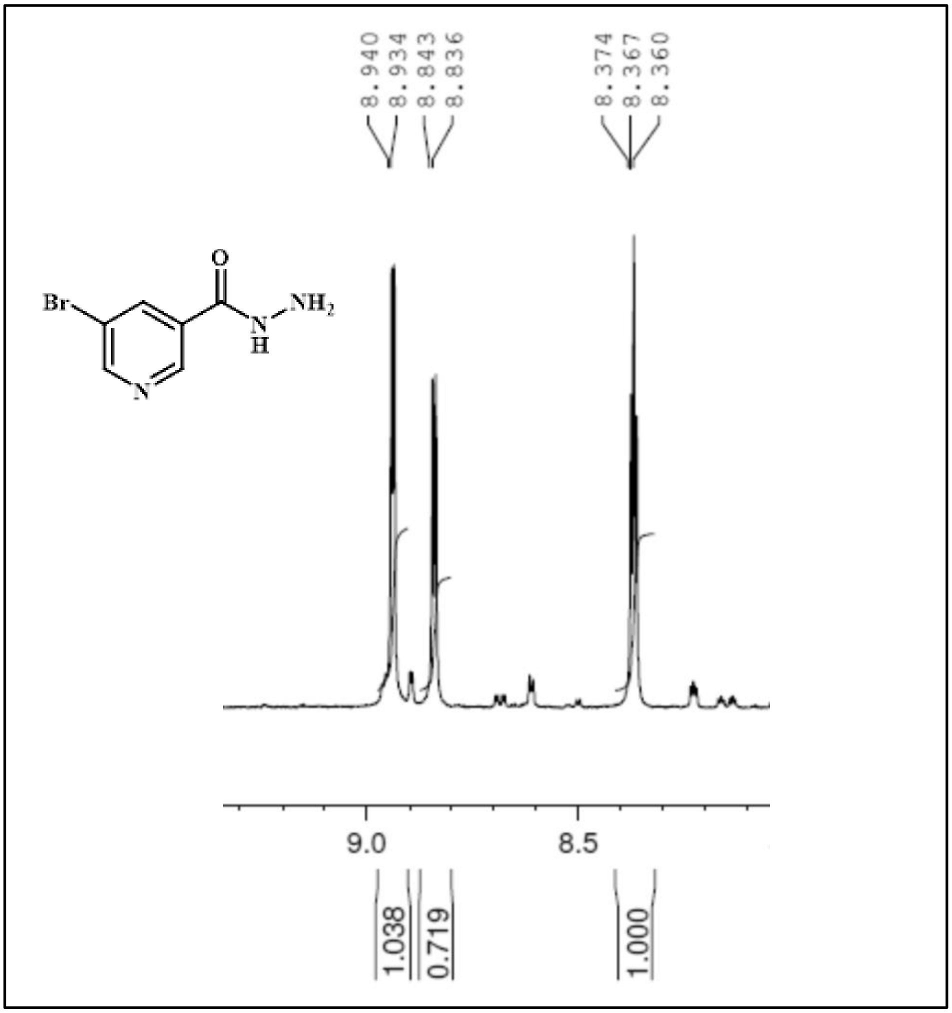

Figure S14: Expanded ^{1}H NMR (400 MHz, DMSO-d_6) of compound **11**

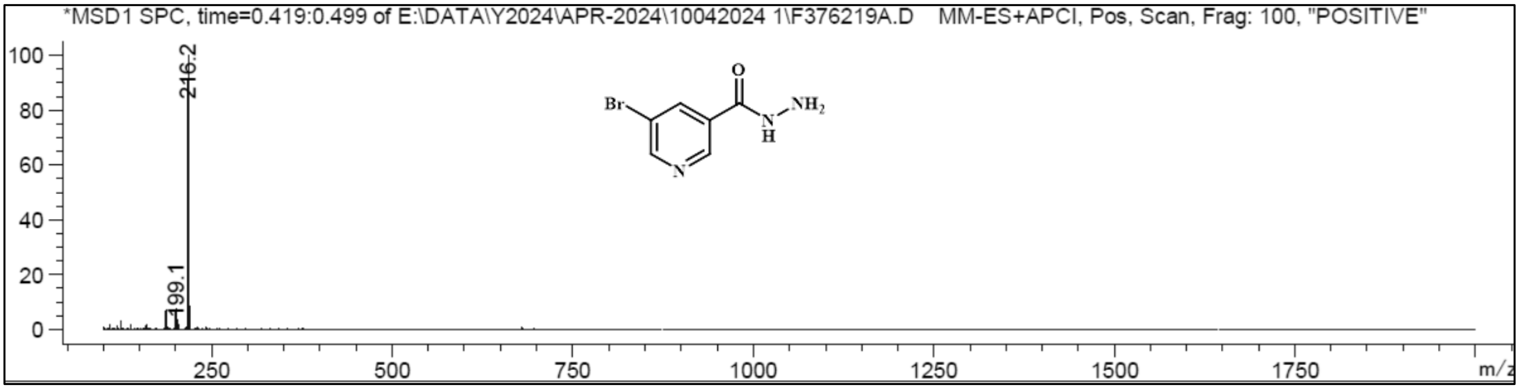

Figure S15: LCMS of compound **11**

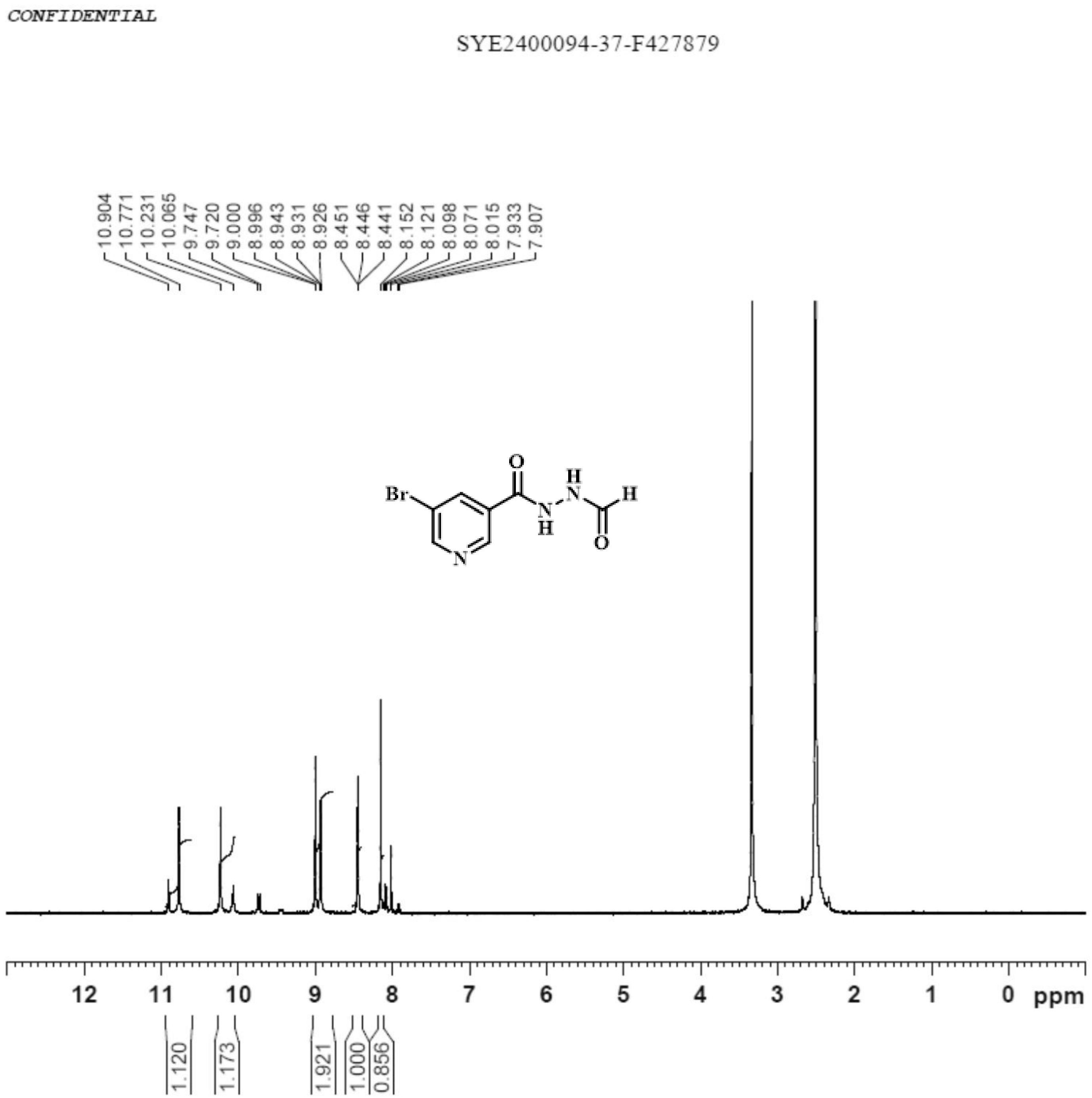

Figure S16: ^{1}H NMR (400 MHz, DMSO-d_6) of compound **12**

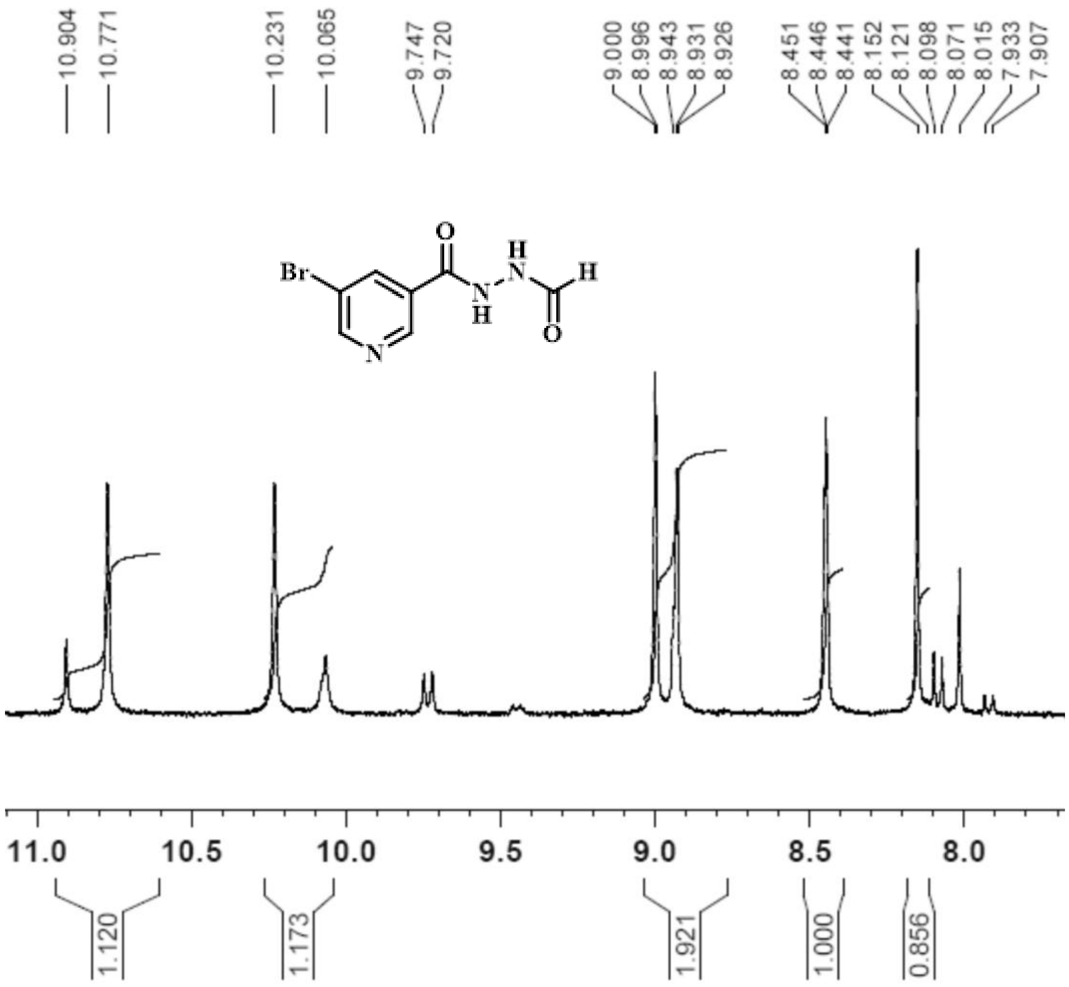

Figure S17: Expanded ^{1}H NMR (400 MHz, DMSO-d_6) of compound **12**

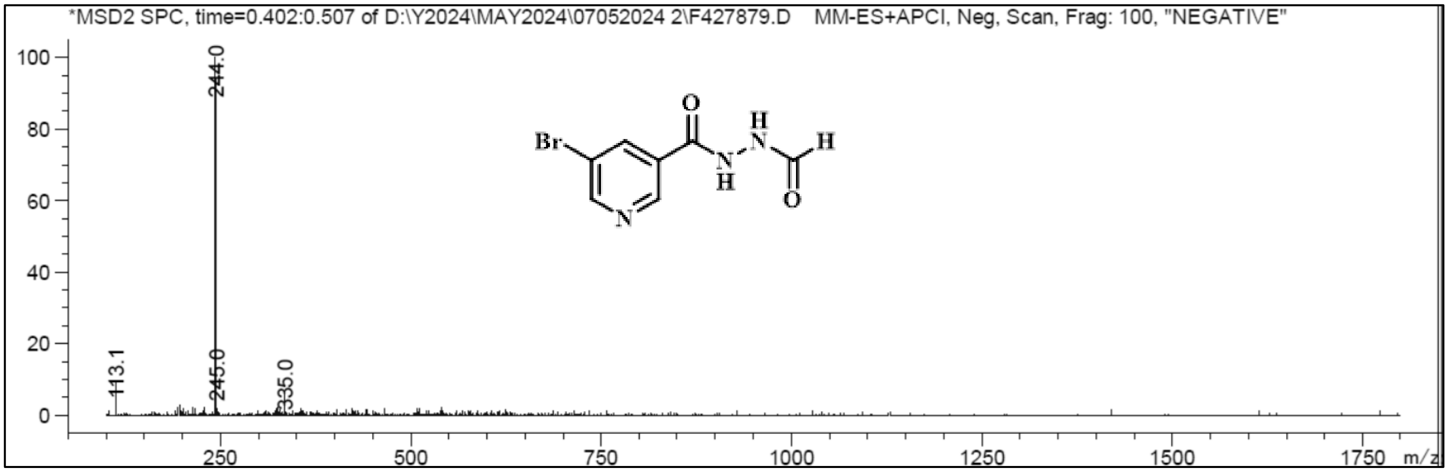

Figure S18: LCMS of compound **12**

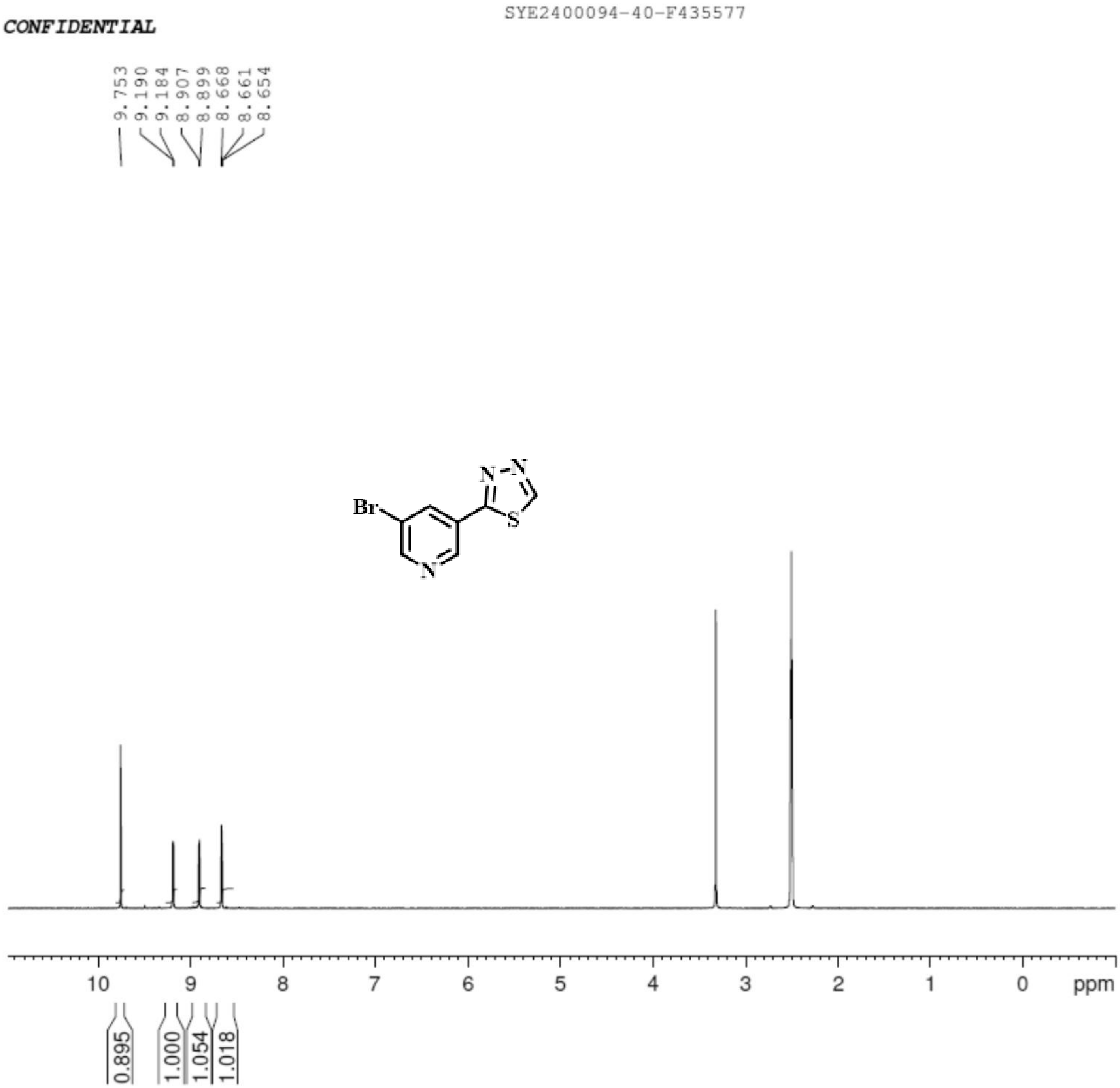

Figure S19: ^{1}H NMR (400 MHz, DMSO-d_6) of compound **13**

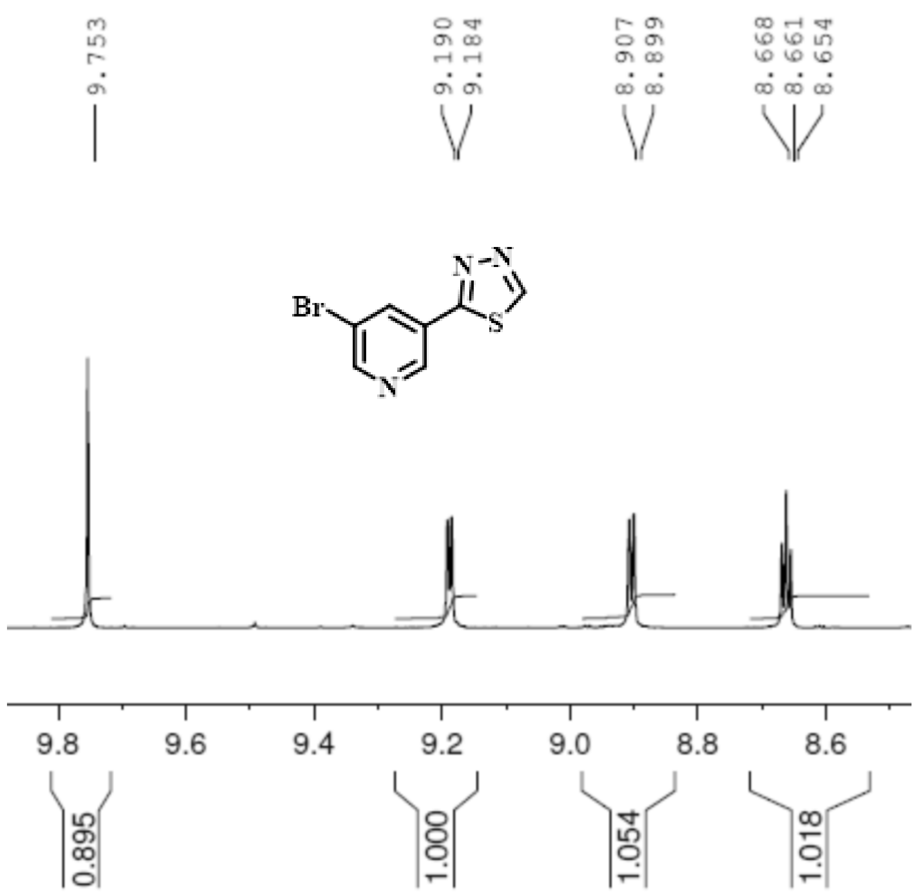

Figure S20: Expanded ^{1}H NMR (400 MHz, DMSO-d_6) of compound **13**

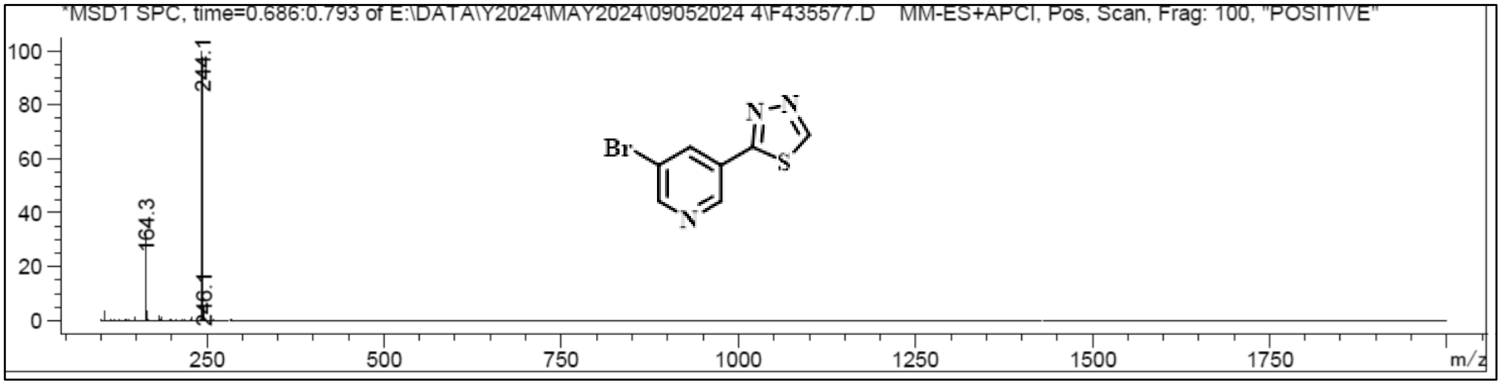

Figure S21: LCMS of compound **13**

CONFIDENTIAL

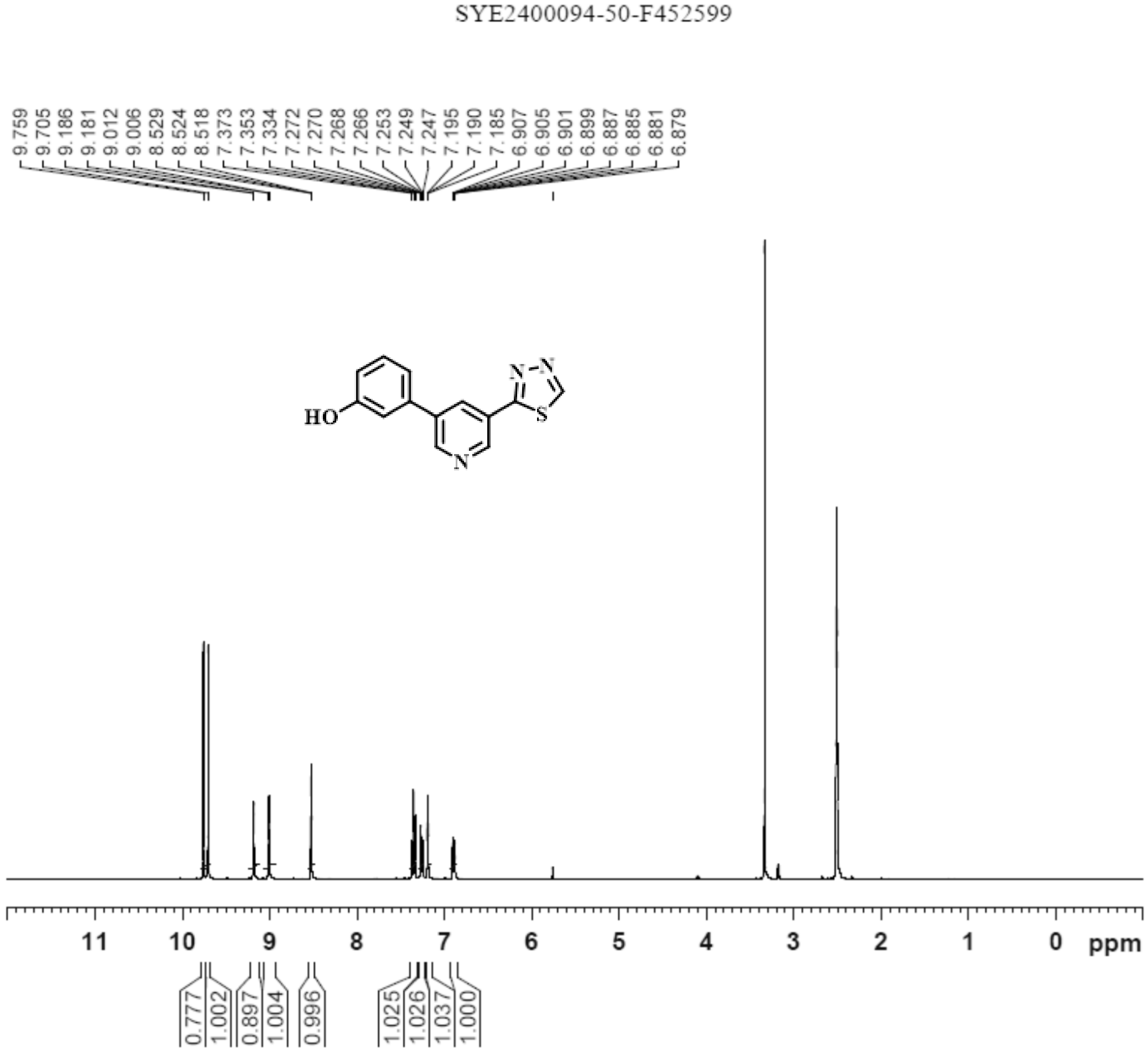

Figure S22: ^{1}H NMR (400 MHz, DMSO-d_6) of compound **14**

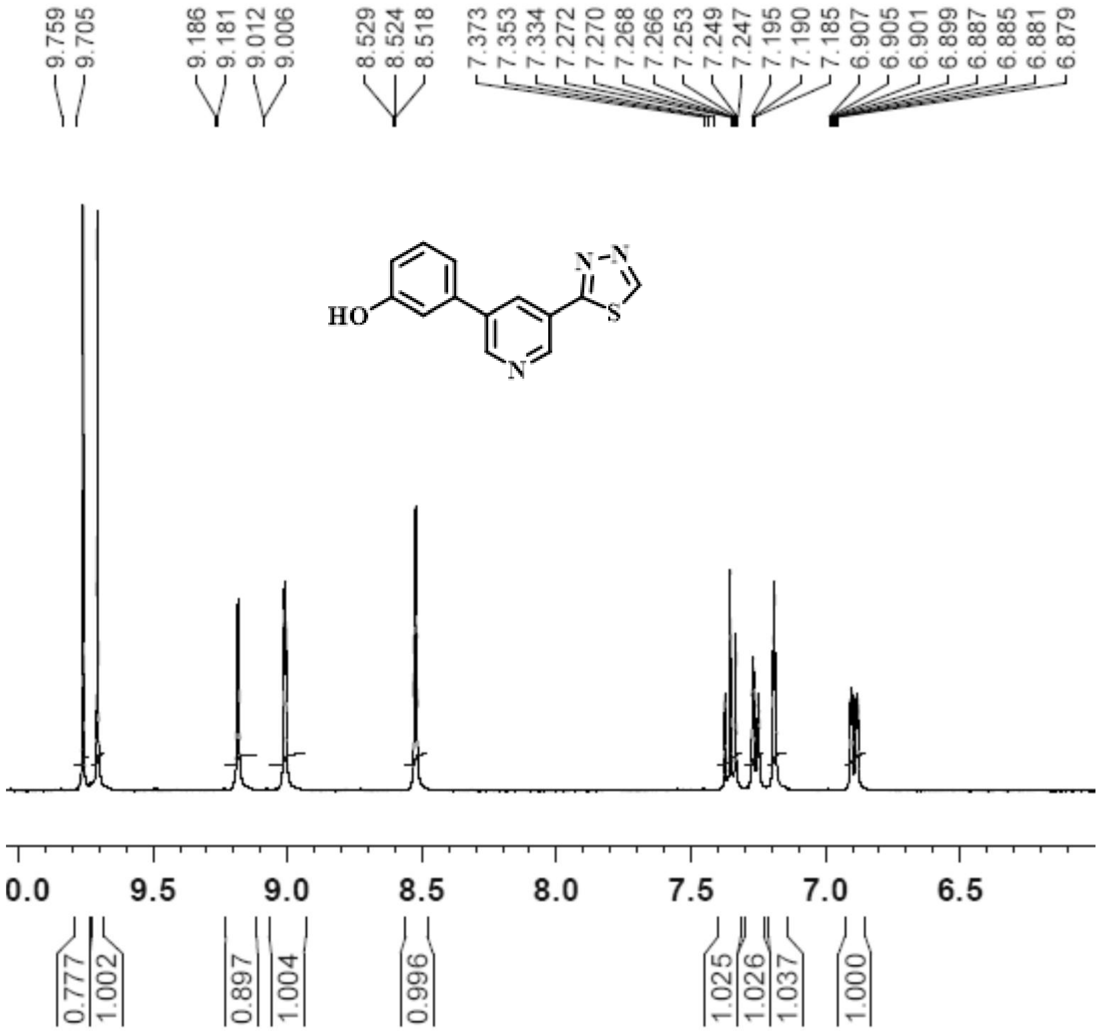

Figure S23: Expanded ^{1}H NMR (400 MHz, DMSO-d_6) of compound **14**

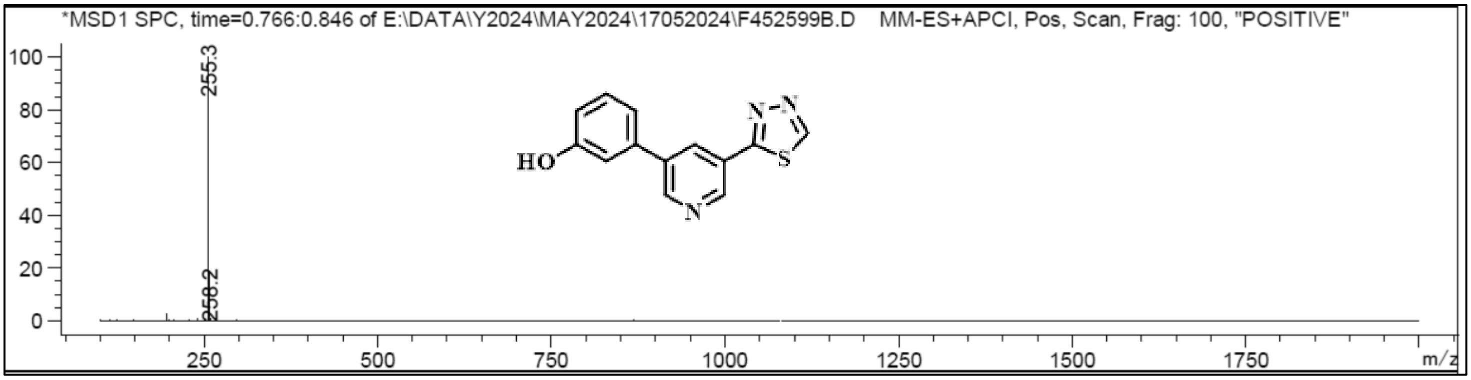

Figure S24: LCMS of compound **14**

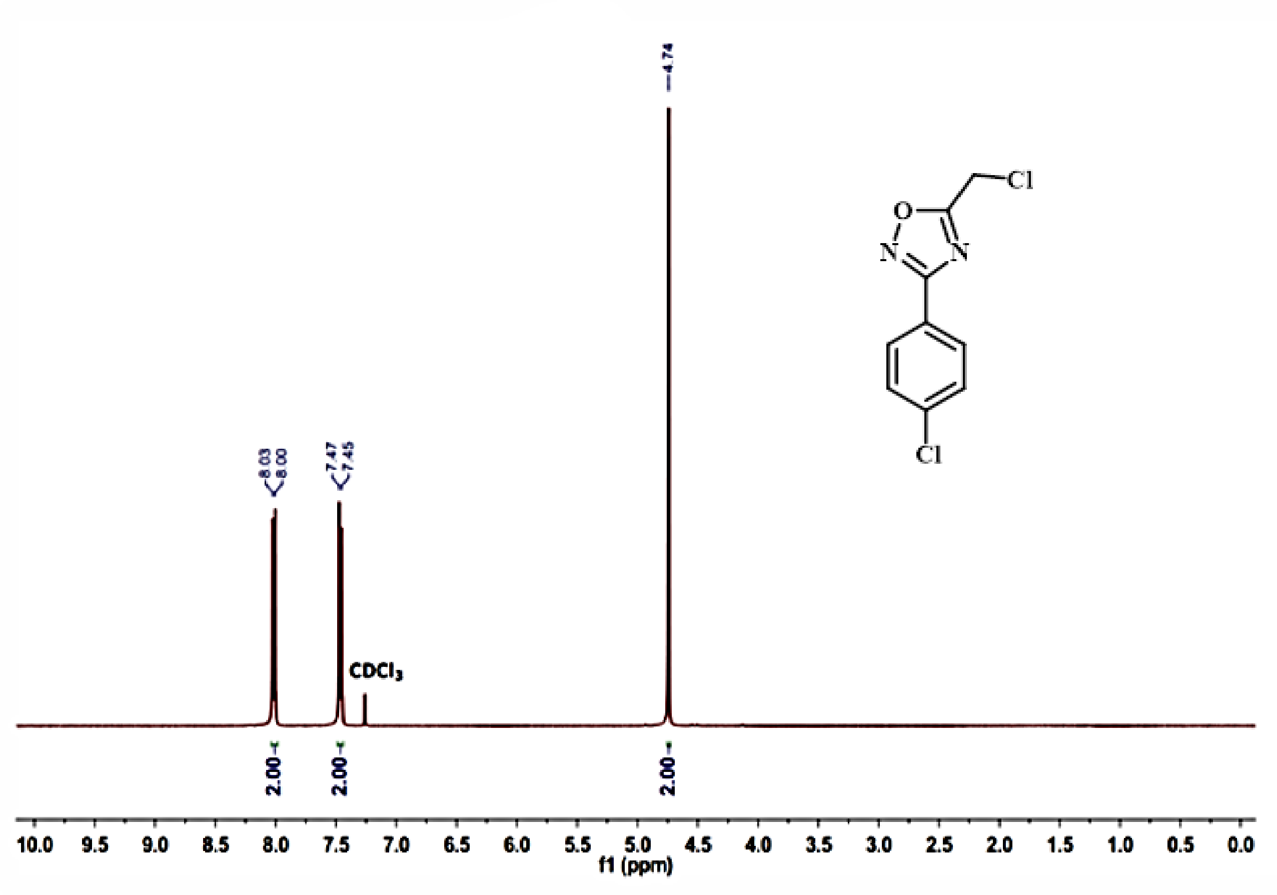

Figure S25: ^{1}H NMR spectrum (400 MHz, $CDCl_3$) of compound **19b**

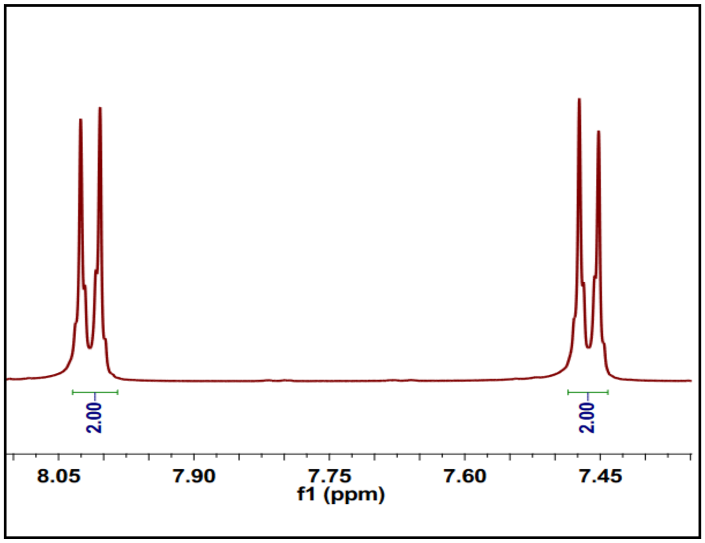

Figure S26: Expanded ^{1}H NMR spectrum (400 MHz, $CDCl_3$) of compound **19b**

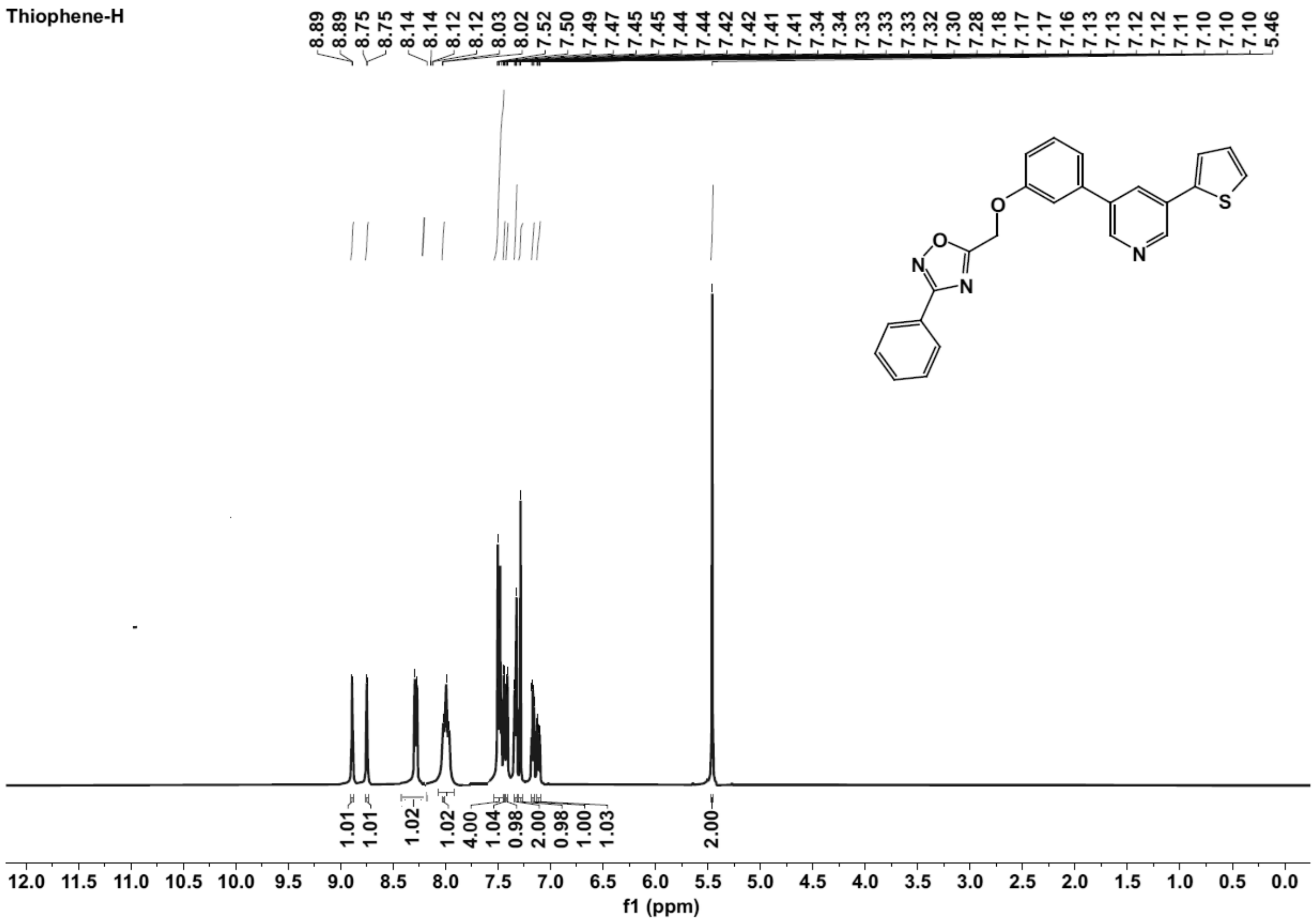

Figure S27: ^{1}H NMR spectrum (400 MHz, $CDCl_3$) of compound **20a**

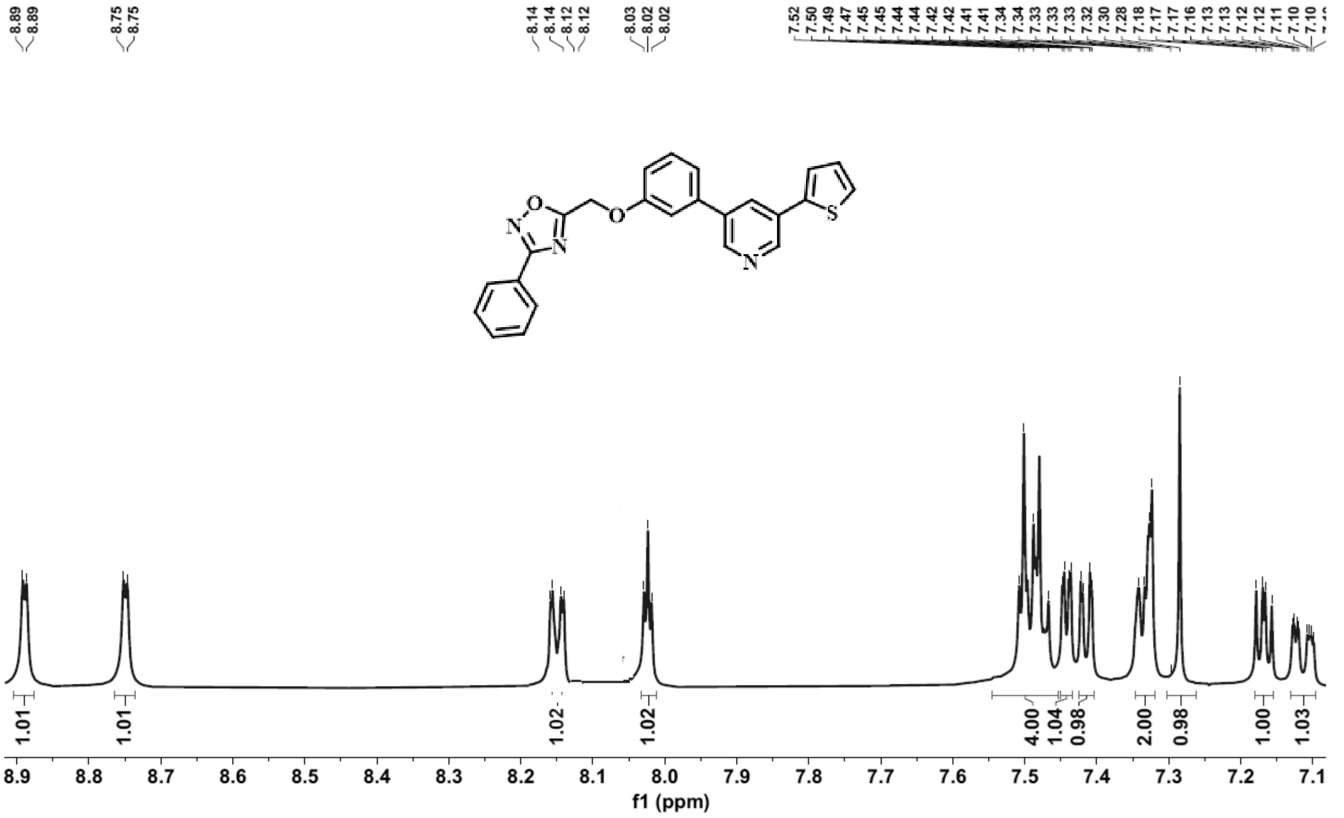

Figure S28: Expanded ^{1}H NMR spectrum (400 MHz, $CDCl_3$) of compound **20a**

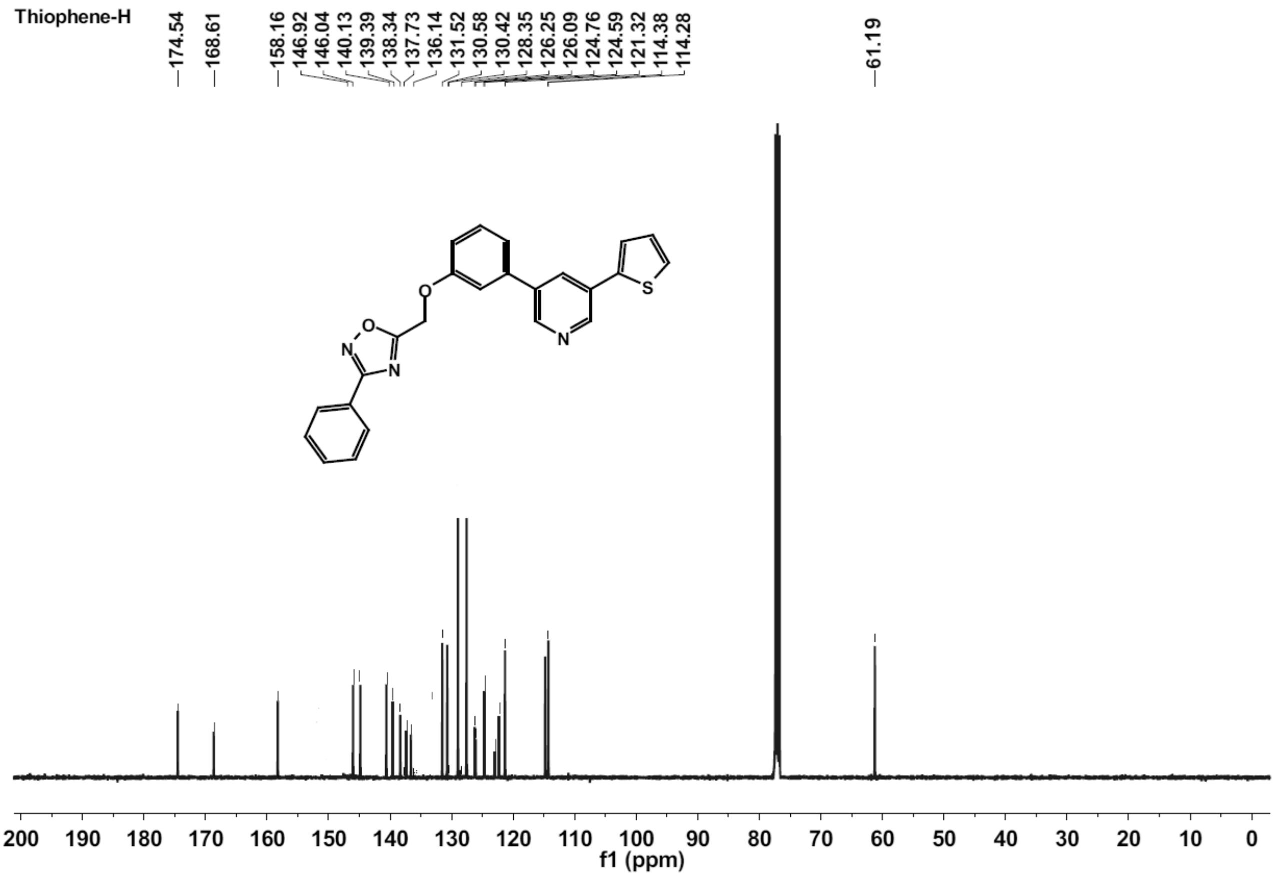

Figure S29: ^{13}C NMR spectrum (400 MHz, $CDCl_3$) of compound **20a**

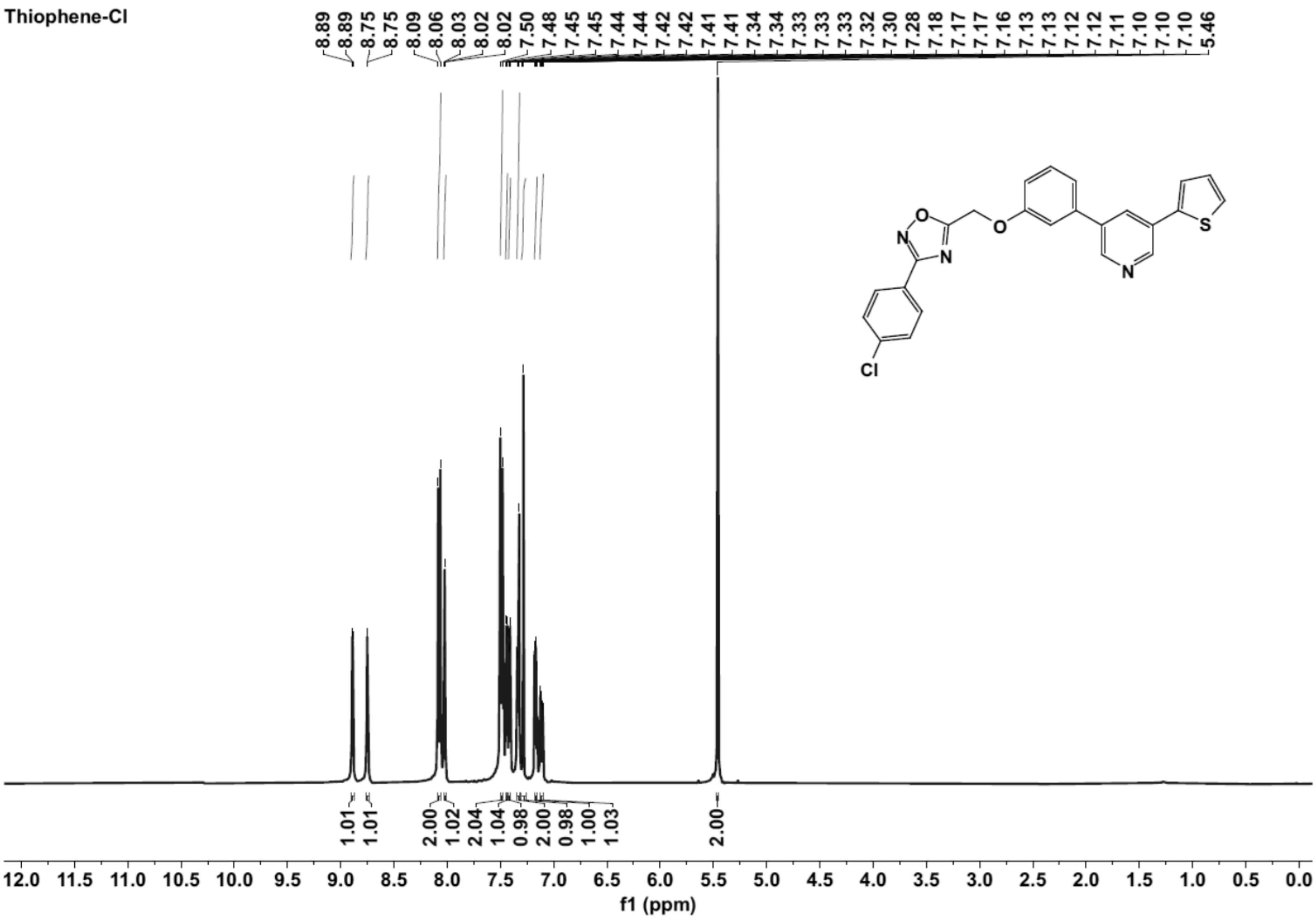

Figure S30: ^{1}H NMR spectrum (400 MHz, $CDCl_3$) of compound **20b**

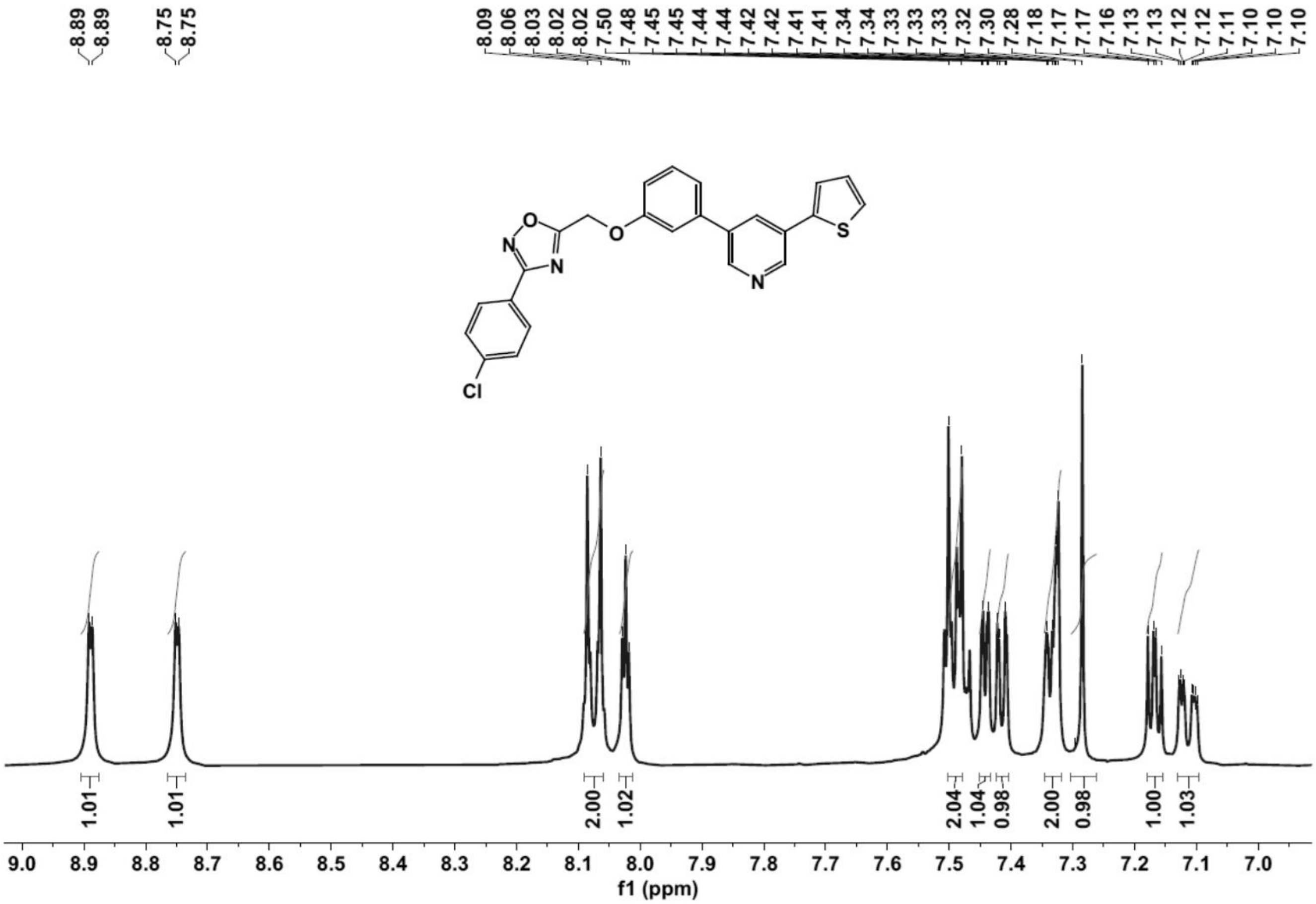

Figure S31: Expanded ^{1}H NMR spectrum (400 MHz, $CDCl_3$) of compound **20b**

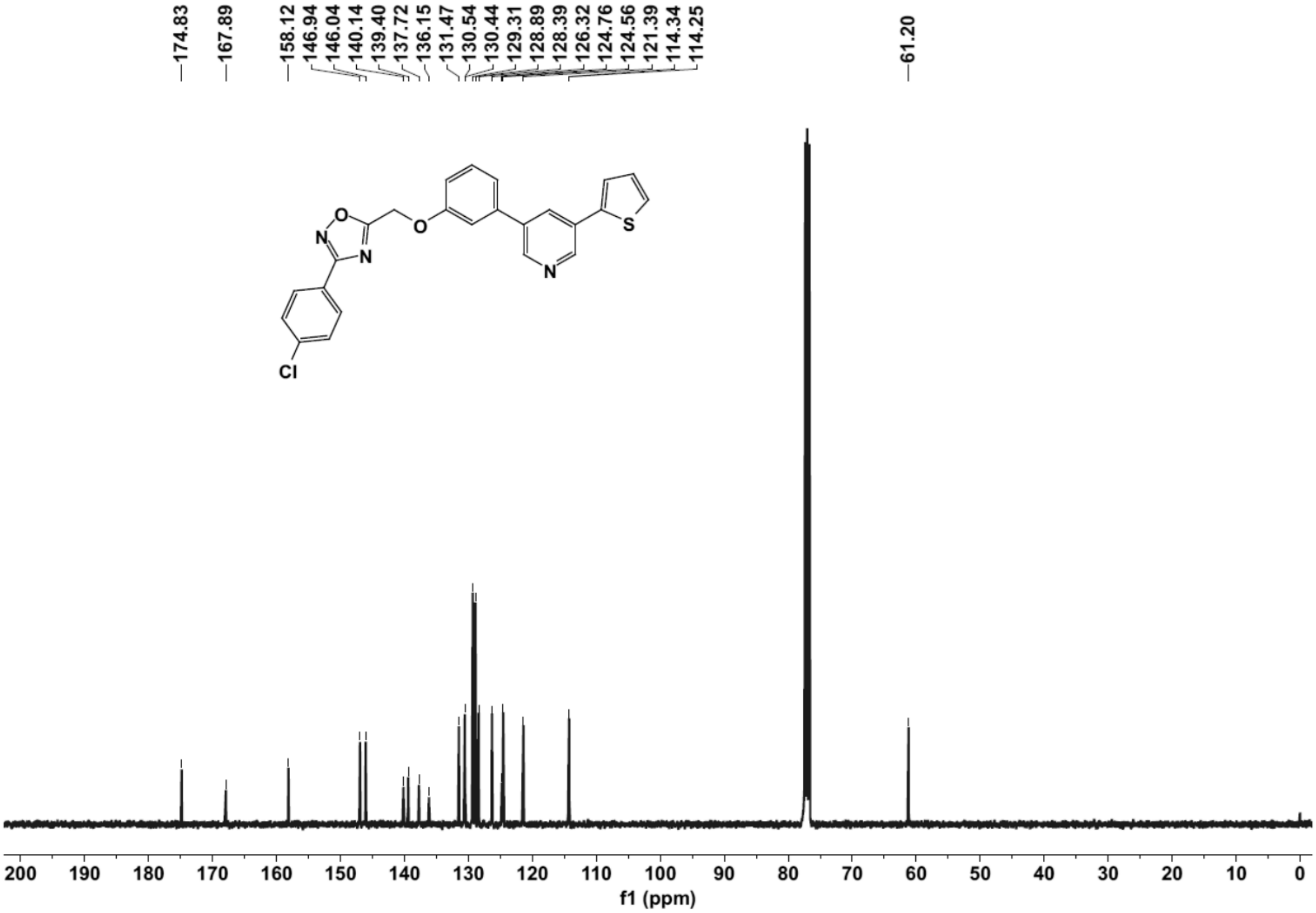

Figure S32: ^{13}C NMR spectrum (400 MHz, $CDCl_3$) of compound **20b**

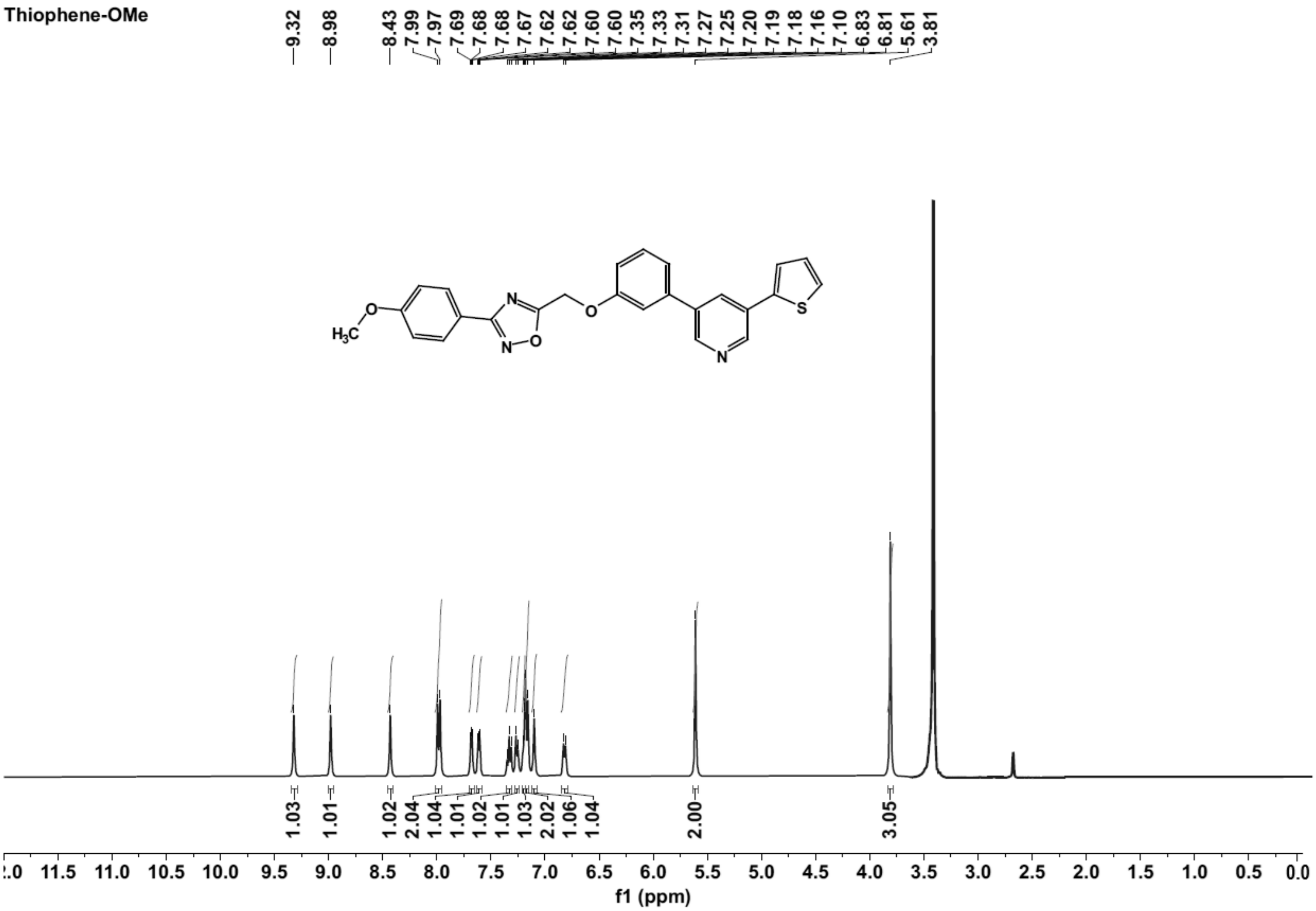

Figure S33: ^{1}H NMR (500 MHz, DMSO-d_6) of compound **20c**

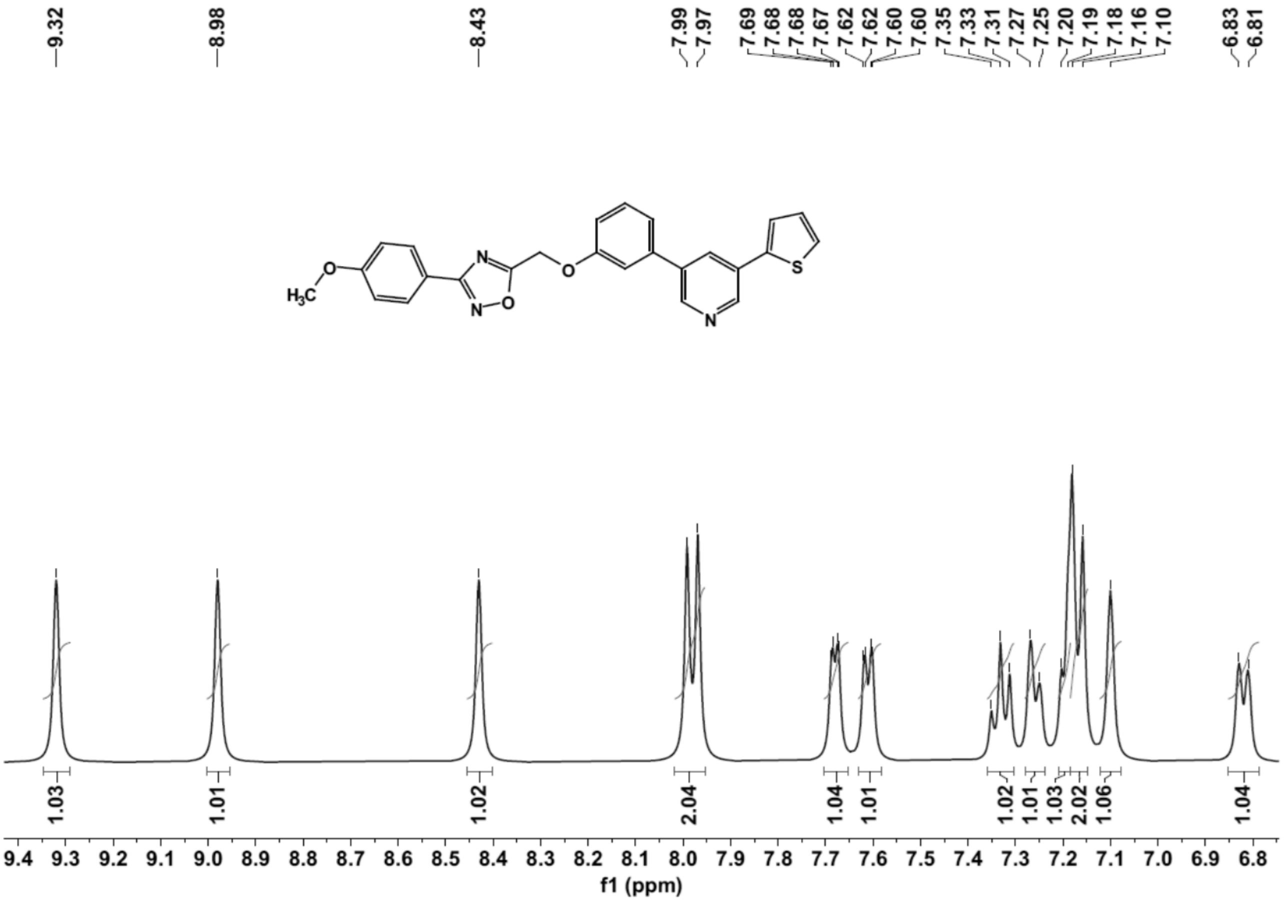

Figure S34: Expanded ^{1}H NMR (500 MHz, DMSO-d_6) of compound **20c**

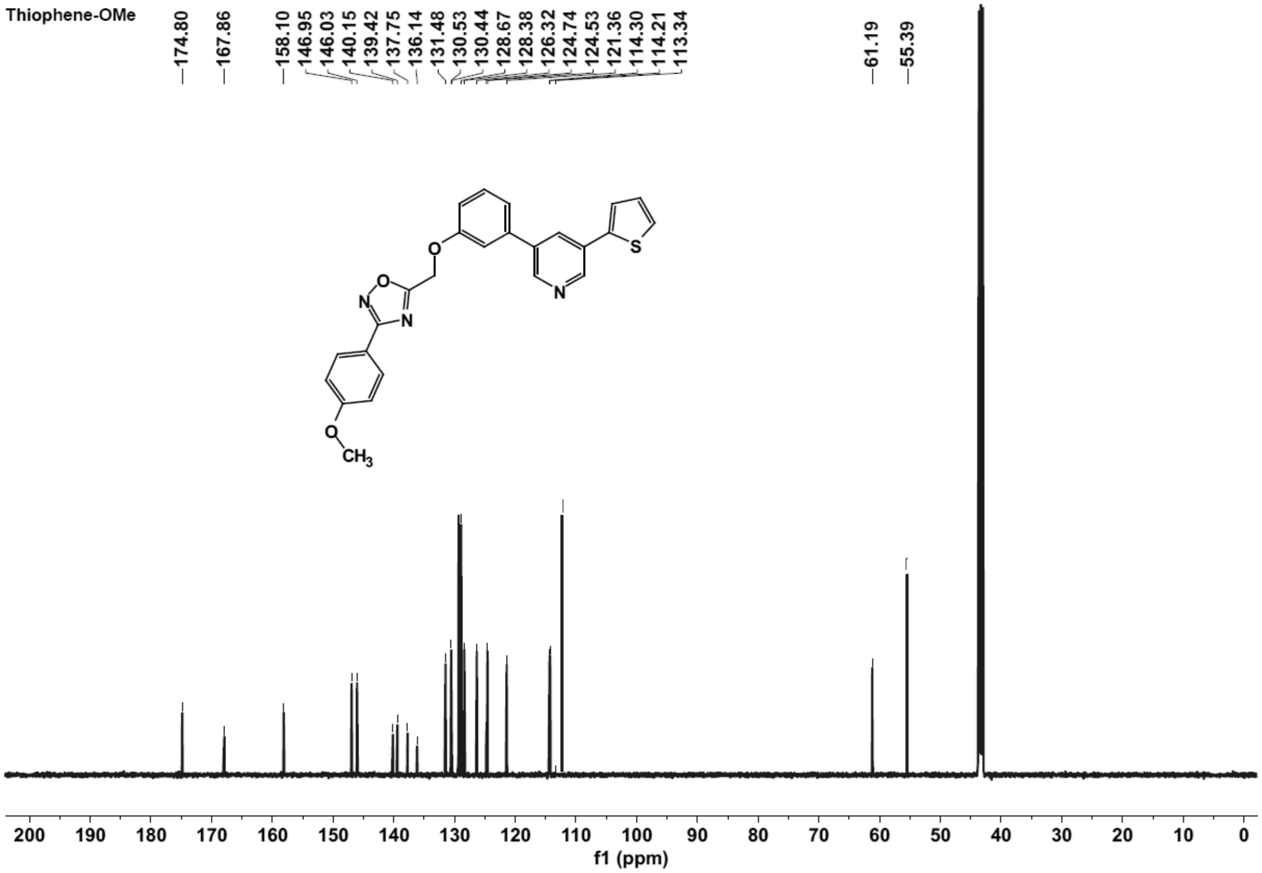

Figure S35: ^{13}C NMR (500 MHz, DMSO-d_6) of compound **20c**

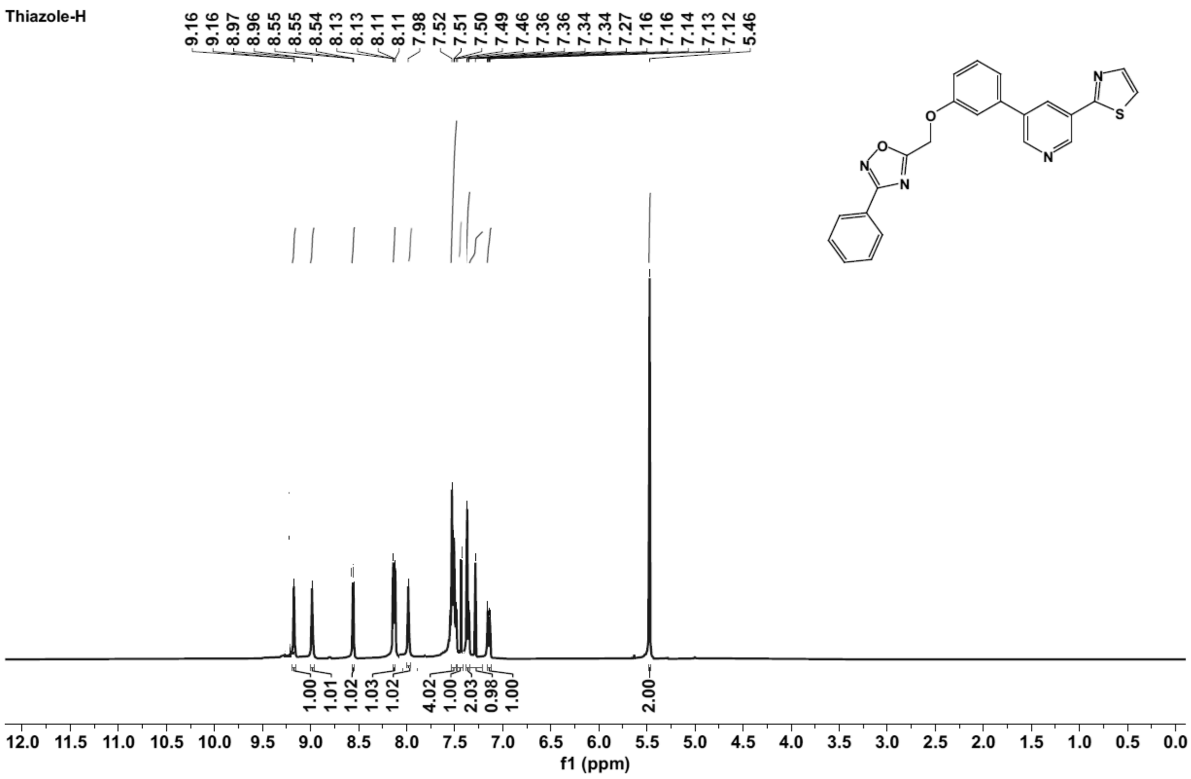

Figure S36: ^{1}H NMR spectrum (400 MHz, $CDCl_3$) of compound **21a**

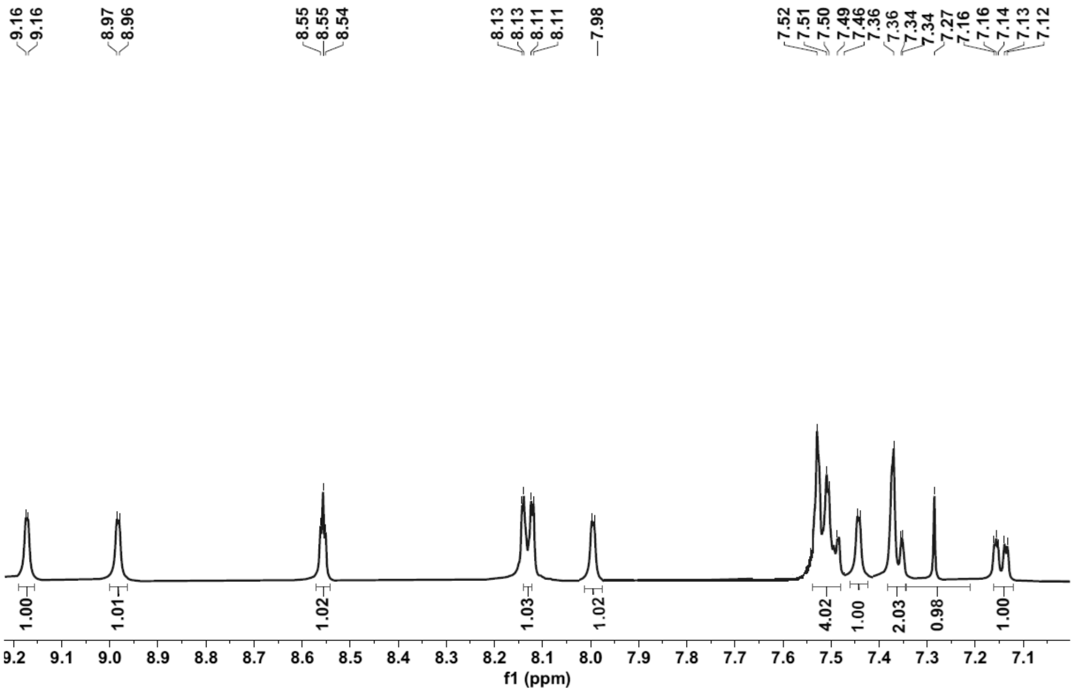

Figure S37: Expanded ^{1}H NMR spectrum (400 MHz, $CDCl_3$) of compound **21a**

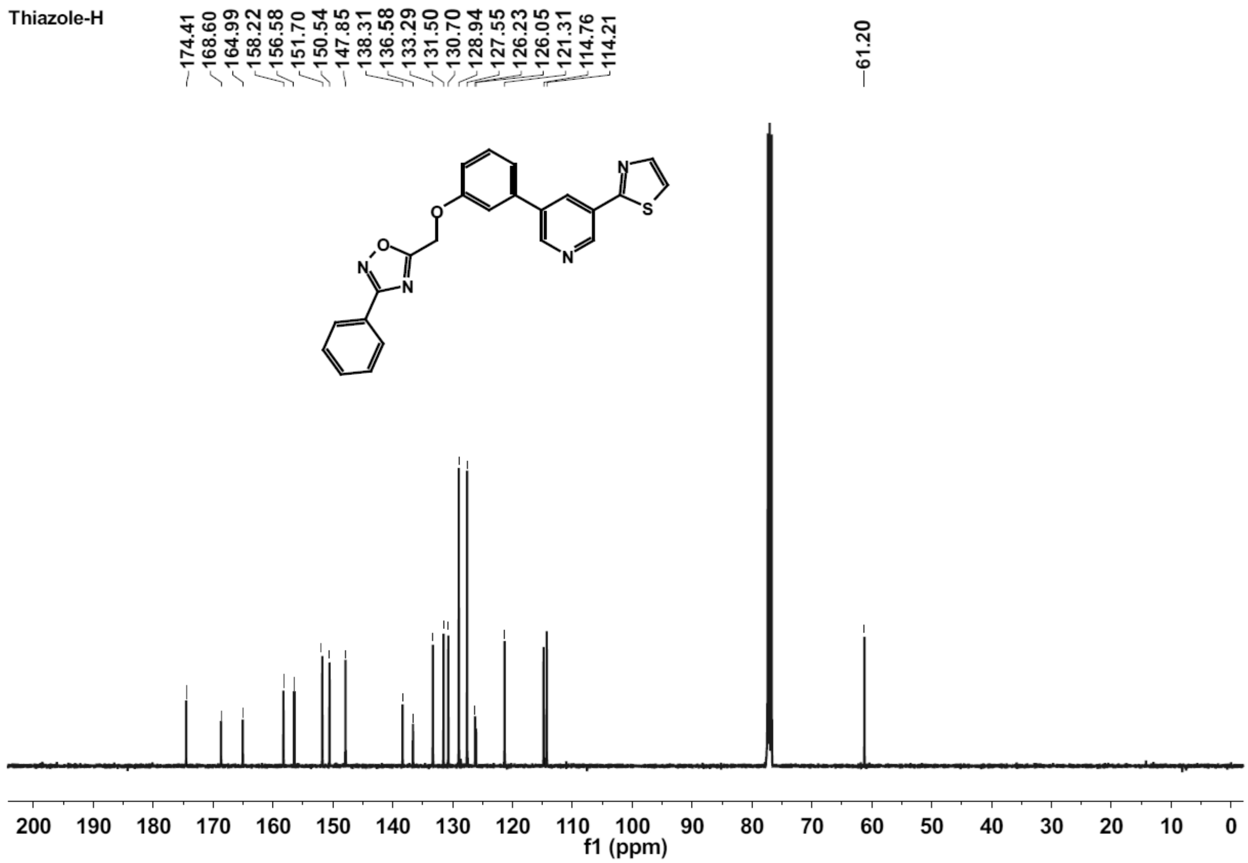

Figure S38: ^{13}C NMR spectrum (400 MHz, $CDCl_3$) of compound **21a**

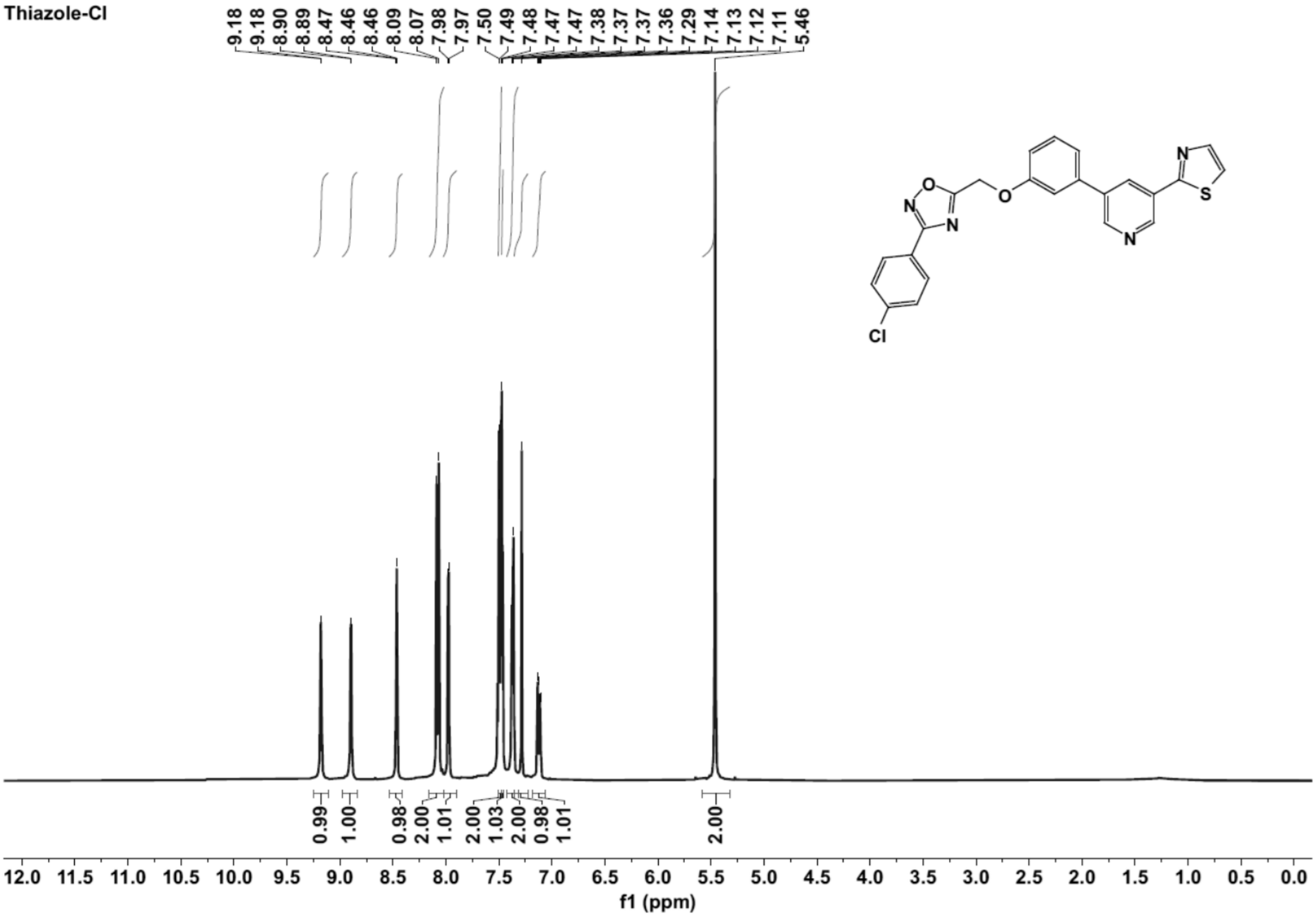

Figure S39: ^{1}H NMR spectrum (400 MHz, $CDCl_3$) of compound **21b**

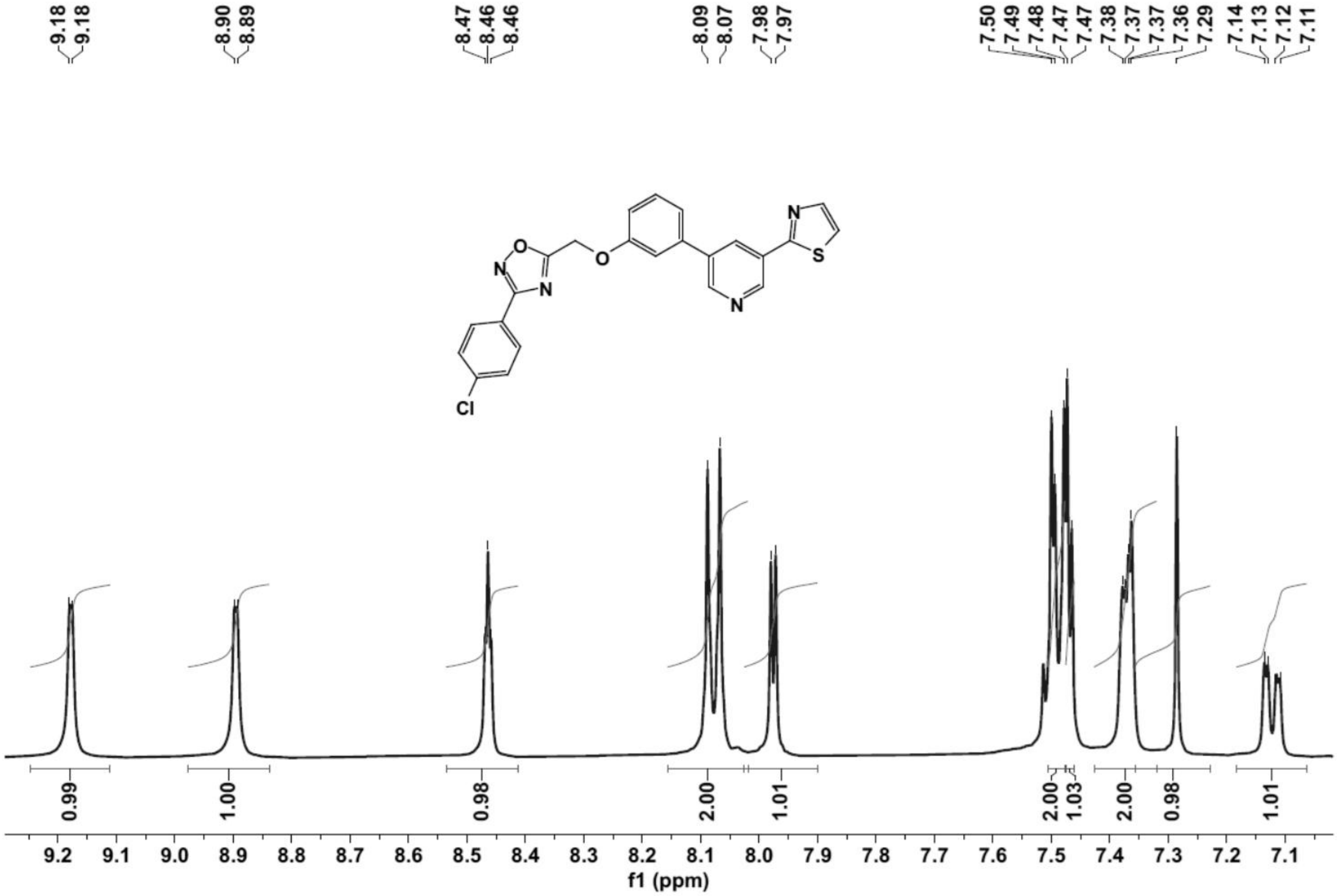

Figure S40: Expanded ^{1}H NMR spectrum (400 MHz, $CDCl_3$) of compound **21b**

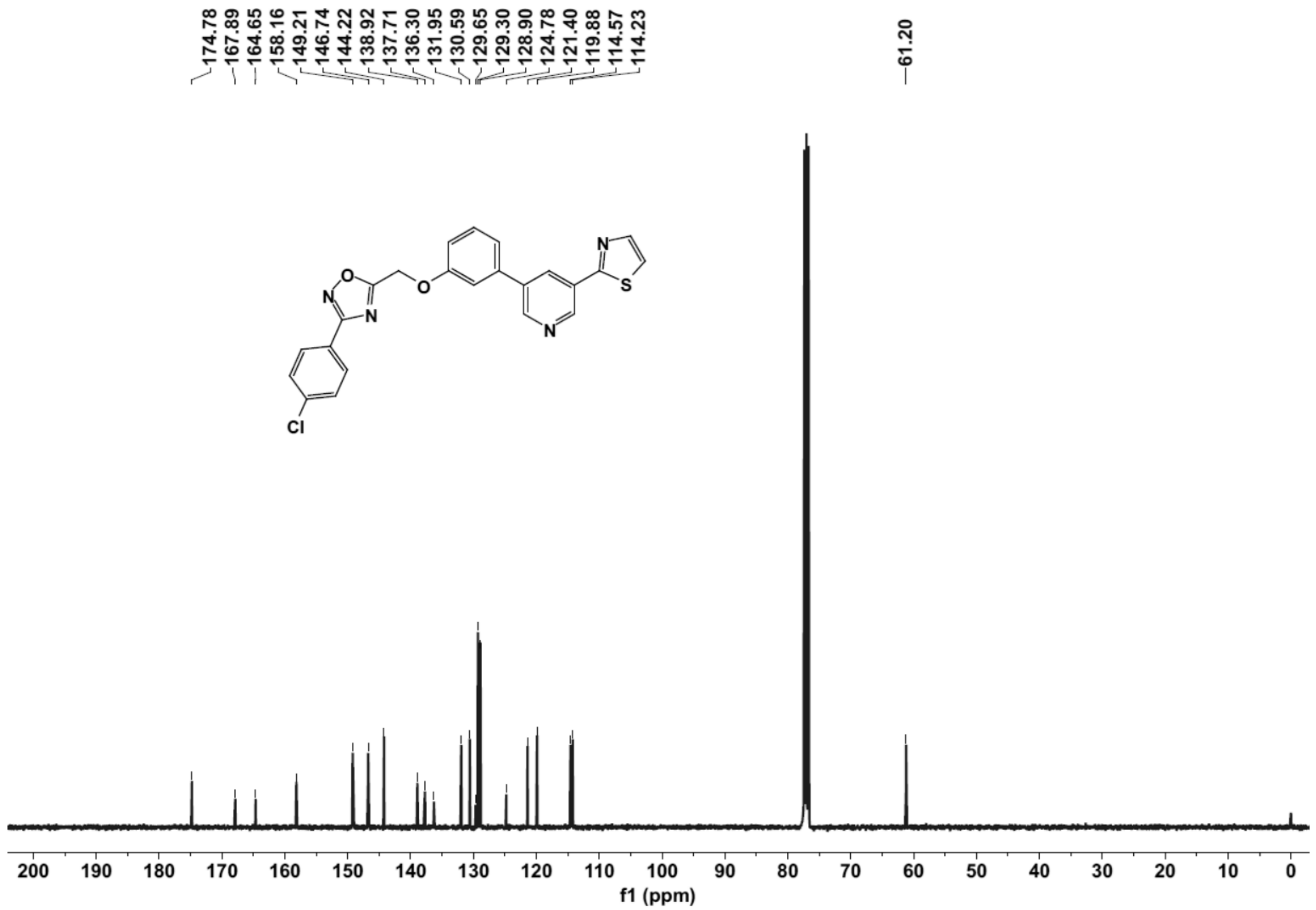

Figure S41: ^{13}C NMR spectrum (400 MHz, $CDCl_3$) of compound **21b**

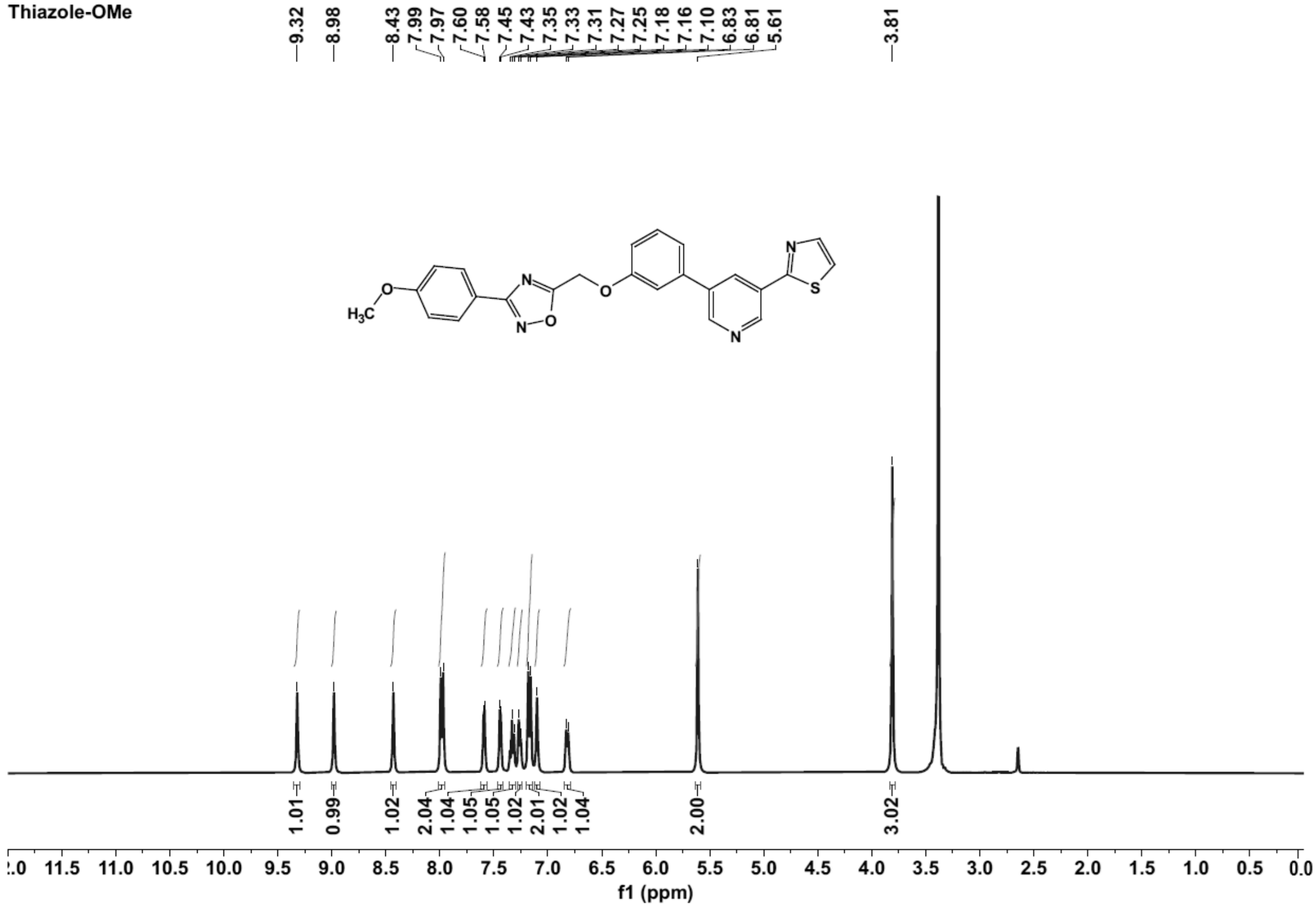

Figure S42: ^{1}H NMR (500 MHz, DMSO-d_6) of compound **21c**

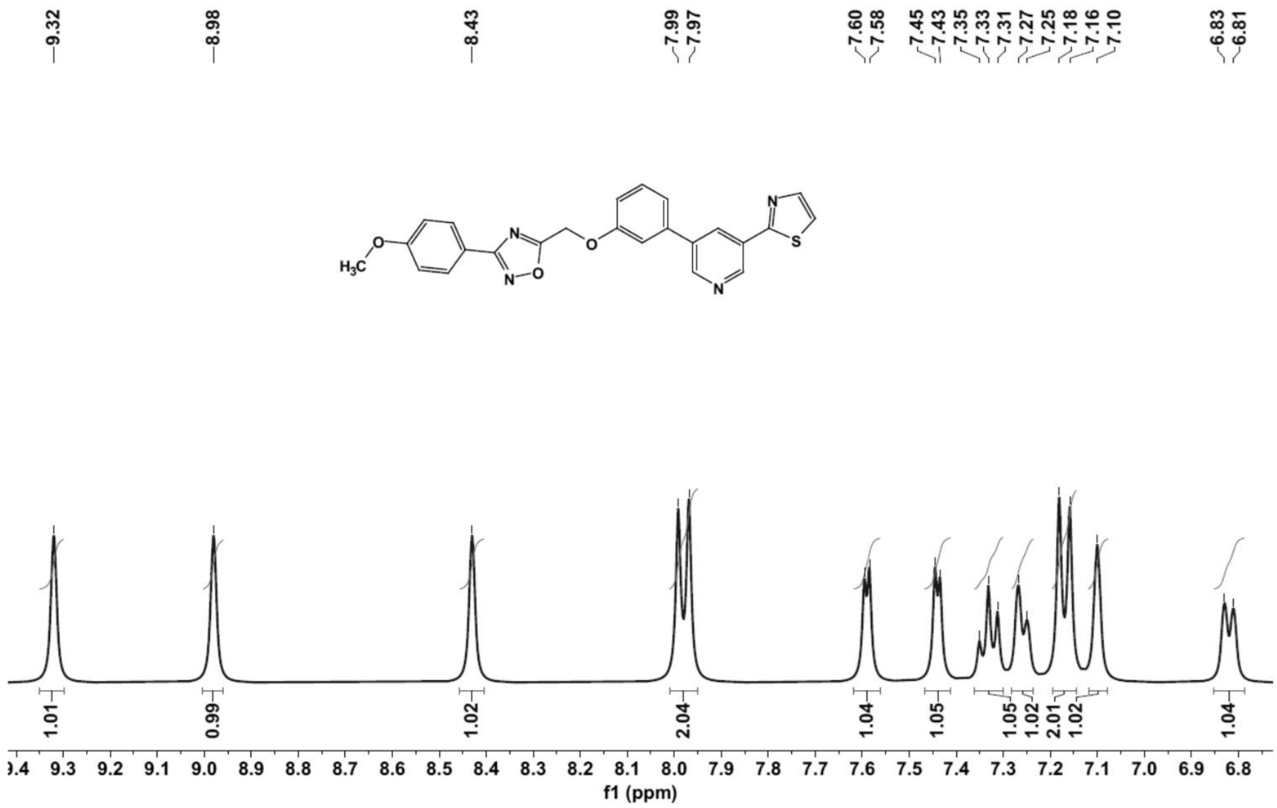

Figure S43: Expanded ^{1}H NMR (500 MHz, DMSO-d_6) of compound **21c**

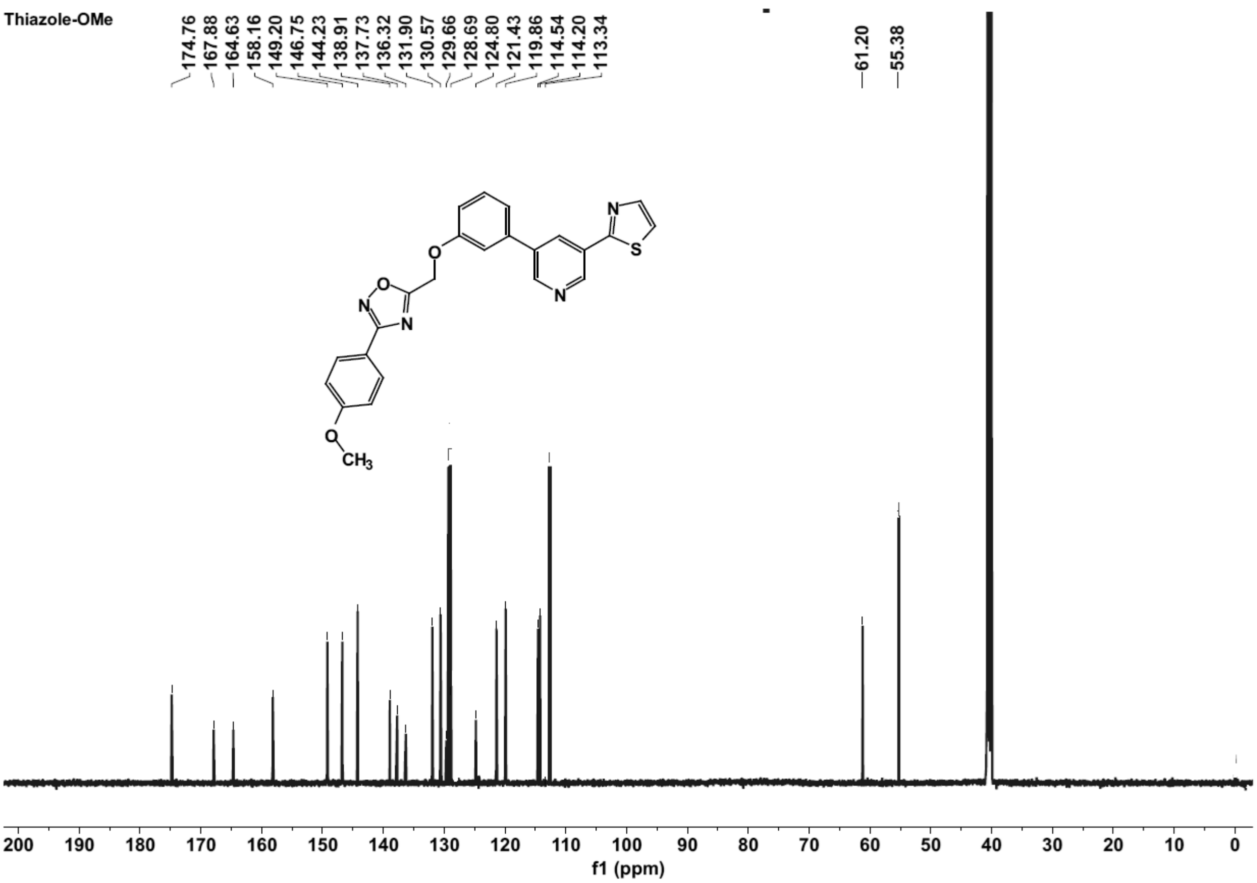

Figure S44: ^{13}C NMR (500 MHz, DMSO-d_6) of compound **21c**

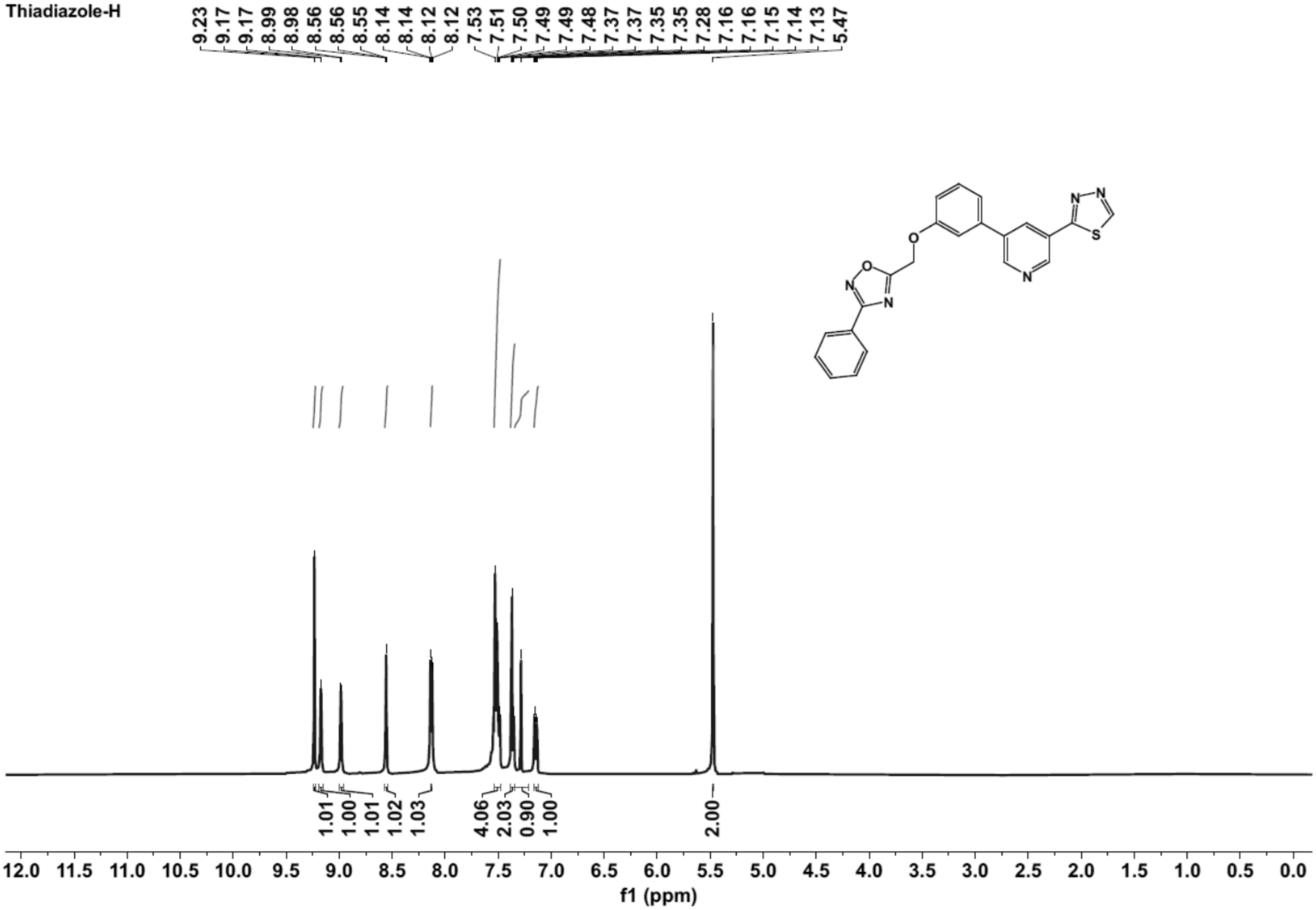

Figure S45: ^{1}H NMR spectrum (400 MHz, $CDCl_3$) of compound **22a**

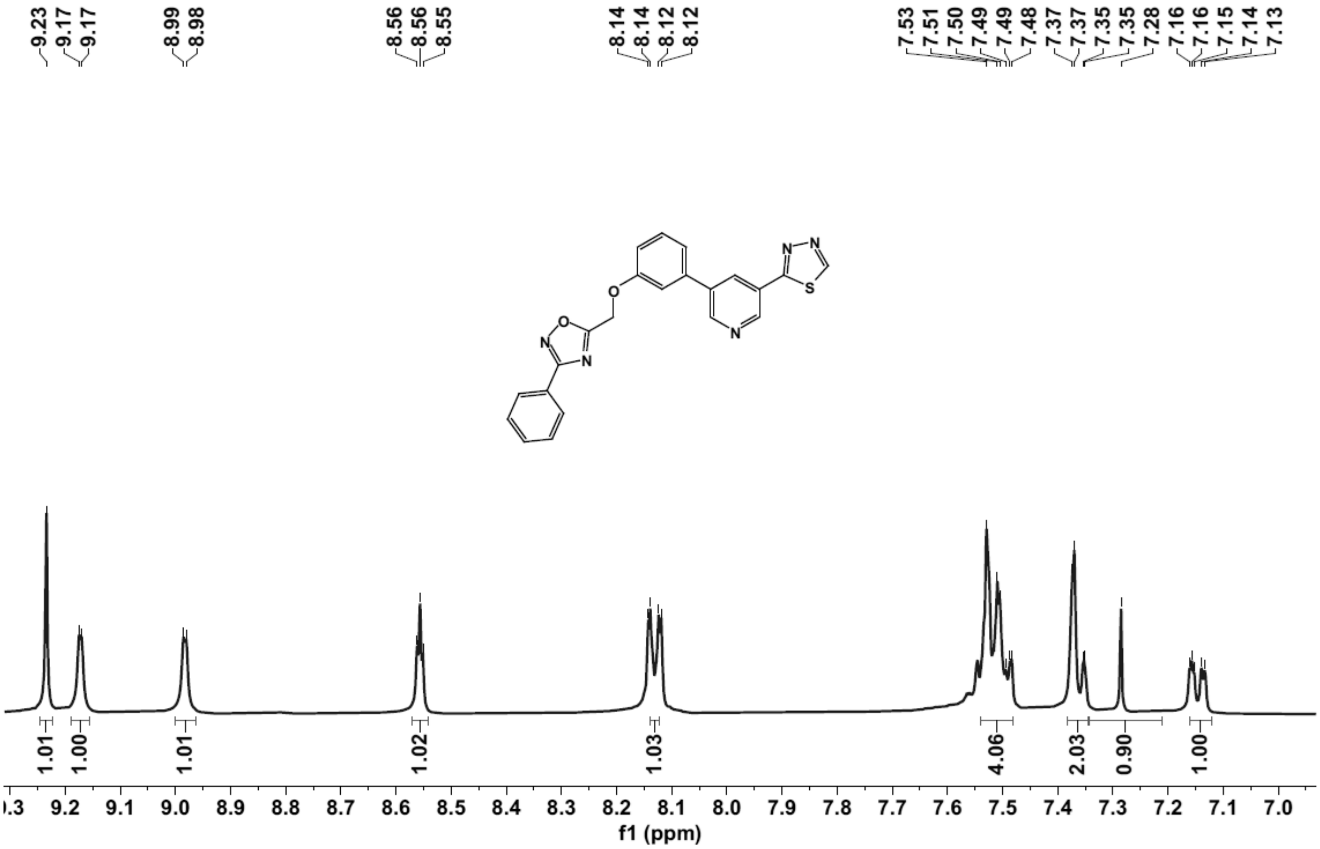

Figure S46: Expanded ^{1}H NMR spectrum (400 MHz, $CDCl_3$) of compound **22a**

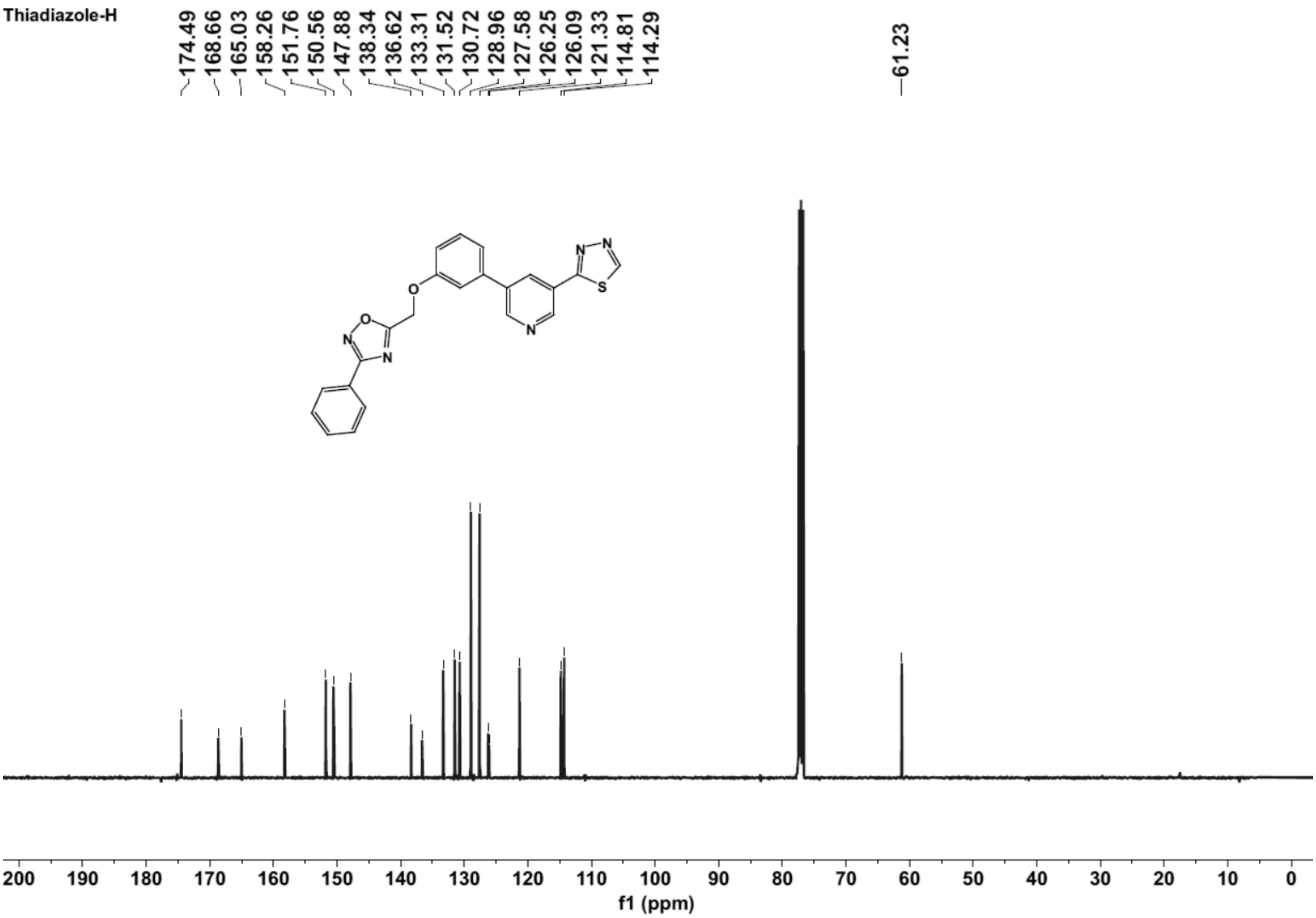

Figure S47: ^{13}C NMR spectrum (400 MHz, $CDCl_3$) of compound **22a**

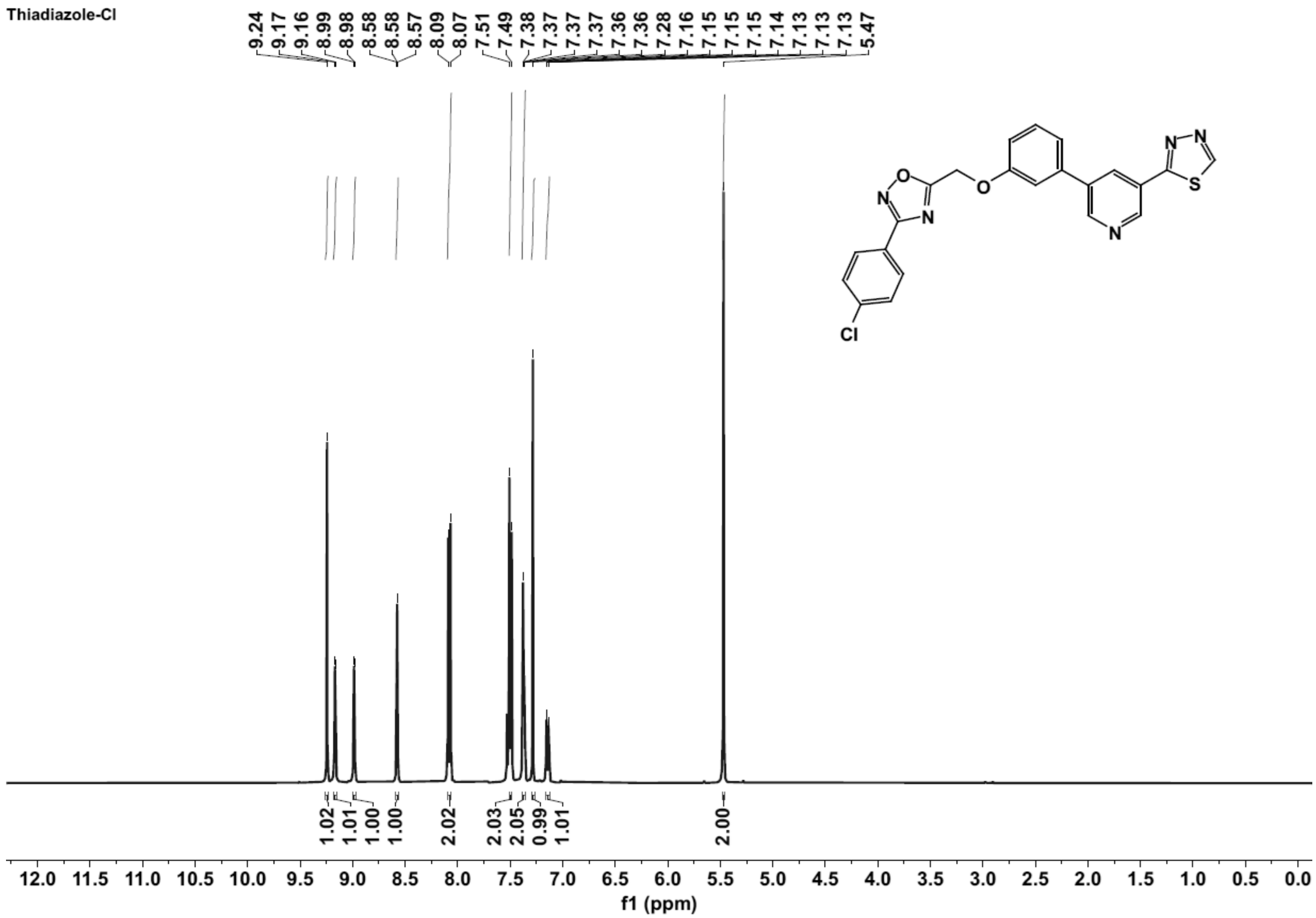

Figure S48: ^{1}H NMR spectrum (400 MHz, $CDCl_3$) of compound **22b**

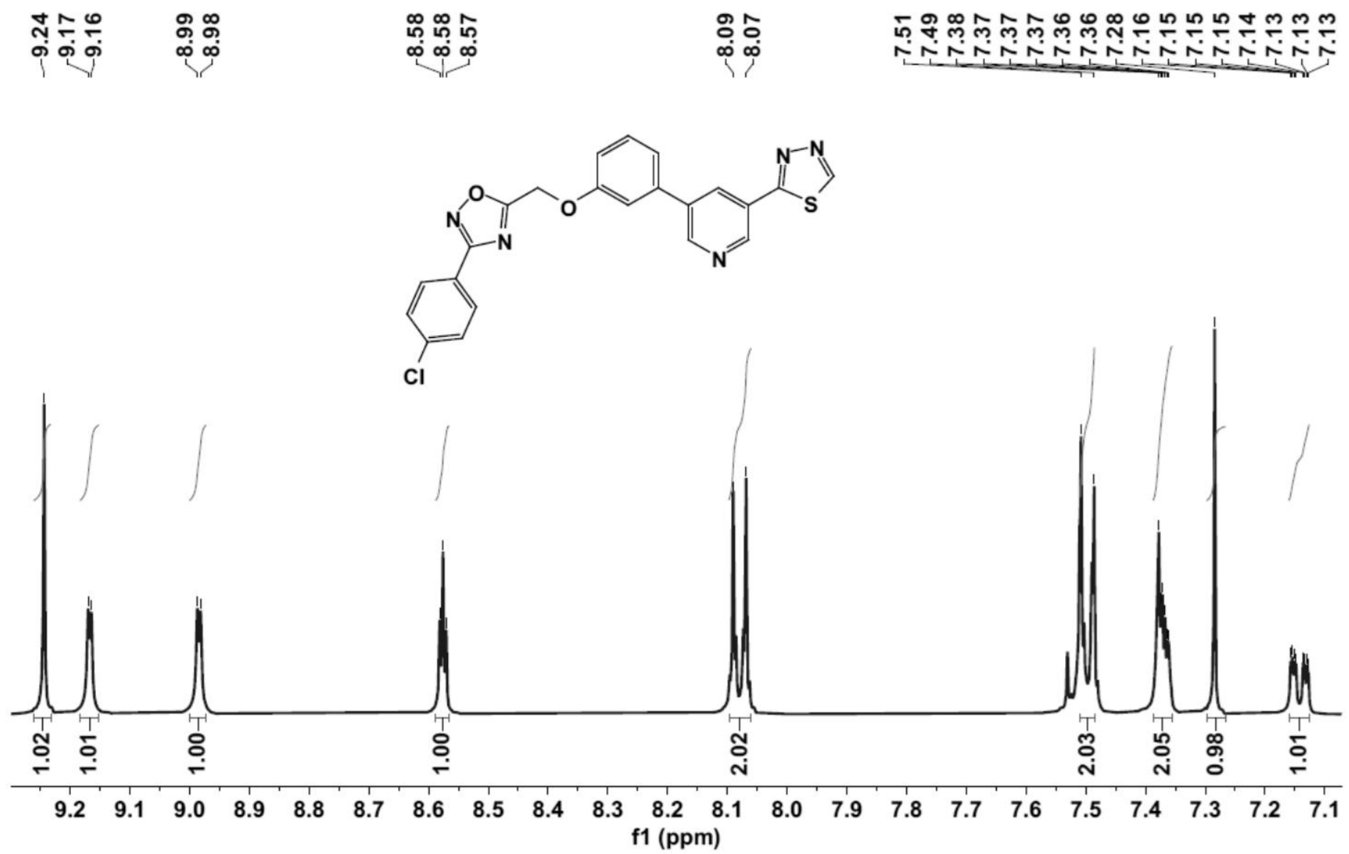

Figure S49: Expanded ^{1}H NMR spectrum (400 MHz, $CDCl_3$) of compound **22b**

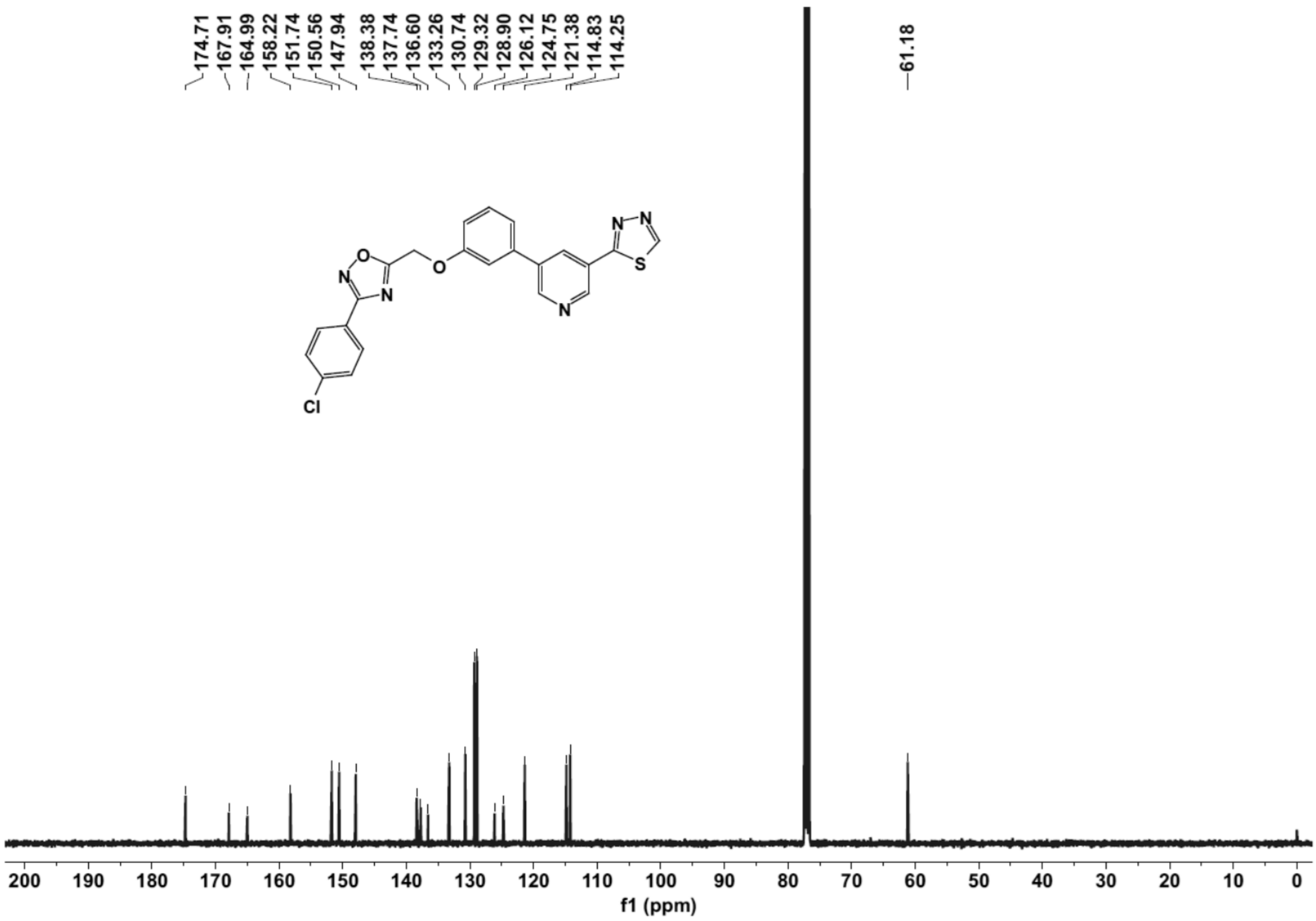

Figure S50: ^{13}C NMR spectrum (400 MHz, $CDCl_3$) of compound **22b**

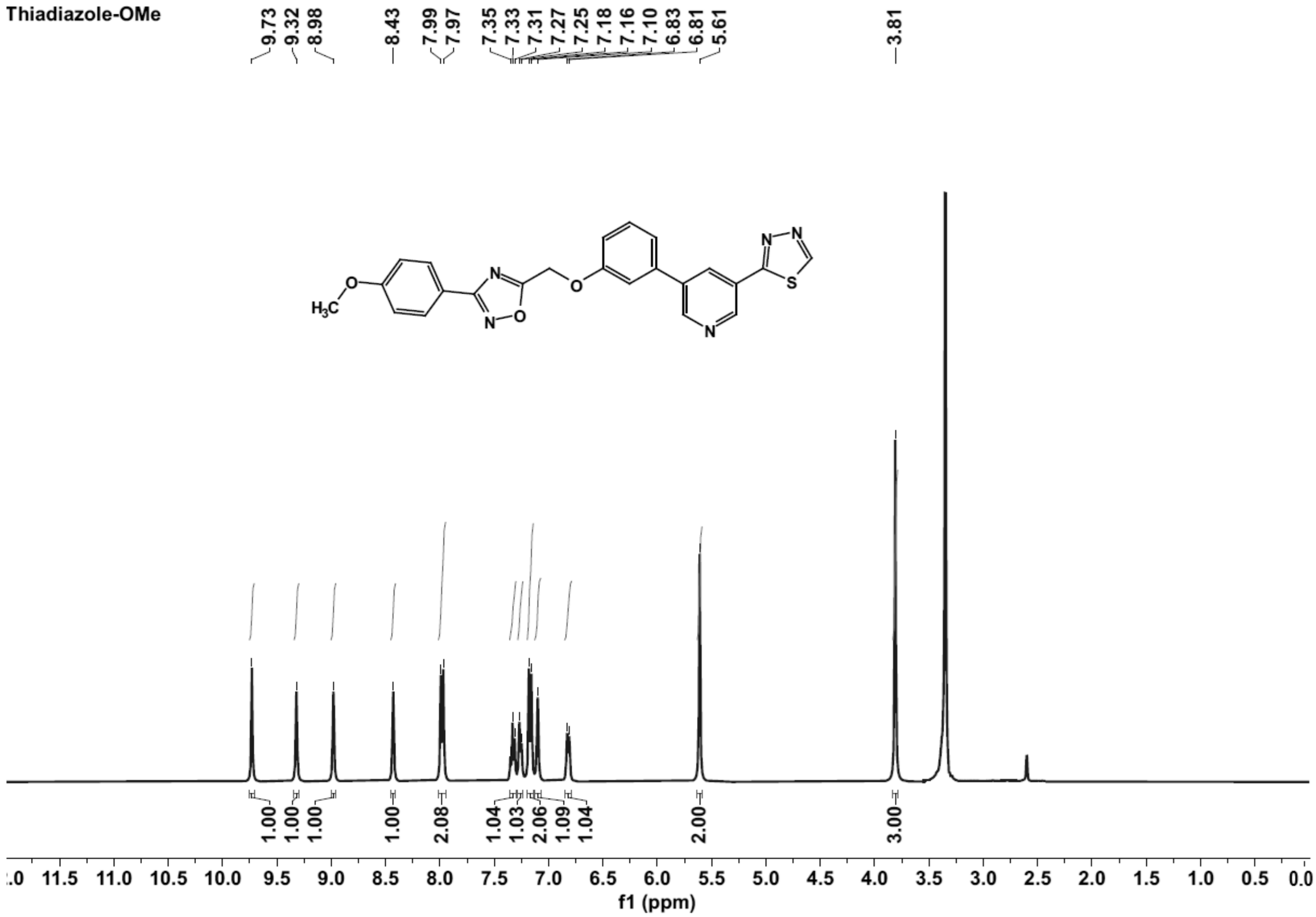

Figure S51: ^{1}H NMR (500 MHz, DMSO-d_6) of compound **22c**

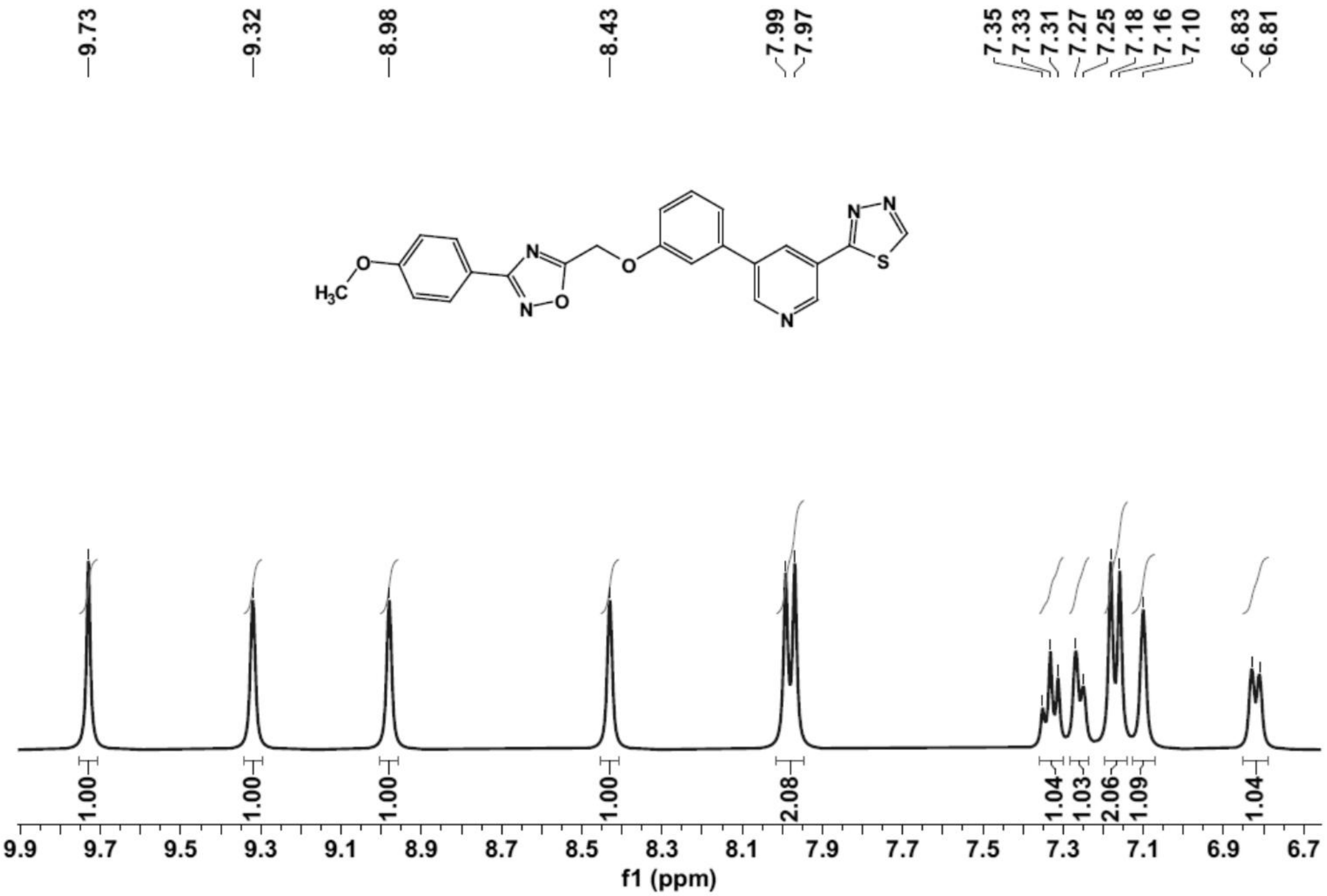

Figure S52: Expanded ^{1}H NMR (500 MHz, DMSO-d_6) of compound **22c**

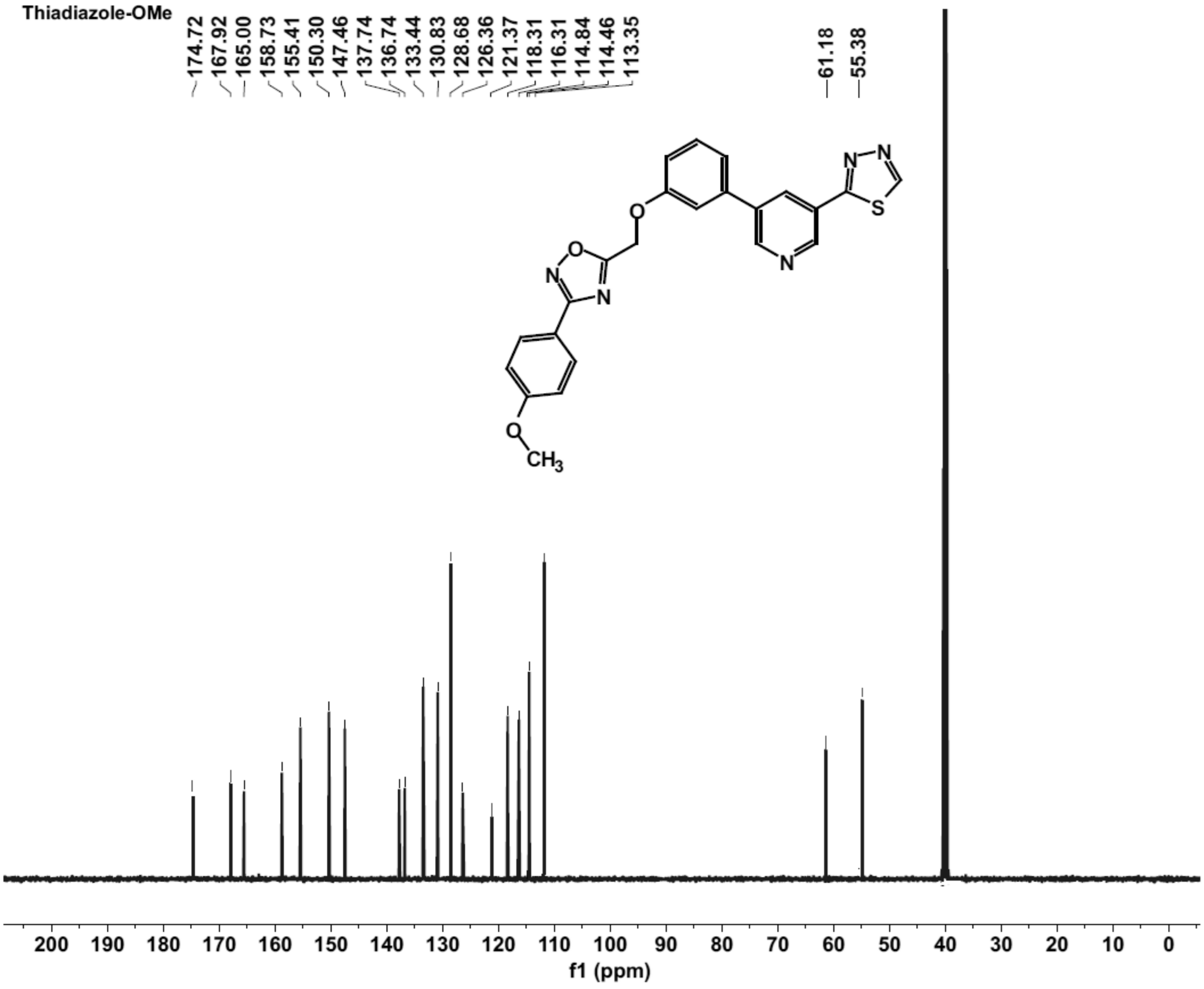

Figure S53: ^{13}C NMR (500 MHz, DMSO-d_6) of compound **22c**

Appendix A

4. EXPERIMENTAL

4.1. Chemistry

Materials and methods

All reagents and solvents were of general purpose or analytical grade and purchased from Sigma Aldrich Ltd, Fisher Scientific, Fluka and Acros. Melting points were determined on Stuart scientific, model SMP3, UK and are uncorrected. Commercial-grade solvents and reagents were used to synthesize the specified compounds. All solvents were dried prior to use and stored over 4 Å molecular sieves, The progress of reactions was checked by TLC (pre-coated TLC sheets, 60G F254, Merk, Germany). UV light was used for detection at 254 nm wavelength (Spectroline, model CM-10, USA). The IR spectra were recorded on a thermoscientific Nicolet IS10 FTIR spectrometer (thermo Fischer scientific, USA) (KBr; ν max in cm^{-1}). ^1H and ^{13}C NMR spectra of key intermediates and final compounds were scanned on Avance-III, High performance FT-NMR spectrum, Bruker biospin international AG Switzeraland at 400 MHz at Faculty of Science, Zagazig University, Egypt and at faculty of pharmacy, Mansoura university, Mansoura, Egypt. Chemical shifts are expressed in δ-values (ppm) relative to TMS as an internal standard, using the appropriate solvent as specified. Coupling constants (*J*) are reported in Hertz (Hz). Deuterium oxide (D_2O) was used for the detection of exchangeable protons. Elemental microanalyses were performed on elemental analyzer model flash 2000 thermo fisher at the regional center for mycology and biotechnology (RCMB), faculty of science, Al-Azhar university, Nasr city, Cairo, Egypt for all new compounds.

4.2. Biological evaluation

4.2.1 Cell Viability assay

MTT assay was performed to investigate the effect of the synthesized compounds on mammary epithelial cells (MCF-10A). The cells were propagated in medium consisting of Ham's F-12 medium/ Dulbecco's modified Eagle's medium (DMEM) (1:1) supplemented with 10% foetal calf serum (GIBCO, UK), 2 mM glutamine, insulin (10 μg/mL), hydrocortisone (500 ng/mL) and epidermal growth factor (20 ng/mL). Trypsin ethylenediamine tetra acetic acid (EDTA) was used to passage the cells after every 2-3 days. 96-well flat-bottomed cell culture plates were used to seed the cells at a density of 104 cells mL^{-1}. The medium was aspirated from all the wells of culture plates after 24 h followed by the addition of synthesized compounds (in 200 μL medium to yield a final concentration of 0.1% (v/v) dimethyl sulfoxide) into individual wells of the plates. Four wells were designated to a single compound. The plates were allowed to incubate at 37°C for 96 h. Afterwards, the medium was aspirated and 3-[4,5-dimethylthiazol-2-yl]-2,5-diphenyltetrazolium bromide (MTT) (0.4 mg/mL) in medium was added to each well and subsequently incubated for 3 h. The medium was aspirated and 150 μL dimethyl sulfoxide (DMSO) was added to each well. The plates were vortexed followed by the measurement of absorbance at 540 nm on a microplate reader. The results were presented as inhibition (%) of proliferation in contrast to controls comprising 0.1% DMSO.

4.2.2. Assay for antiproliferative effect

To explore the antiproliferative potential of compounds propidium iodide fluorescence assay was performed using different cell lines such as: Panc-1 (pancreas cancer cell line) [Panc-1-CTC (RRID:CVCL_VQ69)], MCF-7 (breast cancer cell line) [MCF-7 (RRID:CVCL_0031)], HT-29 (colon cancer cell line) [HT-29 (RRID:CVCL_0320)] and A-549 (epithelial cancer cell line)[A-549 (RRID:CVCL_0023)], respectively. All cell lines were obtained from ATCC (American Type Cell Culture).

To calculate the total nuclear DNA, a fluorescent dye (propidium iodide, PI) is used which can attach to the DNA, thus offering a quick and precise technique. PI cannot pass through the cell membrane, and its signal intensity can be considered as directly proportional to quantity of cellular DNA. Cells whose cell membranes are damaged or have changed permeability are counted as dead ones. The assay was performed by seeding the cells of different cell lines at a density of 3000-7500 cells/well (in 200 µl medium) in culture plates followed by incubation for 24 h at 37 °C in humidified 5% CO2 /95% air atmospheric conditions. The medium was removed; the compounds were added to the plates at 10 µM concentrations (in 0.1% DMSO) in triplicates, followed by incubation for 48 h. DMSO (0.1%) was used as control. After incubation, medium was removed followed by the addition of PI (25 µl, 50 µg/mL in water/medium) to each well of the plates. At -80 °C, the plates were allowed to freeze for 24 h, followed by thawing at 25 ºC. A fluorometer (Polar-Star BMG Tech) was used to record the readings at excitation and emission wavelengths of 530 and 620 nm for each well.

The percentage cytotoxicity of compounds was calculated using the following formula:

$$\% \text{ Cytotoxicity} = \frac{A_C - A_{TC}}{A_C} \times 100$$

Where A*TC*= Absorbance of treated cells and AC= Absorbance of control. Erlotinib was used as positive control in the assay.

4.2.3. EGFR inhibitory assay

Baculoviral expression vectors including pBlueBacHis2B and pFASTBacHTc were used separately to clone 1.6 kb cDNA coding for EGFR cytoplasmic domain (EGFR-CD, amino acids 645–1186). 5′ upstream to the EGFR sequence comprised a sequence that encoded (His)6. Sf-9 cells were infected for 72h for protein expression. The pellets of Sf-9 cells were solubilized in a buffer containing sodium vanadate (100 µM), aprotinin (10 µg/mL), triton (1%), HEPES buffer (50 mM), ammonium molybdate (10 µM), benzamidine HCl (16 µg/mL), NaCl (10 mM), leupeptin (10 µg/mL) and pepstatin (10 µg/mL) at 0°C for 20 min at pH 7.4, followed by centrifugation for 20 min. To eliminate the nonspecifically bound material, a Ni-NTA super flow packed column was used to pass through and wash the crude extract supernatant first with 10 mM and then with 100 mM imidazole. Histidine-linked proteins were first eluted with 250 and then with 500 mM imidazole subsequent to dialysis against NaCl (50 mM), HEPES (20 mM), glycerol (10%) and 1 µg/mL each of aprotinin, leupeptin and pepstatin for 120 min. The purification was performed either at 4 °C or on ice. To record autophosphorylation level, EGFR kinase assay was carried out on the basis of DELFIA/Time-Resolved Fluorometry. The compounds were first dissolved in DMSO absolute, subsequent to dilution to appropriate concentration using HEPES (25 mM) at pH 7.4. Each compound (10 µL) was incubated with recombinant enzyme (10 µL, 5 ng for EGFR, 1:80 dilution in 100 mM HEPES) for 10 min at 25°C, subsequent to the addition of 5X buffer (10

μL, containing 2 mM $MnCl_2$, 100 μM Na_3VO_4, 20 mM HEPES and 1 mM DTT) and ATP-$MgCl_2$ (20 μL, containing 0.1 mM ATP and 50 mM $MgCl_2$) and incubation for 1h. The negative and positive controls were included in each plate by the incubation of enzyme either with or without ATP-$MgCl_2$. The liquid was removed after incubation, and the plates were washed thrice using a wash buffer. The Europium-tagged antiphosphotyrosine antibody (clone PT66) (PerkinElmer, 10 ng/well in a 20 μL final volume, resulting in a final concentration of around 0.5 nM) was added to each well followed by incubation of 1h and then washing of the plates using buffer. The enhancement solution was added to each well and the signal was recorded at excitation and emission wavelengths of 340 at 615 nm. The autophosphorylation percentage inhibition by compounds was calculated using the following equation:

$$100\% - [(negative\ control)/(positive\ control) - (negative\ control)]$$

Using the curves of percentage inhibition of eight concentrations of each compound, IC_{50} was calculated. The majority of signals detected by antiphosphotyrosine antibody were from EGFR because the enzyme preparation contained low impurities.

4.2.4. BRAF^{V600E} inhibitory assay

V^{600E} mutant BRAF kinase assay was performed to investigate the activity of tested compounds against BRAF. Mouse full-length GST-tagged BRAF^{V600E} (7.5 ng, Invitrogen, PV3849) was pre-incubated with drug (1 μL) and assay dilution buffer (4 μL) for 60 min at 25ºC. In assay dilution buffer, a solution (5 μL) containing $MgCl_2$ (30 mM), ATP (200 μM), recombinant human full length (200 ng) and *N*-terminal His-tagged MEK1 (Invitrogen) was added to start the assay, subsequent to incubation for 25 min at 25ºC. The assay was stopped using 5X protein denaturing buffer (LDS) solution (5 μL). To further denature the protein, heat (70° C) was applied for 5 min.

4-12% precast Nu-Page gel plates (Invitrogen) were used to carry out electrophoresis (at 200 V). 10 µL of each reaction was loaded into the precast plates and electrophoresis was allowed to proceed. After completion of electrophoresis, the front part of the precast gel plate (holding hot ATP) was cut and afterwards cast-off. The dried gel was developed using a phosphor screen. A reaction without active enzyme was used as negative control while that containing no inhibitor served as positive control. To study the effect of compounds on cell-based pERK1/2 activity in cancer cells, commercially available ELISA kits (Invitrogen) were used according to manufacturer's instructions.

4.2.5. Bax activation assay

Bring all reagents, except the human Bax-α Standard, to room temperature for at least 30 minutes prior to opening. The human Bax-α Standard solution should not be left at room temperature for more than 10 minutes. All standards, controls and samples should be run in duplicate. Refer to the Assay Layout Sheet to determine the number of wells to be used and put any remaining wells with the desiccant back into the pouch and seal the Ziploc. Store unused wells at 4 °C. Pipet 100 µL of Assay Buffer into the S0 (0 pg/mL standard) wells. Pipet 100 µL of Standards #1 through #6 into the appropriate wells. Pipet 100 µL of the Samples into the appropriate wells. Tap the plate gently to mix the contents. Seal the plate and incubate at room temperature on a plate shaker for 1 hour at ~500 rpm. Empty the contents of the wells and wash by adding 400 µL of wash solution to every well. Repeat the wash 4 more times for a total of 5 washes. After the final wash, empty or aspirate the wells and firmly tap the plate on a lint free paper towel to remove any remaining wash buffer. Pipet 100 µL of yellow Antibody (**E63,** Abcam ab32503, diluted 1:100 with buffer solution) into each well, except the Blank. Seal the plate and incubate at room temperature on a plate shaker for 1 hour at ~500 rpm. Empty the contents of the wells and wash by adding 400 µL of wash solution

to every well. Repeat the wash 4 more times for a total of 5 washes. After the final wash, empty or aspirate the wells and firmly tap the plate on a lint free paper towel to remove any remaining wash buffer. Add 100 µL of blue Conjugate to each well, except the Blank. Seal the plate and incubate at room temperature on a plate shaker for 30 minutes at ~500 rpm. Empty the contents of the wells and wash by adding 400 µL of wash solution to every well. Repeat the wash 4 more times for a total of 5 washes. After the final wash, empty or aspirate the wells and firmly tap the plate on a lint free paper towel to remove any remaining wash buffer. Pipet 100 µL of Substrate Solution into each well. Incubate for 30 minutes at room temperature on a plate shaker at ~500 rpm. Pipet 100 µL Stop Solution to each well. Blank the plate reader against the Blank wells, read the optical density at 450 nm. Calculate the average net Optical Density (OD) bound for each standard and sample by subtracting the average Blank OD from the average OD for each standard and sample. Using linear graph paper, plot the Average Net OD for each standard versus Bax concentration in each standard. Approximate a straight line through the points. The concentration of Bax in the unknowns can be determined by interpolation.

4.2.6. Bcl-2 inhibition assay

Mix all the reagents thoroughly without foaming before use. Wash the microwells twice with approximately 300 µL Wash Buffer per well with thorough aspiration of microwell contents between washes. Take caution not to scratch the surface of the microwells. After the last wash, empty the wells and tap microwell strips on absorbent pad or paper towel to remove excess Wash Buffer. Use the microwell strips immediately after washing or place upside down on a wet absorbent paper for not longer than 15 minutes. Do not allow wells to dry. Add 100 µL of Sample Diluent in duplicate to all standard wells and to the blank wells. Prepare standard (1:2 dilution) in duplicate ranging from 32 ng/mL to 0.5 ng/mL. Add 100 µL of Sample Diluent, in duplicate, to

the blank wells. Add 80 µL of Sample Diluent, in duplicate, to the sample wells. Add 20 µL of each Sample, in duplicate, to the designated wells. Add 50 µL of diluted biotin-conjugate to all wells, including the blank wells. Cover with a plate cover and incubate at room temperature, on a microplate shaker at 100 rpm if available, for 2 hours. Remove plate cover and empty the wells. Wash microwell strips 3 times as described in step 2. Add 100 µL of diluted Streptavidin-HRP to all wells, including the blank wells. Cover with a plate cover and incubate at room temperature, on a microplate shaker at 100 rpm if available, for 1 hour. Remove the plate cover and empty the wells. Wash microwell strips 3 times as described in step 2. Proceed to the next step. Pipette 100 µl of mixed TMB Substrate Solution to all wells, including the blanks. Incubate the microwell strips at room temperature (18° to 25°C) for about 15 minutes, if available on a rotator set at 100 rpm. Avoid direct exposure to intense light. The point, at which the substrate reaction is stopped, is often determined by the ELISA reader. Many ELISA readers record absorbance only up to 2.0 O.D. Therefore, the color development within individual microwells must be watched by the person running the assay and the substrate reaction stopped before positive wells are no longer properly detectable. Stop the enzyme reaction by quickly pipetting 100 µL of Stop Solution into each well, including the blank wells. It is important that the Stop Solution is spread quickly and uniformly throughout the microwells to completely inactivate the enzyme. Results must be read immediately after the Stop Solution is added or within one hour if the microwell strips are stored at 2 - 8°C in the dark. Read the absorbance of each microwell on a spectrophotometer using 450 nm as the primary wavelength.

4.2.7. Caspase-3 activation assay

Allow all reagents to reach room temperature before use. Gently mix all liquid reagents prior to use. Determine the number of 8-well strips needed for the assay. Insert these in the frame(s) for current use. Add 100 µl of the Standard Diluent Buffer (1X Cell Extraction Buffer PTR) to the zero standard wells. Well(s) reserved for chromogen blank should be left empty. Add 100 µl of standards and controls or diluted samples to the appropriate microtiter wells. The sample dilution chosen should be optimized for each experimental system. Tap gently on side of plate to mix. Cover wells with *plate cover* and incubate for 2 hours at room temperature. Thoroughly aspirate or decant solution from wells and discard the liquid, Wash wells 4 times. Pipette 100 µl of Caspase-3 (Active) Detection Antibody (**PerkinElmer Alpha LISA** system, Europium tag, Alpha beads, final concentrations of 20 µg/mL), solution into each well except the chromogen blank(s). Tap gently on the side of the plate to mix. Cover plate with plate cover and incubate for 1 hour at room temperature. Thoroughly aspirate or decant solution from wells and discard the liquid, Wash wells 4 times. Add 100 µl Anti-Rabbit IgG HRP Working Solution to each well except the chromogen blank(s). Prepare the working dilution as described in Preparing IgG HRP. Cover wells with the plate cover and incubate for 30 minutes at room temperature. Thoroughly aspirate or decant solution from wells and discard the liquid. Wash wells 4 times. Add 100 µl of Stabilized Chromogen to each well. The liquid in the wells will begin to turn blue. Incubate for 30 minutes at room temperature and in the dark. The incubation time for chromogen substrate is often determined by the microtiter plate reader used. Many plate readers have the capacity to record a maximum optical density (O.D.) of 2.0. The O.D. values should be monitored, and the substrate reaction stopped before the O.D. of the positive wells exceeds the limits of the instrument. The O.D. values at 450 nm can only be read after the Stop Solution has been added to each well. If

using a reader that records only to 2.0 O.D., stopping the assay after 20 to 25 minutes is suggested. Add 100 µl of Stop Solution to each well. Tap side of plate gently to mix. The solution in the wells should change from blue to yellow. Read the absorbance of each well at 450 nm having blanked the plate reader against a chromogen blank composed of 100 µl each of Stabilized Chromogen and Stop Solution. Read the plate within 2 hours after adding the Stop Solution. Use a curve fitting software to generate the standard curve. A four-parameter algorithm provides the best standard curve fit. Read the concentrations for unknown samples and controls from the standard curve. Multiply value(s) obtained for sample(s) by the appropriate dilution factor to correct for the dilution in step 3. Samples producing signals greater than that of the highest standard should be diluted in Standard Diluent Buffer and reanalyzed.

4.2.8. Caspase-8/9 activation assay

Cells were obtained from American Type Culture Collection, cells were grown in RPMI 1640 containing 10% fetal bovine serum at 37°C, stimulated with the compounds to be tested for caspase 8/9, and lysed with Cell Extraction Buffer. This lysate was diluted in Standard Diluent Buffer over the range of the assay and measured for human active caspase-8/9 content. (Cells are Plated in a density of 1.2 – 1.8 × 10,000 cells/well in a volume of 100µl complete growth medium + 100 ul of the tested compound per well in a 96-well plate for 24 hours before the enzyme assay). The absorbance of each microwell was read on a spectro-photometer at 450 nm. A standard curve is prepared from 7 human Caspase-8/9 standard dilutions and human Caspase-8/9 concentration determined.

4.2.9. TNF-α and IL6 a determination

The effect of compounds **20c** and **21c** on the expression of TNF- *α* and IL-6 were determined using of q RT-PCR technique. By using q RTPCR (reference), the amount of immunomodulatory proteins TNF-α and IL-6 in control and compounds **20c-** and **21c**-treated HCT-116 cells was measured (at the IC_{50} concentrations). Total RNA was extracted from 10k-treated HepG2 cells and vehicle treated control (0.01% DMSO) in accordance with the manufacturer's instructions (RNeasy mini kit, Qiagen, Germany). Following RNA extraction, the Revert Aid First Strand cDNA Synthesis kit (Thermo Scientific, USA) was used to create cDNA. The Rotor-Gene Q real-time PCR thermal cycler was used to amplify target cDNA for apoptosis markers and GAPDH [as a normalization (housekeeping) gene] using one step RT-PCR SYBR® Green kit Master Mix (Bio-Rad Laboratories, USA). Two microliters of cDNA were combined with one microliter of forward primer, one microliter of reverse primer, ten microliters of master mixture, and twenty microliters of nuclease-free water to complete the reaction volume. Every experiment was carried out three times.

Quantitative Real Time Reverse-Transcriptase PCR (qRT-PCR) primer sequences

TNF-α : R 5'- ATGGGCTACAGGCTTGTCACTC -3'.

TNF-α: F 5’- CTCTTCTGCCTGCTGCACTTTG -3’,

IL6 : R 5'- TTCTGCCAGTGCCTCTTTGCTG -3

IL6 : F 5’- AGACAGCCACTCACCTCTTCAG -3’,

GAPDH : R 5’- ACCACCCTGTTGCTGTAGCCAA-3’

GAPDH : F 5’- GTCTCCTCTGACTTCAACAGCG-3’

4.3. Docking studies

The crystal structures of EGFR (PDB code: 1M17) and $BRAF^{V600E}$ (PDB code: 3OG7) were downloaded from the Protein Data Bank. Structures of compounds **20c**, and **21c** were drawn and optimized using Avogadro molecular editors. The proteins were prepared using Autodock tools where the co-crystallized ligands and water molecules were removed then kollman charges and polar hydrogens were added. Autodock vina was used for molecular docking and the best docking poses were visualized using Discovery Studio Visualizer.

4.4. Statistical analysis

Computerized Prism 5 program was used to statistically analyzed data using one-way ANOVA test followed by Tukey's as post ANOVA for multiple comparison at $P \leq .05$. Data were presented as mean ± SEM.
